# Detecting multiple generalized change-points by isolating single ones

**DOI:** 10.1007/s00184-021-00821-6

**Published:** 2021-05-24

**Authors:** Andreas Anastasiou, Piotr Fryzlewicz

**Affiliations:** 1 Department of Mathematics and Statistics, University of Cyprus, P.O. Box 20537, 1678 Nicosia, Cyprus; 2 Department of Statistics, The London School of Economics and Political Science, Columbia House, Houghton Street, London, WC2A 2AE UK

**Keywords:** Segmentation, Symmetric interval expansion, Threshold criterion, Schwarz information criterion, SDLL

## Abstract

We introduce a new approach, called Isolate-Detect (ID), for the consistent estimation of the number and location of multiple generalized change-points in noisy data sequences. Examples of signal changes that ID can deal with are changes in the mean of a piecewise-constant signal and changes, continuous or not, in the linear trend. The number of change-points can increase with the sample size. Our method is based on an isolation technique, which prevents the consideration of intervals that contain more than one change-point. This isolation enhances ID’s accuracy as it allows for detection in the presence of frequent changes of possibly small magnitudes. In ID, model selection is carried out via thresholding, or an information criterion, or SDLL, or a hybrid involving the former two. The hybrid model selection leads to a general method with very good practical performance and minimal parameter choice. In the scenarios tested, ID is at least as accurate as the state-of-the-art methods; most of the times it outperforms them. ID is implemented in the R packages **IDetect** and **breakfast**, available from CRAN.

**Supplementary Information:**

The online version supplementary material available at 10.1007/s00184-021-00821-6.

## Introduction

Change-point detection is an active area of statistical research that has attracted a lot of interest in recent years. Our work’s focus is on a posteriori change-point detection, where the aim is to estimate the number and locations of certain changes in the behaviour of the data. We work in the model

1 $$\begin{aligned} X_t = f_t + \sigma \epsilon _t, \quad t=1,2,\ldots ,T, \end{aligned}$$

where $X_t$ are the observed data and $f_t$ is a one-dimensional, deterministic signal with structural changes at certain points. Two examples are: change-points in the level when $f_t$ is seen as piecewise-constant, and change-points in the first derivative when $f_t$ is piecewise-linear. We highlight, however, that our methodology and analysis apply to more general scenarios, for instance the detection of knots in a piecewise polynomial signal of order *k*, where *k* is not necessarily equal to zero (piecewise-constant mean) or one (piecewise-linear mean). The number *N* of change-points as well as their locations $r_1, r_2, \ldots , r_N$ are unknown and our aim is to estimate them. In addition, *N* can grow with *T*. The random variables $\epsilon _t$ in (1) have mean zero and variance one; further assumptions will be given in Sect. 3.2.

When $f_t$ is assumed to be piecewise-constant, the existing change-point detection techniques are mainly split into two categories based on whether the change-points are detected all at once or one at a time. The former category mainly includes optimization-based methods, in which the estimated signal is chosen based on its least squares or log-likelihood fit to the data, penalized by a complexity rule in order to avoid overfitting. The most common example of a penalty function is the Schwarz Information Criterion (SIC); see Yao (1988) for details. To solve the implied penalization problem, dynamic programming approaches, such as the Segment Neighborhood (SN) and Optimal Partitioning (OP) methods of Auger and Lawrence (1989) and Jackson et al. (2005), have been developed. In an attempt to improve on OP’s computational cost, Killick et al. (2012) introduce the PELT method, based on a pruning step applied to OP’s dynamic programming approach. A non-parametric adaptation of PELT is given in Haynes et al. (2017). Rigaill (2015) introduces an improvement over classical SN algorithms, through a pruning approach called PDPa, while Maidstone et al. (2017) give two algorithms by combining ideas from PELT and PDPa. Frick et al. (2014) propose the simultaneous multiscale change-point estimator (SMUCE) for the change-point problem in the case of exponential family regression; solving an optimization problem is also required. The FDRSeg method of Li et al. (2016) is a combination of False Discovery Rate (FDR) control and global segmentation methods in a multiscale way; the change-points are again detected all at once.

In the latter category, in which change-points are detected one at a time, a popular method is binary segmentation, which performs an iterative binary splitting of the data on intervals determined by the previously obtained splits. Vostrikova (1981) introduces and proves the validity of binary segmentation in the setting of change-point detection for piecewise-constant signals. The main advantages of binary segmentation are its conceptual simplicity and low computational cost. However, at each step of the algorithm, binary segmentation looks for a single change-point, which leads to its suboptimality in terms of accuracy, especially for signals with frequent change-points. Some variants of binary segmentation that work towards solving this issue are the Circular Binary Segmentation (CBS) of Olshen et al. (2004), the Wild Binary Segmentation (WBS) of Fryzlewicz (2014) as well as its second version (WBS2) of Fryzlewicz (2020), the Narrowest-Over-Threshold (NOT) method of Baranowski et al. (2019), and the Seeded Binary Segmentation (SeedBS) of Kovács et al. (2020). CBS searches for at most two change-points at each step. Instead of initially calculating the contrast value for the whole data sequence, WBS and NOT are based on a random draw of subintervals of the domain of the data, on which an appropriate statistic is tested against a threshold. The draw of all the subintervals takes place at the beginning of the algorithm. In contrast, WBS2 draws first only a small number, ${\tilde{M}}$, of data subsamples. It then uses the first change-point candidate to split the data into two parts, and again recursively draws the same number ${\tilde{M}}$ of subsamples to the left and to the right of this change-point candidate, and so on. A major difference between WBS and WBS2 is that the latter adaptively decides where to recursively draw the next subsamples, based on the change-point candidates detected so far; this adds to the detection power of the method. SeedBS is an approach, similar to WBS and NOT, that relies instead on a deterministic construction of background intervals in which single change points are searched. Apart from binary-segmentation-related approaches, the category in which the change-points are detected one at a time also includes methods that control the False Discovery Rate. For instance, the “pseudo-sequential” (PS) procedure of Venkatraman (1992), as well as the CPM method of Ross (2015) are based on an adaptation of online detection algorithms to a posteriori situations and work by bounding the Type I error rate of falsely detecting change-points. Some methods do not fall in either category. For example, the tail-greedy algorithm in Fryzlewicz (2018) achieves a multiscale decomposition of the data using Unbalanced Haar wavelets in an agglomerative way. In addition, Eichinger and Kirch (2018) use moving sum (MOSUM) statistics in order to detect multiple change-points. For a more thorough review of the literature on the detection of multiple change-points in the mean of univariate data sequences, see Cho and Kirch (2020) and Yu (2020). Truong et al. (2020) also present a survey of various a posteriori change-point detection algorithms; the focus is, however, on multivariate time series.

Beyond the piecewise-constant signal model, existing methods mainly minimize the residual sum of squares taking into account a penalty, with the most common being the SIC. This is used in Bai and Perron (1998), in the trend filtering (TF) approach (Kim et al. 2009; Tibshirani 2014), and in the dynamic programming algorithm CPOP (Maidstone et al. 2019). Friedman (1991) introduces the Multivariate Adaptive Regression Splines (MARS) method for regression analysis based on splines with the number and the location of the knots being determined by the data. Spiriti et al. (2013) propose two methods for optimizing knot locations in spline smoothing, where either the number of knots is fixed or an upper bound for it needs to be given. The NOT approach (Baranowski et al. 2019) detects change-points one at a time in various scenarios including piecewise-linear mean signals.

In general, change-point detection becomes easier in situations where there is at most one change-point to be detected in a given interval; in such cases the detection power of the contrast function (more details are in Sect. 3.2) is maximised. Therefore, it makes sense to decouple the multiple change-point detection problem into many single change-point detections. To achieve this, we propose a generic technique, Isolate-Detect (ID), for generalized change-point detection in various different structures, such as piecewise-constant or piecewise-linear signals. The concept behind ID is simple and is split into two stages; firstly, the isolation of each of the true change-points within subintervals of the domain $[1,2,\ldots ,T]$, and secondly their detection. From now on, the terms *subinterval* and *interval* will be used interchangeably. Although a detailed explanation of our methodology is provided in Sect. 3.1, the basic idea is that for an observed data sequence of length *T* and with $\lambda _T$ a positive constant, ID first creates two ordered sets of $K = \left\lceil T/\lambda _T \right\rceil$ right- and left-expanding intervals as follows. The *j*th right-expanding interval is $R_j = [1,\min \left\{ j\lambda _T, T\right\} ]$, while the *j*th left-expanding interval is $L_{j} = [\max \left\{ 1,T - j\lambda _T + 1\right\} ,T]$. We collect these intervals in the ordered set $S_{RL} = \left\{ R_1, L_1, R_2, L_2, \ldots ,R_K,L_K\right\}$. For a suitably chosen contrast function (more details are in Sect. 3.2), ID identifies the point with the maximum contrast value in $R_1$. If its value exceeds a threshold, denoted by $\zeta _T$, then it is taken as a change-point. If not, then the next interval in $S_{RL}$ is tested. Upon detection, ID makes a new start from the end-point (or start-point) of the right- (or left-) expanding interval where the detection occurred. Upon correct choice of $\zeta _T$, ID ensures that we work on intervals with at most one change-point, which was our aim.

We would like to highlight the importance of the change-point isolation aspect present in our method as explained in the previous paragraph. There are various advantages. First, it enables detection in higher-order polynomial signals. Second, it is carried out in a fixed and systematic way, which eliminates any randomness in the selection of the intervals and, by extension, in the final results. Third, the way the isolation is carried out in ID makes it quicker than other localisation-focused algorithms, such as NOT, due to the fact that it needs to work on fewer intervals; more details on this advantage of our proposed methodology are in Sect. 4.1. We note here that, even though the default methodology described in Fryzlewicz (2014) and Baranowski et al. (2019) is based on the construction of random intervals, the same approaches can be applied to a fixed grid of intervals. However, as noted in Kovács et al. (2020), the latter implementation can be quite slow. Fourth, the pseudo-sequential nature of the attempted isolation, makes our proposed methodology suitable for online change-point detection. This is one of the various different ways that ID is different from existing techniques in the literature which also attempt change-point isolation; a more thorough comparison with seemingly similar, but still different, methods is given in the next section.

The paper is organized as follows. Section 2 is a motivating illustration of our proposed method through examples. Section 3 gives a formal explanation of the ID methodology along with two different scenarios of use and the associated theory. In Sect. 4, we first discuss the computational aspects of ID and the choice of parameter values. ID variants which lead to improved practical performance are also explained. In Sect. 5, we provide a thorough simulation study to compare ID with state-of-the-art methods. Real-life data examples are provided in Sect. 6. The paper is concluded with reflections on the proposed method. The theoretical, as well as practical, merits and weaknesses of ID when compared against state-of-the-art methods are discussed throughout the paper. However, for the sake of clarity these are also brought together in Sect. 7. ID is implemented in the R packages **IDetect** and **breakfast**, available from CRAN.

## Motivating illustration of Isolate-Detect

The fact that each change-point is sequentially detected using an interval that contains no other change-points leads to high detection power, especially in difficult structures, such as limited spacings between consecutive change-points and/or higher-order piecewise-polynomial signals. Two examples follow in order to make clear the importance of the isolation step and to illustrate the power of ID compared to other change-point detection methods (some of those also attempt localisation) in capturing even small movements in the data that are close to each other. Table 1 provides results on 100 replications of the continuous piecewise-linear signal (S1) and the piecewise-constant signal (S2), where

1. $T = 5200$, with 21 change-points in the slope at locations $100, 140, \cdots , 900$. The standard deviation is $\sigma =0.25$;
2. $T = 5200$, with 21 change-points in the mean at locations $100, 105, \cdots , 200$. The standard deviation is $\sigma =0.1$.

**Table 1:** Distribution of ${\hat{N}} - N$ over 100 simulated data sequences from (S1)

Signal	Method						MSE
		$${\hat{N}} - N$$					
		$$\le - 15$$	$$(-15, -5] $$	$$[-4,4]$$	[5, 15)	$$\ge 15$$	
(S1)	**ID**	0	0	**100**	0	0	$13 {\times } 10^{-5}$
	NOT	5	86	9	0	0	$141 {\times } 10^{-5}$
	MARS	100	0	0	0	0	$284 \times 10^{-5}$
(S2)	**ID**	0	1	**97**	2	0	$94 \times 10^{-5}$
	NOT	100	0	0	0	0	$485 \times 10^{-5}$
	PELT	78	22	0	0	0	$437 \times 10^{-5}$
	WBS	27	71	2	0	0	$413 \times 10^{-5}$

The average MSE is also given

**Fig. 1:**
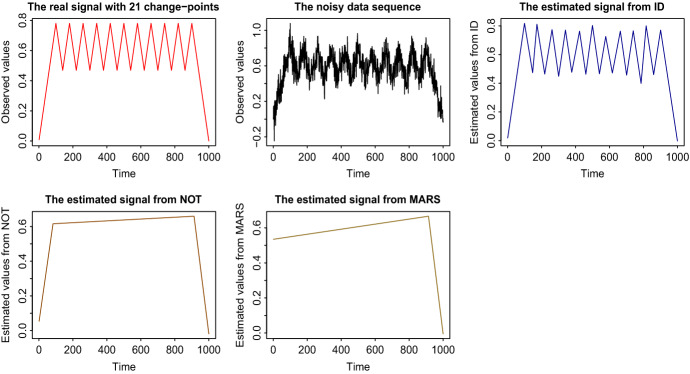
Results (up to $t = 1000$) on estimated signals obtained by different change-point detection methods. Top row: the true signal (S1) and the data sequence, and the estimated signal using ID. Bottom row: The estimated signals from NOT, and MARS

**Fig. 2:**
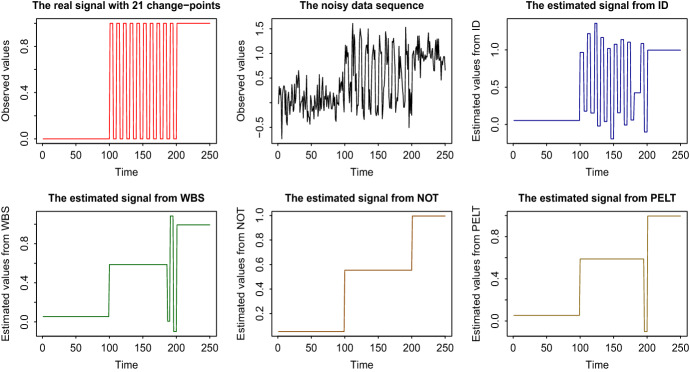
Results (up to $t=250$) on estimated signals obtained by different change-point detection methods. Top row: the true signal (S2), the data sequence, and the estimated signal using ID. Bottom row: The estimated signals from WBS, NOT, and PELT

As a measure of the accuracy of the estimated number we give ${\hat{N}} - N$, while as a measure of the accuracy of the detected locations, we give Monte-Carlo estimates of the mean square error, $\mathrm{MSE} = T^{-1}\sum _{t=1}^{T}{{\mathbb {E}}} \left( {\hat{f}}_t - f_t\right) ^2$. The methods compared are ID, NOT, and MARS for (S1) and ID, WBS, NOT, and PELT for (S2). For the ID related results in Table 1, we used the hybrid version of ID explained in Sect. 4.4. The choice of the parameters is described in Sect. 4.2. As already mentioned, WBS and NOT also work on subintervals of the data, chosen though in a completely different manner than in ID. More comparative simulation and real-life studies will be given in Sects. 5 and 6, respectively. We notice from Table 1 that ID offers an important increase in the change-point detection power, especially under limited spacings between consecutive change-points. Figures 1 and 2 give a graphical representation of the results for the first out of the 100 repetitions for signals (S1) and (S2), respectively. For better presentation of the results, in (S1) the signals are presented up to $t=1000$, since after $t = 900$ there is no change-point and in all methods the estimated signal continues linearly beyond that point. For the same reason, in Fig. 2 which is related to (S2), the results are presented up to $t = 250$. The NOT and WBS methods also operate on sub-intervals of the data. However, the nature of the fixed, certain (we can expand one data point at each time), localization in ID means that it is of an order of magnitude faster than the aforementioned methods, which have high computational cost that increases linearly with the number of the randomly drawn intervals. This is an issue of fundamental importance, especially in signals with a large number of change-points, in which NOT and WBS need to increase the number *M* of intervals drawn. However, doing this also increases the computational cost. More specifically, one could try and draw all possible combinations of start- and end-points of the intervals; however, the computational complexity turns out to be cubic in *T*. In contrast, due to the explained interval expansion approach, in ID no choice of *M* is required, which leads to better practical performance with more predictable execution times, while at the same time ID examines all possible change-point locations. We recall that unlike ID and NOT, the principle of WBS does not extend to models other than piecewise-constant. To be more precise, this generality of Isolate-Detect with respect to its applicability in many different signal structures is a main distinction between our method and recently published competing methods which, with the exception of NOT, have been developed to cover only the detection of level-changes.

## Methodology and theory

### Methodology

**Fig. 3:**
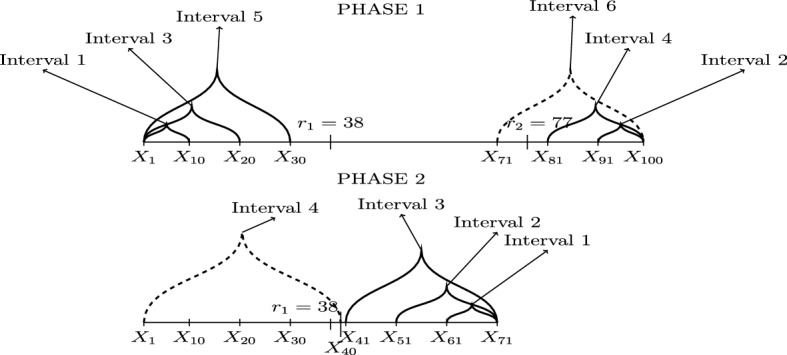
An example with two change-points; $r_1=38$ and $r_2 = 77$. The dashed line is the interval in which the detection took place in each phase

The model is given in (1) and the unknown number, *N*, of change-points $r_j$ can possibly grow with *T*. Let $r_{0} = 0$ and $r_{N+1} = T$ and let $\delta _T = \min _{j=1,2,\ldots ,N+1} \left| r_{j} - r_{j-1}\right|$. For clarity of exposition, we start with a simple example before providing a more thorough explanation of how ID works. Figure 3 covers a specific case of two change-points, $r_1=38$ and $r_2=77$. We will be referring to Phases 1 and 2 involving six and four intervals, respectively. These are clearly indicated in the figure and they are only related to this specific example, as for cases with more change-points we would have more such phases. At the beginning, $s=1$, $e=T=100$, and we take $\lambda _T = 10$ (how to choose $\lambda _T$ will be described in Sect. 4.2). Suppose the threshold $\zeta _T$ has been chosen well enough (more details in Sect. 4.2) so that $r_2$ gets detected in $\left\{ X_{s^*}, X_{s^*+1},\ldots ,X_{e}\right\}$, where $s^*=71$. After the detection, *e* is updated as the start-point of the interval where the detection occurred; therefore, $e=71$. In Phase 2 indicated in the figure, ID is applied in $[s,e]=[1,71]$. Intervals 1, 3 and 5 of Phase 1 will not be re-examined in Phase 2 and $r_1$ gets, upon a good choice of $\zeta _T$, detected in $\left\{ X_{s}, X_{s+1},\ldots ,X_{e^*}\right\}$, where $e^*=40$. After the detection, *s* is updated as the end-point of the interval where the detection occurred; therefore, $s=40$. Our method is then applied in $[s,e] = [40,71]$; supposing there is no interval $[s^*,e^*]\subseteq [40,71]$ on which the contrast function value exceeds $\zeta _T$, the process will terminate.

We now describe ID more generically. For each change-point, $r_j$, ID works in two stages: Firstly, we isolate $r_j$ in an interval that contains no other change-point. To ensure this, the expansion parameter $\lambda _T$ can be taken to be as small as equal to 1. If $\lambda _T > 1$, then isolation is guaranteed with high probability. Theoretically for large *T*, the chosen value for $\lambda _T$ (this typically will be small; see Sect. 4.2 for more details) is guaranteed to be smaller than the minimum distance $\delta _T$ (which has to grow with *T*) between two consecutive change-points and isolation will be guaranteed. For an explanation on the rate of $\delta _T$ with respect to the sample size *T*, see the discussion that follows Theorem 1. (Of course when asymptotics is put aside, in finite samples anything can happen, and in some configurations no method can be guaranteed to detect change-points if they are arbitrarily close.) The second stage is to detect $r_j$ through the use of an appropriate contrast function. This function is, from now on, denoted by $C_{s,e}^b({\varvec{X}})$, and it is defined for any integer triple (*s*, *e*, *b*), with $1\le s \le b < e \le T$. Heuristically, the value of $C_{s,e}^b({\varvec{X}})$ is small if *b* is not a change-point and large otherwise. In piecewise-constant signals, the contrast function reduces to the absolute value of the CUSUM statistic defined in (4), while for continuous, piecewise-linear signals, the contrast function is given in Sect. 3.2. For the better understanding of the method, we provide its step-by-step simple outline through pseudocode, followed by a succinct narrative of the purpose of each step. The threshold to be used, in order to decide if a change has occurred at a specific data point, is denoted by $\zeta _T$. Practical choices for $\lambda _T$ and $\zeta _T$ are given in Sect. 4.2. For $K=\lceil T/\lambda _T\rceil$, let $c_{j}^r = j\lambda _T$ and $c_{j}^l = T - j\lambda _T + 1$ for $j=1,2,\ldots , K-1$, while $c_{K}^r = T$ and $c_{K}^l = 1$. For a generic interval [*s*, *e*], define the sequences

2 $$\begin{aligned} \mathrm{R}_{s,e} = \left[ c_{k_1}^r, c_{k_1+1}^r, \ldots ,e \right] , \quad \mathrm{L}_{s,e} = \left[ c_{k_2}^l, c_{k_2+1}^l, \ldots , s\right] , \end{aligned}$$

where $k_1 := \mathrm{argmin}_{j \in \left\{ 1,2\ldots ,K \right\} }\left\{ j\lambda _T > s \right\}$ and $k_2 := \mathrm{argmin}_{j\in \left\{ 1,2\ldots ,K \right\} }\lbrace T-j\lambda _T+1 < e \rbrace$. Denoting by |*A*|, the cardinality of any sequence *A*, and by *A*(*j*) its *j*th element, the pseudocode of the main function is as below:

***Pseudocode explaining the proposed ID algorithm***

**function** **I**SOLATE**D**ETECT($s, e, \lambda _T, \zeta _T$) 
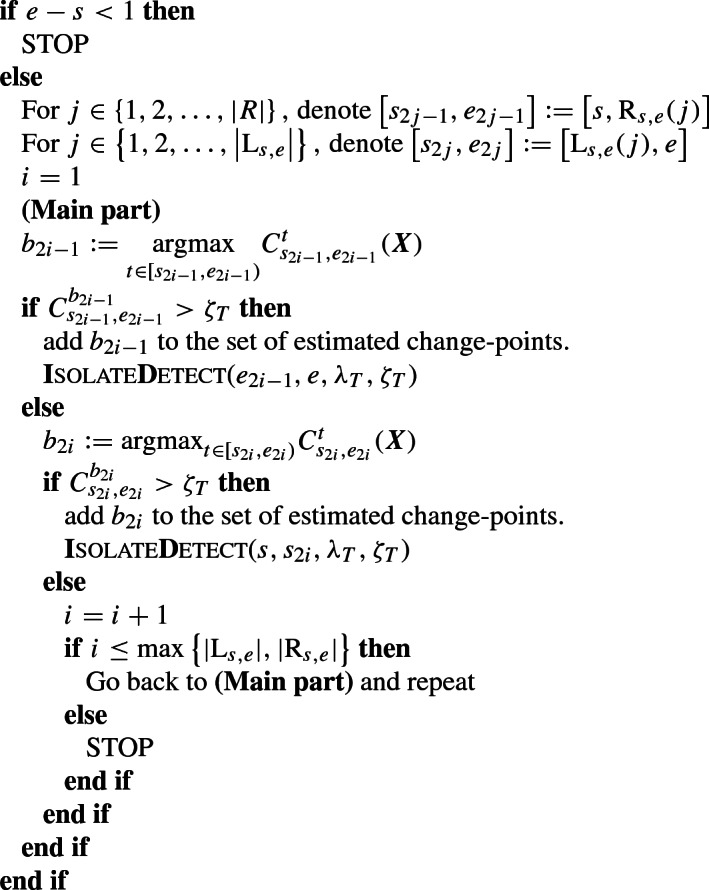
**end function**

A brief explanation of the pseudocode follows. With *K* already defined above, the intervals $[s_1,e_1], [s_2,e_2], \ldots , [s_{2K},e_{2K}]$ are those used for the isolation step. Notice that in the odd intervals $[s_1, e_1], [s_3, e_3], \ldots , [s_{2K - 1}, e_{2K - 1}]$ the start-point is fixed, unchanged, and equal to *s*, meaning that $s_1 = s_3 = \cdots = s_{2K-1} = s$. In the even intervals $[s_2, e_2], [s_4, e_4], \ldots , [s_{2K}, e_{2K}]$, it is the end-point that is kept fixed and equal to *e*, meaning that $e_2 = e_4 = \cdots = e_{2K} = e$. The process will follow until there are intervals to check. The term “expanding intervals” that is used throughout the paper is due to this one-sided expansion (of magnitude $\lambda _T$) of the intervals. The pseudocode makes it also clear that ID is looking for change-points interchangeably in *right*- and *left-expanding* intervals which, with high probability, contain at most one change-point. The Isolate-Detect procedure is launched by the call **I**SOLATE**D**ETECT($1, T, \lambda _T, \zeta _T$).

The idea of a-posteriori change-point detection, in which change-points are detected sequentially, has appeared previously in the literature. The PS method of Venkatraman (1992) studies the multiple change-point detection problem for the case of piecewise-constant mean signals, as well as for changes in the rate of an exponential process. The CPM method of Ross (2015) treats change-point detection in the mean or variance of a sequence of random variables when their distribution is known. In addition, CPM can be used for distributional changes. Fang and Siegmund (2020), in a work completed after the first version of the current paper appeared on arXiv, search for significant change-points in settings such as piecewise-linear, and one of their algorithms, labelled Seq, bears some resemblance to ID; we note, however, that in addition to some algorithmic differences our aim is different as we focus on consistent estimation while Fang and Siegmund (2020) on testing.

ID is conceptually and in practice different from these methods in a number of ways related to the threshold choice, the construction of the estimated change-point locations as well as the way PS, CPM, and Seq restart upon detection. Furthermore, ID’s isolation technique does not appear in CPM. By contrast, we use this isolation property of ID as a device enabling its use in piecewise-(higher-order-) polynomial models. Indeed, as shown in Baranowski et al. (2019), fast segmentation of signals of the latter type is difficult to achieve unless any change-point present can be isolated away from neighbouring change-points before detection is performed, which is exactly what ID sets out to do. In particular, this paper demonstrates the use of ID in continuous piecewise-linear models. A comparison between the performance of ID and that of state-of-the-art methods is given in Sect. 5.

### Theoretical behavior of ID

The assumption of the random sequence $\left\{ \epsilon _t\right\} _{t=1,2,\ldots ,T}$ being independent and identically distributed (i.i.d.) from the Gaussian distribution is widely used in the literature. In this paper, the Gaussianity assumption is only made for technical convenience with respect to the proofs of Theorems 1 and 2. Relaxing both the Gaussianity and the independence assumptions in order to have time-dependent errors is a more complicated issue in terms of theory development. Recently, Dette et al. (2018) have attempted to treat this issue, specifically for the SMUCE approach of Frick et al. (2014), using a reliable estimate for the long run variance, $\sigma _*^2 := \sum _{k \in {\mathbb {Z}}}\mathrm{Cov}(\epsilon _0, \epsilon _k)$, of the error distribution, which is not necessarily Gaussian.

Apart from the well-studied i.i.d. Gaussian noise structure, Isolate-Detect is explored under a variety of settings including i.i.d. non-Gaussian (see Sect. 4.5), and auto-correlated noise structures; see Fearnhead and Rigaill (2020) who conclude that “IDetect has very strong performance for many scenarios when either we have auto-correlated or heavy-tailed noise”.

If the standard deviation, $\sigma$, of $\epsilon _t$ is unknown, then we need to estimate it and in the cases of independent errors with the signal being piecewise-constant or piecewise-linear, $\sigma$ can be estimated via the Median Absolute Deviation (MAD) method proposed in Hampel (1974). For ${\varvec{x}} = (x_1, x_2, \ldots , x_T)$, the proposed estimator, denoted by ${\hat{\sigma }} := C\times \mathrm{median}\left| {\varvec{x}} - \mathrm{median}({\varvec{x}})\right|$, has been shown to be, for $C = 1.4826$, a consistent estimator of the population standard deviation $\sigma$ in the case of Gaussian data (Rousseeuw 1993). It is very robust as evidenced by its bounded influence function and its 50% breakdown point. For simplicity, let $\sigma = 1$, and (1) becomes

3 $$\begin{aligned} X_t = f_t + \epsilon _t, \quad t=1,2,\ldots ,T. \end{aligned}$$

With $r_0 = 0$ and $r_{N+1} = T$, and for $j=1,2,\ldots ,N+1$, we examine the theoretical behaviour of ID in the following two illustration cases:

**Piecewise-constant signals:** $f_t = \mu _j$ for $t = r_{j-1}+1,\ldots ,r_j$, and $f_{r_j}\ne f_{r_j+1}$.

**Continuous, piecewise-linear signals:** $f_{t} = \mu _{j,1} + \mu _{j,2}t$, for $t = r_{j-1} + 1, \ldots , r_{j}$ with the additional constraint of $\mu _{k,1} + \mu _{k,2}r_{k} = \mu _{k+1,1} + \mu _{k+1,2}r_{k}$ for $k=1,2,\ldots ,N$. The change-points, $r_k$, satisfy $f_{r_k-1} + f_{r_k+1}\ne 2f_{r_k}$.

The above scenarios are only examples of settings in which the ID methodology can be applied. The isolation aspect of the method allows its application to various different cases, such as the estimation of the number and the position of knots in piecewise polynomial signals (with or without the continuity constraint).

**Piecewise-constant signals.** Under piecewise-constancy, the contrast function used is the absolute value of the CUSUM statistic, the latter being

4 $$\begin{aligned} {\tilde{X}}_{s,e}^b = \sqrt{\frac{e-b}{n(b-s+1)}}\sum _{t=s}^{b}X_t - \sqrt{\frac{b-s+1}{n(e-b)}}\sum _{t=b+1}^{e}X_t, \end{aligned}$$

where $1\le s \le b < e\le T$ and $n=e-s+1$. Under the i.i.d. Gaussian framework used for the theoretical results presented in this paper, it can be shown that $\mathrm{argmax}_b\left| {\tilde{X}}_{s,e}^b\right| = \mathrm{argmax}_b {\mathcal {R}}_{s,e}^b({\varvec{X}})$, where ${\mathcal {R}}_{s,e}^b({\varvec{X}})$ is the generalized log-likelihood ratio statistic for all potential single change-points within [*s*, *e*]. For the main result of Theorem 1, we also make the following assumption.

1. The minimum distance, $\delta _T$, between two change-points and the minimum magnitude of jumps, ${\underline{f}}_T$, are connected by $\sqrt{\delta _T}{\underline{f}}_T \ge {\underline{C}}\sqrt{\log T}$, for a large enough constant ${\underline{C}}$.

The number of change-points, *N*, is assumed to be neither known nor fixed. It can grow with *T* and the only indirect assumption on *N* is due to the minimum distance, $\delta _T$, between two change-points in the sense that $N + 1\le T/\delta _T$. Below, we give the theoretical result for the consistency of the number and location of the estimated change-points. The proof is in Section 8 of the supplementary material.

#### Theorem 1

Let $\left\{ X_t \right\} _{t=1,2,\ldots ,T}$ follow model (3), with $f_t$ being a piecewise-constant signal and assume that the random sequence $\left\{ \epsilon _t\right\} _{t=1,2,\ldots ,T}$ is independent and identically distributed (i.i.d.) from the normal distribution with mean zero and variance one and also that (A1) holds. Let *N* and $r_j, j=1,2,\ldots ,N$ be the number and locations of the change-points, while ${\hat{N}}$ and ${\hat{r}}_j, j=1,2,\ldots ,{\hat{N}}$ are their estimates sorted in increasing order. In addition, $\varDelta _j^f = \left| f_{r_j+1} - f_{r_j}\right|$, $j=1,2,\ldots , N$. Then, there exist positive constants $C_1, C_2, C_3, C_4$, which do not depend on *T*, such that for $C_1\sqrt{\log T}\le \zeta _T < C_2\sqrt{\delta _T}{\underline{f}}_T$ and for a sufficiently large *T*, we obtain

5 $$\begin{aligned} {\mathbb {P}}\,\left( {\hat{N}} = N, \max _{j=1,2,\ldots ,N}\left( \left| {\hat{r}}_j - r_j\right| \left( \varDelta _j^f\right) ^2\right) \le C_3\log T\right) \ge 1 - \frac{C_4}{T}. \end{aligned}$$

The isolation aspect of Isolate-Detect helps us to prove consistency under the conditions used in Theorem 1 (and later in Theorem 2). From (5), we notice that in order to be able to match the estimated change-point locations with the true ones, $\delta _T$ should be larger than $\underset{j=1,2,\ldots ,N}{\max }\left| {\hat{r}}_j - r_j\right|$, meaning that $\delta _T$ must be at least ${\mathcal {O}}(\log T)$. For this order of $\delta _T$, Chan and Walther (2013) argue that the smallest possible $\delta _T{\underline{f}}_T^2$ that allows change-point detection is ${\mathcal {O}}\left( \log T - \log (\log T)\right)$. In our case, assumption (A1) ensures that the ${\mathcal {O}}(\log T)$ rate for $\delta _T{\underline{f}}_T^2$ is attained, which is nearly optimal (up to the double logarithmic term). This provides evidence that ID allows for detection in complex scenarios, such as limited spacings between change-points. We mention that if $\delta _T$ is of higher order than ${\mathcal {O}}\left( \log T\right)$, then Assumption (A1) implies that ${\underline{f}}_T$ could decrease with *T*.

The quantity on the right-hand side of (5) is $1-{\mathcal {O}}(1/T)$; the same order as in WBS and NOT. However, ID gives a provably lower constant $C_4$ for the bound. To understand this consistency advantage of our method over, for example, NOT see our proof in Section 8 of the supplement and compare (17) with the result in Equation (19), p.28 in the online supplementary material of Baranowski et al. (2019). The rate of the lower bound for the threshold $\zeta _T$ is ${\mathcal {O}}\left( \sqrt{\log T}\right)$ and this is what will be used in practice as the default rate: we use

6 $$\begin{aligned} \zeta _T = C\sqrt{2\log T}. \end{aligned}$$

and the choice of the constant *C* will be explained in Sect. 4. Furthermore, (5) indicates that $\delta _T$ does not affect the rate of convergence of the estimated change-point locations; these only depend on $\varDelta _j^f$.

**Continuous, piecewise-linear signals.** Under Gaussianity and with $R_{s,e}^b({\varvec{X}})$ being the generalized log-likelihood ratio for all possible single change-points within [*s*, *e*), the idea is to find a contrast function $C_{s,e}^b({\varvec{X}})$, which is maximized at the same point as $R_{s,e}^b({\varvec{X}})$. The contrast function is constructed by taking inner products of the data with a contrast vector. In the case of continuous piecewise-linear signals, Baranowski et al. (2019) show that the contrast vector to be used is $\varvec{\phi _{s,e}^b} = \left( \phi _{s,e}^b(1),\ldots ,\phi _{s,e}^b(T)\right)$, where

7 $$\begin{aligned} \phi _{s,e}^b(t) = {\left\{ \begin{array}{ll} \alpha _{s,e}^b\beta _{s,e}^b\left[ (e+2b-3s+2)t - (be +bs - 2s^2+2s)\right] , &{} \quad t=s,\ldots ,b,\\ -\frac{\alpha _{s,e}^b}{\beta _{s,e}^b}\left[ (3e-2b-s+2)t - (2e^2+2e-be-bs)\right] , &{}\quad t=b+1,\ldots ,e,\\ 0, &{} \quad \text {otherwise}, \end{array}\right. } \end{aligned}$$

where $n=e-s+1$, $\alpha _{s,e}^b = (6/[n(n^2-1)(1+(e-b+1)(b-s+1)+(e-b)(b-s))])^{\frac{1}{2}}$ and $\beta _{s,e}^b = ([(e-b+1)(e-b)]/[(b-s+1)(b-s)])^{\frac{1}{2}}$. The contrast function is $C_{s,e}^b({\varvec{X}}) = \left| \left\langle {\varvec{X}},\varvec{\phi _{s,e}^b}\right\rangle \right|$. To explain the reasoning behind the choice of the triangular function $\phi _{s,e}^b(\cdot )$, we define, for the interval [*s*, *e*], the linear vector $\gamma _{s,e}(t) = \left( \frac{1}{12}(e-s+1)\left( e^2 - 2es +2e +s^2-2s\right) \right) ^{-\frac{1}{2}}\left( t-\frac{e+s}{2}\right)$, $t=s,\ldots ,e$ (and 0 otherwise) as well as the constant vector $1_{s,e}(t) = \left( e-s+1\right) ^{-\frac{1}{2}}$, $t=s,\ldots ,e$ (and 0 otherwise). On the vector ${\tilde{\phi }}_{s,e}^b(t) = t - b$, $t=b+1,\ldots ,e$ (and 0 otherwise), which is linear with a kink at $b+1$, we apply the Gram-Schmidt orthogonalization with respect to $\gamma _{s,e}(t)$ and $1_{s,e}(t)$. Normalizing the obtained vector such that $\Vert .\Vert _2 = 1$ returns the contrast vector $\phi _{s,e}^b(t)$ defined in (7). The best approximation, in terms of the Euclidean distance, of $X_t$ in [*s*, *e*] is a linear combination of $\gamma _{s,e}(t)$, $1_{s,e}(t)$, and $\phi _{s,e}(t)$, which are mutually orthonormal (Baranowski et al. 2019). This orthonormality leads to $R_{s,e}^b({\varvec{X}}) = \left| \left\langle {\varvec{X}},\varvec{\phi _{s,e}^b}\right\rangle \right| = C_{s,e}^b({\varvec{X}})$. For the consistency of ID in continuous piecewise-linear signals, we make the following assumption.

(A2) The minimum distance, $\delta _T$, between two change-points and the minimum magnitude of jumps, ${\underline{f}}_T = \min _{j=1,2,\ldots ,N}\left| 2f_{r_j} - f_{r_{j}+1} - f_{r_{j}-1}\right|$, are connected by the requirement $\delta _T^{3/2}{\underline{f}}_T \ge C^*\sqrt{\log T}$, for a large enough constant $C^*$.

The term $\delta _T^{3/2}{\underline{f}}_T$ characterizes the difficulty level of the detection problem and is analogous to $\sqrt{\delta _T}{\underline{f}}_T$ in the scenario of piecewise-constant signals. Theorem 2 gives the consistency result for the case of continuous piecewise-linear signals. The proof is in Section 8 of the supplement.

#### Theorem 2

Let $\left\{ X_t \right\} _{t=1,2,\ldots ,T}$ follow model (3) with $f_t$ being a continuous, piecewise-linear signal and assume that the random sequence $\left\{ \epsilon _t\right\} _{t=1,2,\ldots ,T}$ is independent and identically distributed (i.i.d.) from the normal distribution with mean zero and variance one and that (A2) holds. We denote by *N* and $r_j, j=1,2,\ldots ,N$ the number and locations of the change-points, while ${\hat{N}}$ and ${\hat{r}}_j, j=1,2,\ldots ,{\hat{N}}$ are their estimates sorted in increasing order. Also, we denote $\varDelta _j^f = \left| 2f_{r_j} - f_{r_{j}+1} - f_{r_{j}-1}\right|$. Then, there exist positive constants $C_1, C_2, C_3, C_4$, which do not depend on *T*, such that for $C_1\sqrt{\log T}\le \zeta _T < C_2\delta _T^{3/2}{\underline{f}}_T$ and for sufficiently large *T*,

8 $$\begin{aligned} {\mathbb {P}}\,\left( {\hat{N}} = N, \max _{j=1,2,\ldots ,N}\left( \left| {\hat{r}}_j - r_j\right| \left( \varDelta _j^f\right) ^{2/3}\right) \le C_3(\log T)^{1/3}\right) \ge 1 - \frac{C_4}{T}. \end{aligned}$$

The quantity on the right-hand side of (8) is $1-{\mathcal {O}}\left( 1/T\right)$. In addition, in the case of ${\underline{f}}_T \sim T^{-1}$, ID’s change-point detection accuracy is ${\mathcal {O}}\left( T^{2/3}\left( \log T\right) ^{1/3}\right)$, as can be seen from (8). This differs from the ${\mathcal {O}}\left( T^{2/3}\right)$ rate derived in Raimondo (1998) only by the logarithmic factor. The lower bound of the threshold is ${\mathcal {O}}\left( \sqrt{\log T}\right)$. Therefore,

9 $$\begin{aligned} \zeta _T = {\tilde{C}}\sqrt{2\log T}, \end{aligned}$$

where ${\tilde{C}}$ is a constant and we will comment on its choice in Sect. 4.2.

ID is flexible because it does not depend on the structure of the signal; what changes is the choice of an appropriate contrast function. Adopting a similar approach as the one for the case of continuous piecewise-linear signals, one can construct contrast functions for the detection of other types of features.

### Information criterion approach

Misspecification of the threshold in the ID algorithm can lead to the misestimation of the number of change-points. To remedy this, we develop an approach which starts by possibly overestimating the number of change-points and then creates a solution path, with the estimates ordered according to a certain predefined criterion. The best fit is then chosen, based on the optimization of a model selection criterion.

**The solution path algorithm:** The estimated number of change-points depends on $\zeta _T$ and this allows us to denote ${\hat{N}} = {\hat{N}}(\zeta _T)$. For given data, we employ ID using first $\zeta _T$ and then ${\tilde{\zeta }}_T$, where ${\tilde{\zeta }}_T < \zeta _T$. Let $C_{{\tilde{\zeta }}_T}$ and ${\tilde{C}}_{{\tilde{\zeta }}_T}$ be the ${\tilde{\zeta }}_T$-associated constants in (6) and (9), respectively. With $J \ge {\hat{N}}(\zeta _T)$, we estimate ${\tilde{r}}_j, j=1,2,\ldots , J$, which are sorted in increasing order in ${\tilde{S}} = \left[ {\tilde{r}}_1, {\tilde{r}}_2, \ldots , {\tilde{r}}_{J}\right]$. Our aim is to prune the estimates through an iterative procedure, where at each iteration the estimation most likely to be spurious is removed. The algorithm is split into four parts, with their descriptions being fairly technical. We note however that the different parts are very similar and are based on the idea of removing change-points according to their contrast function values as well as their distance to neighbouring estimates. Even though the full explanation of each part is in Section 1 of the supplement, we now provide a brief summary for the framework of the solution path algorithm. With ${\tilde{r}}_0 = 1$ and ${\tilde{r}}_{J+1} = T$, we first collect triplets $({\tilde{r}}_{j-1},{\tilde{r}}_j,{\tilde{r}}_{j+1})$, $\forall \left\{ 1,2,\ldots , J\right\}$ and we calculate $\mathrm{CS}({\tilde{r}}_j) := C_{{\tilde{r}}_{j-1},{\tilde{r}}_{j+1}}^{{\tilde{r}}_j}({\varvec{X}}),$ with $C_{s,e}^b({\varvec{X}})$ being the relevant contrast function. For $m = \mathrm{argmin}_{j}\left\{ \mathrm{CS}({\tilde{r}}_j) \right\}$ we check whether $CS({\tilde{r}}_m) \le \tilde{{\tilde{C}}}\sqrt{\log T}$, for $\tilde{{\tilde{C}}} > 0$; in the proofs of Theorems 3 and 4, $\tilde{{\tilde{C}}} = 2\sqrt{2}$ but smaller values could be sufficient; see for example Corollary 1. If $CS({\tilde{r}}_m) \le \tilde{{\tilde{C}}}\sqrt{\log T}$, we remove ${\tilde{r}}_m$ from ${\tilde{S}}$, reduce *J* by 1, relabel the remaining estimates (in increasing order) in ${\tilde{S}}$, and repeat this estimate removal process, which is carried out in a way such that once the set ${\tilde{S}}$ contains *N* estimates, then for $j=1,2,\ldots ,N$, each ${\tilde{r}}_j$ is within a distance of $C^*\left( \log T\right) ^{\alpha }$ from the true change-point $r_j$. We keep removing estimates until ${\tilde{S}} = \emptyset$.

At the end of this change-point removal approach, we collect the estimates in

10 $$\begin{aligned} {\varvec{b}} = \left( b_1,b_2,\ldots ,b_J\right) , \end{aligned}$$

where $b_J$ is the estimate that was removed first, $b_{J-1}$ is the one that was removed second, and so on. From now on, the vector ${\varvec{b}}$ is called the solution path and is used to give a range of different fits. We define the collection $\left\{ {\mathcal {M}}_j\right\} _{j = 0,1,\ldots ,J}$ where ${\mathcal {M}}_{0} = \emptyset$ and ${\mathcal {M}}_j = \left\{ b_1,b_2,\ldots ,b_j\right\}$. For $j = 2,\ldots ,J$, let ${\tilde{b}}_1< \ldots < {\tilde{b}}_j$ be the sorted elements of ${\mathcal {M}}_j$. Among the collection of models $\left\{ {\mathcal {M}}_j\right\} _{j=0,1,\ldots ,J}$, we propose to select the one that minimizes the strengthened Schwarz Information Criterion (Liu et al. 1997; Fryzlewicz 2014), defined as

11 $$\begin{aligned} \mathrm{sSIC}(j) = -2\sum _{k=1}^{j+1}\ell \left( X_{{\tilde{b}}_{k-1} + 1},\ldots ,X_{{\tilde{b}}_k};{\hat{\theta }}_k\right) + n_{j}\left( \log T\right) ^{\alpha }, \end{aligned}$$

where ${\tilde{b}}_0 = 0$ and for each collection ${\mathcal {M}}_j$, ${\tilde{b}}_{j+1} = T$ and ${\hat{\theta }}_1,{\hat{\theta }}_2,\ldots ,{\hat{\theta }}_{j+1}$ are the maximum likelihood estimators of the segment parameters for the model (3) with change-point locations $b_1,b_2,\ldots ,b_j$. The quantity $n_j$ is the total number of estimated parameters related to ${\mathcal {M}}_j$. For example, if we do not consider the change-point locations as free parameters, then in the scenario of piecewise-constant mean $n_j = j+1$ (the constant values for each of the $j+1$ segments), while in the scenario of continuous and piecewise-linear signals $n_j = j+2$ (the starting intercept and slope and the *j* changes in the slope). We mention that if the continuity constraint is to be removed, then $n_j$ would be equal to $2j+2$ (the constant and slope values for the $j+1$ segments). If now we consider the change-point locations to be free parameters, then we just need to add *j* in the above values for $n_j$ in the different scenarios.

In the algorithm we have referred to three parameters: $C^*, \tilde{{\tilde{C}}}$, and $\alpha$. Although we do not give a recipe for the choice of $C^*$ and $\tilde{{\tilde{C}}}$, Sect. 3 describes how to circumvent their choice. With respect to $\alpha$, taking its value to be equal to 1 in (11) gives the standard SIC penalty, but our theory requires $\alpha > 1$. In practice we use $\alpha = 1.01$ in order to remain close to SIC. Theorems 3 and 4 below give the consistency results for the piecewise-constant and continuous piecewise-linear models, based on the sSIC approach. The proof of Theorem 3 is in the supplementary material and the same approach can be followed to prove Theorem 4.

#### Theorem 3

Let $\left\{ X_t \right\} _{t=1,2,\ldots ,T}$ follow model (3) under piecewise-constancy and let the assumptions of Theorem 1 hold. Let *N* and $r_j, j=1,2,\ldots ,N$ be the number and locations of the change-points. Let $N \le J$, where *J* can also grow with *T*. In addition, let $\alpha > 1$ be such that $\left( \log T\right) ^\alpha = o(\delta _T{\underline{f}}_T^2)$ is satisfied, where $\delta _T$ and ${\underline{f}}_T$ are defined in (A1). With $\left\{ {\mathcal {M}}_{j}\right\} _{j=0,1,\ldots ,J}$ being the set of candidate models obtained by the solution path algorithm, we define ${\hat{N}} = \mathrm{argmin}_{j=0,1,\ldots ,J}\;\mathrm{sSIC}(j)$. Then, there exist positive constants $C_1, C_2$, which do not depend on *T*, such that for $\varDelta _j^f = \left| f_{r_j+1} - f_{r_j}\right|$,

12 $$\begin{aligned} {\mathbb {P}}\,\left( {\hat{N}} = N, \max _{j=1,2,\ldots ,N}\left( \left| {\hat{r}}_j - r_j\right| \left( \varDelta _j^f\right) ^2\right) \le C_1\left( \log T\right) ^{\alpha }\right) \ge 1 - \frac{C_2}{T}. \end{aligned}$$

#### Theorem 4

Let $\left\{ X_t \right\} _{t=1,2,\ldots ,T}$ follow model (3) under continuous piecewise-linearity and let the assumptions of Theorem 2 hold. Let *N* and $r_j, j=1,2,\ldots ,N$ be the number and locations of the change-points. Let $N \le J$, where *J* can also grow with *T*. In addition, let $\alpha > 1$ be such that $\left( \log T\right) ^\alpha = o(\delta _T^3{\underline{f}}_T^2)$ is satisfied, where $\delta _T$ and ${\underline{f}}_T$ are defined in (A2). With $\left\{ {\mathcal {M}}_{j}\right\} _{j=0,1,\ldots ,J}$ being the set of candidate models obtained by the solution path algorithm, we define ${\hat{N}} = \mathrm{argmin}_{j=0,1,\ldots ,J}\;\mathrm{sSIC}(j)$. Then, there exist positive constants $C_1, C_2$, which do not depend on *T*, such that for $\varDelta _j^f = \left| 2f_{r_j} - f_{r_{j}+1} - f_{r_{j}-1}\right|$,

13 $$\begin{aligned} {\mathbb {P}}\,\left( {\hat{N}} = N, \max _{j=1,2,\ldots ,N}\left( \left| {\hat{r}}_j - r_j\right| \left( \varDelta _j^f\right) ^{2/3}\right) \le C_1(\log T)^{\alpha /3}\right) \ge 1 - \frac{C_2}{T}. \end{aligned}$$

We note that our solution path algorithm, explained in detail in Section 1 of the supplementary material, allows *J*, the number of the detections from the already explained overestimation process, to grow with *T*. The quantities on the right hand sides of (12) and (13) are $1-{\mathcal {O}}\left( 1/T\right)$; the same order as those in (5) and (8). The lowest admissible $\delta _T{\underline{f}}_T^2$ and $\delta _T^3{\underline{f}}_T^2$ in Theorems 3 and 4, respectively, are slightly larger than the same quantities in the thresholding approach. Our empirical expertise suggests that SIC-based approaches tend to exhibit better practical behaviour for signals that have a moderate number of change-points and/or large spacings between them. A hybrid that combines the advantages of the thresholding and the SIC-based approach is introduced in Sect. 4.4.

## Computational complexity and practicalities

### Computational cost

With $\delta _T$ being the minimum distance between two change-points, and $\lambda _T$ the interval-expansion parameter, we use $\lambda _T < \delta _T$. We note that while $\delta _T$ is unknown, choosing $\lambda _T$ small enough guarantees with high probability that this requirement holds; see Sect. 4.2 for how to choose $\lambda _T$ in order to obtain good accuracy performance and at the same time low computational cost. Now, since $K = \lceil T/\lambda _T\rceil > \lceil T/\delta _T \rceil$ and the total number, $M_{ID}$, of intervals required to scan the data is no more than 2*K* (*K* intervals from each expanding direction), in the worst case scenario we have $M_{ID} = 2K > 2\left\lceil \frac{T}{\delta _T}\right\rceil$. As a comparison, in WBS and NOT one needs to draw at least *M* intervals where $M \ge \left( 9T^2/\delta _T^2\right) \log \left( T^2/\delta _T\right)$. The lower bound for *M* in WBS and NOT is ${\mathcal {O}}\left( T^2/\delta _T^2\right)$ up to a logarithmic factor, whereas the lower bound for $M_{ID}$ is ${\mathcal {O}}\left( T/\delta _T\right)$. This results in great speed gains of ID over WBS and NOT. The reason behind this significant difference in the computational complexity of the methods is that in WBS and NOT both the start- and end-points of the randomly drawn intervals have to be chosen, whereas in ID, depending on the expanding direction, we keep the start- or the end-point fixed.

### Parameter choice

**Choice of the threshold constant.** We start with an upper bound on the constant *C*, as defined in (6), for the case of piecewise-constant signals when the error terms $\epsilon _t$ are i.i.d. from the Gaussian distribution. We note that this result is of independent interest. Our model is as in (1) for stationary $\epsilon _t$. For any vector ${\varvec{y}} \in {\mathbb {R}}^{T}$, we define

14 $$\begin{aligned} {\tilde{y}}_{s,e}^b = \sqrt{\frac{e-b}{n(b-s+1)}}\sum _{t=s}^{b}y_t - \sqrt{\frac{b-s+1}{n(e-b)}}\sum _{t=b+1}^{e}y_t;\quad \tilde{{\tilde{y}}}_{s,e} = \frac{\sum _{t=s}^e y_t}{(e-s+1)^{1/2}},\nonumber \\ \end{aligned}$$

where $1 \le s \le b < e \le T$ and $n = e-s+1$. It can be shown that if $\epsilon _t$, are serially independent and their distribution is symmetric about zero (for example i.i.d. standard Gaussian random variables), then the sequence $\left\{ \epsilon _t\right\} _{t=1}^T$ satisfies

15 $$\begin{aligned} \forall \,\gamma>0,\quad P\left( \min _{s,b,e}\,\, \tilde{{\tilde{\epsilon }}}_{s,b} \tilde{{\tilde{\epsilon }}}_{b+1,e} < -\gamma \right) \le P\left( \max _{s,b,e}\,\, \tilde{{\tilde{\epsilon }}}_{s,b} \tilde{{\tilde{\epsilon }}}_{b+1,e} > \gamma \right) \end{aligned}$$

The following corollary indicates that as $T\rightarrow \infty$, we have that $C \le \sqrt{3/2}$, meaning that the threshold can be taken to be at most $\sqrt{3\log T}$. This value of $\sqrt{3}$ is smaller than the constant used in the solution path algorithm of Sect. 3.3 ($\tilde{{\tilde{C}}} = 2\sqrt{2}$), which can however be used to give explicit upper bounds on the consistency results as explained in Theorems 1 and 3; in contrast, Corollary 1 does not give an explicit upper bound for the probability related to the consistency result as expressed in (16). We highlight that the aforementioned bound on the constant and its proof are simpler than the results presented in Fang et al. (2020) which involve the manipulation of complex distributions. The proof is in the supplementary material.

#### Corollary 1

Let $\{\epsilon _t\}_{t=1}^T$ be i.i.d. $N(0, \sigma ^2)$. For any $\delta > 0$,

16 $$\begin{aligned} {\mathbb {P}}\,\left( \exists _{s,a,e}\,\, \left( {\tilde{\epsilon }}_{s,e}^b\right) ^2 > 3 \sigma ^2 (1 + \delta ) \log \,T \right) \xrightarrow [T \rightarrow \infty ]{} 0. \end{aligned}$$

For the practical choice of the values of *C* and ${\tilde{C}}$, in (6) and (9), respectively, we ran a large-scale simulation study involving a wide range of signals. The number of change-points, *N*, was generated from the Poisson distribution with rate parameter $N_{\alpha }\in \left\{ 4,8,12 \right\}$. For $T \in \left\{ 100,200,500,1000,2000,5000 \right\}$, we uniformly distributed the change-points in $\left\{ 1,2,\ldots ,T \right\}$. Then, for piecewise-constant (or continuous piecewise-linear) signals, at each change-point location we introduced a jump (or a slope change) which followed the normal distribution with mean zero and variance $\sigma ^2 \in \left\{ 1,3,5 \right\}$. Standard Gaussian noise was then added onto the simulated signal. For each value of $N_\alpha$, $\sigma ^2$ and *T* we generated 1000 replicates and estimated the number of change-points using ID with threshold $\zeta _T$ as in (6) and (9) for a variety of constant values *C* and ${\tilde{C}}$. The best behaviour occurred when, approximately, $C=1.05$ and ${\tilde{C}} = 1.4$. These values will be referred to as the default constants and they hold true for all signals that satisfy the assumption of the error terms $\epsilon _t$ being i.i.d. Gaussian. We note that the value of ${\tilde{C}} = 1.4$ does not violate Corollary 1 because the result expressed in the latter is only for piecewise-constant signals, while the constant ${\tilde{C}}$ applies to the scenario of continuous, piecewise-linear signals. Due to the fact that the contrast function used is based on local averaging, the CLT can be used to show that for sufficiently large sample size *T*, ID is robust when the normality assumption is not satisfied; this has also been explored in Fearnhead and Rigaill (2020). Also, pre-averaging is a practical approach that we employed in Sect. 4.5 for such cases with error departures from Gaussianity.

In the SIC-based approach of Sect. 3.3, we started by detecting change-points using threshold ${\tilde{\zeta }}_T < \zeta _T$. In practice, we take the constants related to ${\tilde{\zeta }}_T$, namely $C_{{\tilde{\zeta }}_T}$ and ${\tilde{C}}_{{\tilde{\zeta }}_T}$ as defined in Sect. 3.3, to be 0.9 and 1.25, respectively.

**Choice of the expansion parameter** $\lambda _T$. We start by highlighting that our numerical experience suggests that ID is robust to small changes in the value of $\lambda _T$; for a small-scale simulation study when the value of $\lambda _T$ changes significantly ($\lambda _T \in \left\{ 5,20,80\right\}$), see Section 6 of the supplementary material. Theoretically, for a given signal, the change-point detection results obtained from ID are the same for any value of $\lambda _T$ used which is less than the minimum spacing between two successive change-points. The computational cost of running ID is inversely proportional to the size of the expansion parameter; the smaller the $\lambda _T$, the more intervals we need to work on. However, the low computational complexity of our algorithm allows us to take $\lambda _T$ to be as small as the value of three leading to very good accuracy even for signals with frequent change-points. We now give example execution times for two models, (T1) and (T2) defined below, on a 3.60GHz CPU with 16 GB of RAM. We employed the ID-variant for long signals explained in Sect. 3.

1. Length $l_j = 7\times 10^{j}, j=3,4,5$, with change-points at $7, 14, \ldots , l_j - 7$ and values between them $0,4,0,4,\ldots , 0, 4$. The standard deviation is $\sigma =0.5$. Execution times: 0.31s ($j=3$), 2.25s ($j=4$), 26.41s ($j=5$).
2. Length $l_j = 7 \times 10^{j}, j=3,4,5$, with no change-points. We use $\sigma =1$. Execution times: 0.64s ($j=3$), 3.01s ($j=4$), 30.35s ($j=5$).

### Variants

Here, we describe three different ways to further improve ID’s practical performance.

*Long signals:* If *T* is large, we split the given data sequence uniformly into smaller parts (windows), to which ID is then applied. In practical implementations, the length of the window is 3000 and we apply this structure only when $T > 12000$, because for smaller values of *T* there are no significant differences in the execution times of ID and its window-based variant. The computational improvement that this structure offers is explained in Section 3 of the supplement.

*Restarting after detection:* In practice, instead of starting from the end-point $e^*$ (or start-point $s^*$) of the right-expanding (or left-expanding) interval where a detection occurred, we could start from the estimated change-point, ${\hat{b}}$. This alternative, labelled $\mathrm{ID}_{det}$, leads to accuracy improvement without affecting the speed of the method.

*Faster solution path algorithm:* In practice, we use only Part 4 of the solution path algorithm described in Section 1 of the supplement because it is quicker and conceptually simpler; it requires only the choice of $\alpha$ and tends not to affect ID’s accuracy.

### Alternative model selection criteria

*A hybrid between thresholding and SIC stopping rules:* For signals with a large number of regularly occurring change-points, the threshold-based ID tends to behave better than the SIC-based procedure. As explained after Theorems 3 and 4, this is unsurprising because SIC-based approaches typically perform better on signals with a moderate number of change-points separated by larger spacings. This difference in ID’s behaviour between the threshold- and SIC-based versions is what motivates us to introduce a hybrid of these two stopping rules with minimal parameter choice, which works as follows. Firstly, we estimate the change-points using the threshold approach $\mathrm{ID}_{det}$ with $\lambda _T^{th} = 3$. If the estimated change-points are more than a constant $J^*$, then the result is accepted and we stop. Otherwise, the hybrid method proceeds to detect the change-points using the SIC-based approach with $\lambda _T > \lambda _T^{th}$, since the already-applied thresholding rule has not suggested a signal with many change-points. In the simulations, we use $J^* = 100$, $\lambda _T=10$.

*Steepest Drop to Low Levels (SDLL):* We also combine ID with the SDLL model selection method introduced in Fryzlewicz (2020).

### Extension to different noise structures

This section describes how to use ID when the noise is not Gaussian. We pre-process the data in order to obtain a noise structure that is closer to Gaussianity. For a given scale number *s* and data $\left\{ X_t \right\} _{t=1,2,\ldots ,T}$, let $Q= \lceil T/s \rceil$ and ${\tilde{X}}_q = \frac{1}{s}\sum _{t=(q-1)s+1}^{qs}X_t$, for $q=1,2,\ldots ,Q-1$, while ${\tilde{X}}_Q = (T - (Q-1)s)^{-1}\sum _{t=(Q-1)s+1}^{T}X_t$. We apply ID on $\left\{ {\tilde{X}}_q\right\} _{q=1,2,\ldots ,Q}$ to obtain the estimated change-points, namely $\tilde{{\tilde{r}}}_1,\tilde{{\tilde{r}}}_2,\ldots ,\tilde{{\tilde{r}}}_{{\hat{N}}}$, in increasing order. To estimate the original locations of the change-points we define ${\hat{r}}_k = \left( \tilde{{\tilde{r}}}_k-1\right) s + \left\lfloor \frac{s}{2} + 0.5 \right\rfloor , \; k=1,2,\ldots ,{\hat{N}}$. The larger the value of *s*, the closer the distribution of the noise to normal, but the more the amount of pre-processing. In simulations presented in Sect. 5, we use $s=3$ for the case of Student-$t_5$ distributed noise, while if the tails are heavier (Student-$t_3$), we set $s=5$. The hybrid version of ID will be employed on $\left\{ {\tilde{X}}_q\right\} _{q=1,2,\ldots ,Q}$ and in order to be consistent with the choice of the expansion parameter, we take $\lambda ^*_T = \left\lfloor \lambda _T/s\right\rfloor$. In practice, for unknown noise, our recommendation is to set $s=5$.

## Simulations

This section compares the performance of ID with competitors. The main change-point detection ${\mathsf {R}}$ functions in the competing packages were called using their default input arguments, which does not always allow direct like-for-like comparisons of the methods. Whenever needed (difficult signal structures), and in order to help the competitors capture their best possible performance, the input values were adjusted accordingly. The ${\mathsf {R}}$ code used for the simulation study is available from Github at https://github.com/Anastasiou-Andreas/IDetect/blob/master/R/Simulations_used.R. Table 2 shows the competitors used. CPOP is employed based on ${\mathsf {R}}$ code found in http://www.research.lancs.ac.uk/portal/en/datasets/cpop(56c07868-3fe9-4016-ad99-54439ec03b6c).html and TF in https://stanford.edu/~boyd/l1_tf. For WBS, we give results based on both the information criterion and the thresholding (for $C=1$) stopping rules. The notation is WBSIC and WBSC1, respectively. With respect to WBS2, its performance is investigated based on the SDLL model selection criterion introduced in Fryzlewicz (2020). In the **cpm** package, the threshold is decided through the average run length (ARL) until a false positive occurs. In our simulations, we give results for $\mathrm{ARL}=500$ (the default value) and if the signal length, $l_s$, is greater than 500, results are also given for $\mathrm{ARL} = 1000\lceil ls/1000 \rceil$. The notation is CPM.*l*.*A*, with *A* the value of ARL. For FKS, when the number of knots is unknown (the scenario we work in), we need to specify the maximum allowed number of knots. We take this to be 2*N*, with *N* the true number of change-points. Also, the estimated change-points by FKS are positive real numbers; we take as estimation the closest integer. The proposed ID version is the hybrid described in Sect. 4.4. However, we also present the results for two more variants: SDLL and thresholding with constant $\sqrt{3/2}$ (see (6)), which is the upper bound proven in Corollary 1. The notation for these variants is ID.SDLL and $\hbox {ID}_{\sqrt{3/2}}$, respectively.

**Table 2:** The competing methods used in the simulation study

Type of signal	Method notation	Reference	R package
Piecewise-constant	PELT	Killick et al. (2012)	changepoint
	NP.PELT	Haynes et al. (2017)	changepoint.np
	S3IB	Rigaill (2015)	Segmentor3IsBack
	CumSeg	Muggeo and Adelfio (2011)	cumSeg
	CPM	Ross (2015)	cpm
	WBS	Fryzlewicz (2014)	wbs
	WBS2	Fryzlewicz (2020)	breakfast
	NOT	Baranowski et al. (2019)	not
	FDR	Li et al. (2016)	FDRSeg
	TGUH	Fryzlewicz (2018)	breakfast
Continuous piecewise-linear	NOT	Baranowski et al. (2019)	not
	TF	Kim et al. (2009)	–
	CPOP	Maidstone et al. (2019)	–
	MARS	Friedman (1991)	earth
	FKS	Spiriti et al. (2013)	freeknotsplines

**Fig. 4:**
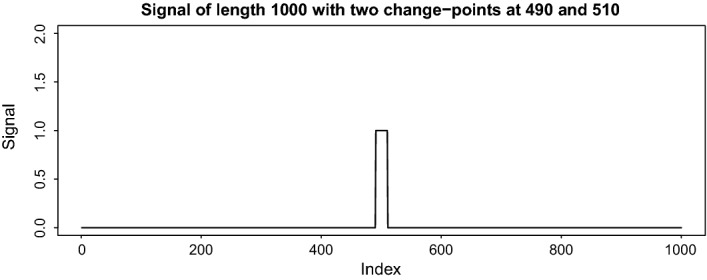
Example of a signal of length 1000 with change-points at 490 and 510 offsetting each other

**Fig. 5:**
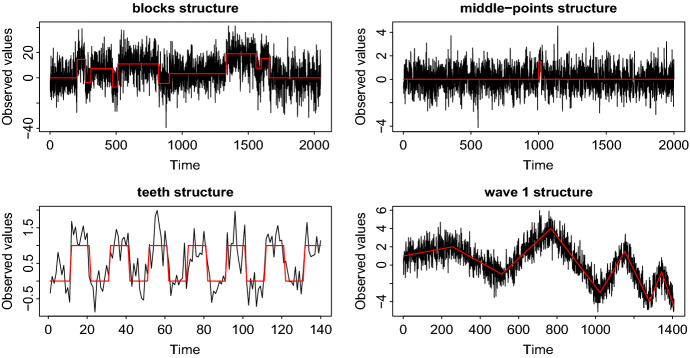
Examples of data series, used in simulations. The true signal, $f_t$, is in red

**A seemingly difficult structure for ID:** Signals that present the most difficulty to ID are ones in which change-points are concentrated in the middle part of the data and offset each other, as in Fig. 4. The reason is that due to the left- and right- expanding feature of ID, where one of the two end-points of the interval is kept fixed, the change-points need to be detectable based on relatively “unbalanced” (explanation follows directly below) tests, which typically tend to offer poor power. For example, referring again to Fig. 4, the change-point at 490 will need to be isolated and detected by comparing the means of the data over the long interval [1, 490] and a short interval of the form $[491,e_j]$, where $e_j \le 510$ is the end-point of a right-expanding interval $[1,e_j]$. To be more precise, if the expansion parameter $\lambda = 3$, then $e_j \in \left\{ 492, 495, \ldots , 510\right\}$ and therefore our procedure will have seven opportunities to detect the change-point 490 while it is still isolated in intervals that do not contain any other change-points. Even though ID would be expected to struggle in detecting the change-points in such unbalanced intervals, our numerical experience suggests that its performance on such challenging signals is in fact very good and matches or surpasses that of the best competitors; see for example the results in Table 3 for the model (M4), which follows this structure. All the signals are fully specified in Section 2 of the supplementary material. Figure 5 shows examples of the data generated by models (M1) *blocks*, (M2) *teeth*, (M4) *middle-points*, and (W1) *wave 1*. Tables 3, 4, 5, 6 and 7 summarize the results in the case of i.i.d. Gaussian noise. Table 8 presents the behaviour of ID under the setting of i.i.d. scaled Student-$t_d$ noise, where $d=3,5$. More examples are in the supplement.

We highlight that the NOT, WBSIC, and S3IB methods require the specification of the maximum number, $K_{max}$, of change-points allowed to be detected. If the default values in these methods are lower than the true number of change-points in the simulated examples, then we take $K_{max} = \lceil T/\delta _T\rceil$, where $\delta _T$ is the minimum distance between two change-points. We ran 100 replications for each signal and the frequency distribution of ${\hat{N}} - N$ for each method is presented. The methods with the highest empirical frequency of ${\hat{N}} - N = 0$ (or in a neighbourhood of zero, depending on the example) and those within $10\%$ off the highest are given in bold. As a measure of the accuracy of the detected locations, we provide Monte-Carlo estimates of the mean squared error, $\mathrm{MSE} = T^{-1}\sum _{t=1}^{T}{{\mathbb {E}}} \left( {\hat{f}}_t - f_t\right) ^2$, where ${\hat{f}}_t$ is the ordinary least square approximation of $f_t$ between two successive change-points. In continuous piecewise-linear signals, ${\hat{f}}_t$ is the splines fit obtained using the **splines** package in R. The scaled Hausdorff distance, $d_H = n_s^{-1}\max \left\{ \max _j\min _k\left| r_j-{\hat{r}}_k\right| ,\max _k\min _j\left| r_j-{\hat{r}}_k\right| \right\} ,$ where $n_s$ is the length of the largest segment, is also given in all examples apart from the signal (NC) in Table 5, which is a constant-mean signal with no change-points.

The average computational time for all methods, apart from FDR, is also provided. FDR is excluded due to its non-uniform procedure in terms of the execution speed for each signal (if a newly obtained signal has length greater than previously treated signals, then FDR estimates the threshold by 5000 Monte-Carlo simulations, which makes it slow). In some cases the average computational time for FKS is not given. We have already explained that we need to pre-specify the maximum allowed number of knots in order for FKS to work. The method is somewhat slow and we exclude the results for FKS when the true change-points are more than 10, as in such cases it would take a significant amount of time to finish all the 100 simulations.

**Table 3:** Distribution of ${\hat{N}} - N$ over 100 simulated data sequences of the piecewise-constant signals (M1)–(M4)

Method	Model								MSE	$d_H$	Time (ms)
		$${\hat{N}} - N$$									
		$\le -3$	$-2$	$-1$	0	1	2	$\ge 3$			
PELT		6	32	50	12	0	0	0	3.23	0.14	3
NP.PELT		0	2	27	49	15	5	2	2.82	0.10	211.8
S3IB		0	7	38	54	1	0	0	2.49	0.08	343.2
CumSeg		39	21	38	2	0	0	0	6.37	0.20	62.3
CPM.*l*.500		0	0	0	3	3	4	90	4.45	0.44	2.3
CPM.*l*.3000		0	0	8	41	26	19	6	3.03	0.19	3.3
WBSC1	(M1)	0	0	11	32	27	19	11	2.79	0.25	99.3
WBSIC		0	3	37	53	7	0	0	2.59	0.08	99.3
WBS2		0	3	54	31	8	2	2	2.64	0.09	623.3
NOT		0	3	51	43	3	0	0	2.61	0.10	80.7
FDR		0	0	33	54	12	1	0	2.51	0.09	–
TGUH		0	5	37	49	7	1	1	3.30	0.08	127.4
**ID**		0	3	30	**62**	5	0	0	2.66	0.08	23.9
ID.SDLL		1	2	59	28	5	3	2	2.80	0.10	20
$\hbox {ID}_{\sqrt{3/2}}$		0	9	62	28	1	0	0	2.75	0.09	22.3
PELT		85	6	0	9	0	0	0	181 $\times 10^{-3}$	6.62	1.1
NP.PELT		84	12	3	1	0	0	0	165 $\times 10^{-3}$	4.26	3.1
S3IB		41	15	1	43	0	0	0	117 $\times 10^{-3}$	3.73	15.2
CumSeg		100	0	0	0	0	0	0	251 $\times 10^{-3}$	–	3.9
CPM.*l*.500		78	4	15	3	0	0	0	145 $\times 10^{-3}$	2.96	0.4
**WBSC1**	(M2)	1	2	7	**72**	12	6	0	53 $\times 10^{-3}$	0.33	38.2
**WBSIC**		7	8	1	**68**	13	3	0	64 $\times 10^{-3}$	1.00	38.2
**WBS2**		3	3	4	**71**	10	4	5	58 $\times 10^{-3}$	0.363	30.5
**NOT**		9	7	4	**73**	6	1	0	65 $\times 10^{-3}$	0.97	43.4
FDR		14	11	11	55	7	2	0	71 $\times 10^{-3}$	0.80	–
**TGUH**		4	18	3	**68**	7	0	0	64 $\times 10^{-3}$	0.47	22.8
**ID**		7	7	1	**74**	11	0	0	60 $\times 10^{-3}$	0.87	8.8
ID.SDLL		5	5	6	63	8	4	9	62 $\times 10^{-3}$	0.43	3.7
$\hbox {ID}_{\sqrt{3/2}}$		28	13	9	47	3	0	0	84 $\times 10^{-3}$	0.90	5.3
**PELT**		0	2	7	**90**	1	0	0	23 $\times 10^{-3}$	0.15	1.1
NP.PELT		100	0	0	0	0	0	0	781 $\times 10^{-3}$	1.78	4.2
S3IB		98	1	1	0	0	0	0	213 $\times 10^{-3}$	0.91	20.2
CumSeg		0	3	16	72	9	0	0	65 $\times 10^{-3}$	0.32	5.2
CPM.*l*.500		1	6	87	6	0	0	0	51 $\times 10^{-3}$	0.85	0.2
WBSC1	(M3)	0	0	0	66	26	7	1	24 $\times 10^{-3}$	0.19	37.3
WBSIC		0	0	0	64	27	9	0	24 $\times 10^{-3}$	0.18	37.3
**WBS2**		0	0	1	**87**	8	2	2	25 $\times 10^{-3}$	0.17	34.7
**NOT**		0	0	0	**93**	7	0	0	21 $\times 10^{-3}$	0.13	118.3
FDR		0	0	2	77	15	5	1	23 $\times 10^{-3}$	0.17	–
**TGUH**		0	0	1	**91**	6	2	0	25 $\times 10^{-3}$	0.15	25.2
**ID**		0	0	0	**91**	8	1	0	22 $\times 10^{-3}$	0.13	9.8
**ID.SDLL**		0	0	1	**97**	1	0	1	24 $\times 10^{-3}$	0.14	6.8
$\mathbf{ID }_{\sqrt{\mathbf{3/2 }}}$		0	0	2	**94**	3	1	0	23 $\times 10^{-3}$	0.15	4.4
PELT		–	53	0	47	0	0	0	14 $\times 10^{-3}$	0.54	6.7
NP.PELT		–	0	0	21	3	34	42	14 $\times 10^{-3}$	0.47	395.2
**S3IB**		–	12	0	**87**	1	0	0	7 $\times 10^{-3}$	0.12	292.1
CumSeg		–	100	0	0	0	0	0	23 $\times 10^{-3}$	–	84.6
CPM.*l*.500		–	0	0	0	0	6	94	31 $\times 10^{-3}$	0.76	14
CPM.*l*.2000		–	0	0	35	11	22	32	13 $\times 10^{-3}$	0.39	20.4
WBSC1	(M4)	–	0	0	23	20	17	40	13 $\times 10^{-3}$	0.50	120.8
**WBSIC**		–	4	0	**96**	1	0	0	5 $\times 10^{-3}$	0.04	119.2
WBS2		–	0	1	83	10	4	2	5 $\times 10^{-3}$	0.09	666.4
**NOT**		–	8	0	**92**	0	0	0	6 $\times 10^{-3}$	0.08	61.8
FDR		–	0	19	70	10	1	0	9 $\times 10^{-3}$	0.07	–
TGUH		–	0	51	40	7	2	0	23 $\times 10^{-3}$	0.28	169.2
**ID**		–	7	0	**93**	0	0	0	6 $\times 10^{-3}$	0.07	42.3
ID.SDLL		–	0	0	81	4	10	5	7 $\times 10^{-3}$	0.10	28.7
$\mathbf{ID }_{\sqrt{\mathbf{3/2 }}}$		–	1	0	**98**	1	0	0	5 $\times 10^{-3}$	0.05	66.4

The average MSE, $d_H$ and computational time are also given

**Table 4:** Distribution of ${\hat{N}} - N$ over 100 simulated data sequences from the piecewise-constant signal (M5)

Method						MSE	$d_H$	Time (s)
	$${\hat{N}} - N$$							
	$\le -500$	$(-500,-50]$	$(-50,-10)$	$[-10,10]$	$> 10$			
PELT	100	0	0	0	0	1.97	114.92	0.033
NP.PELT	100	0	0	0	0	2.25	551.89	8.976
S3IB	99	1	0	0	0	2.23	1979.95	332.841
CumSeg	100	0	0	0	0	2.25	1999	0.551
CPM.*l*.500	0	45	54	1	0	0.19	9.00	0.002
CPM.*l*.20000	100	0	0	0	0	2.23	1999	1.245
WBSC1	100	0	0	0	0	1.51	35.26	12.272
WBSIC	100	0	0	0	0	2.25	1999	12.272
WBS2	0	0	0	**100**	0	0.14	0.54	5.796
NOT	100	0	0	0	0	2.25	1999	0.484
FDR	0	0	0	5	95	0.14	0.51	–
**TGUH**	0	0	0	**100**	0	0.16	0.84	0.794
**ID**	0	0	0	**100**	0	0.14	0.99	0.785
**ID.SDLL**	0	0	0	**100**	0	0.14	0.71	120.601
$\hbox {ID}_{\sqrt{3/2}}$	0	82	18	0	0	0.22	2.48	1.363

The average MSE, $d_H$ and computational time are also given

**Table 5:** Distribution of ${\hat{N}} - N$ over 100 simulated data sequences from (NC)

Method					MSE	Time (s)
	$${\hat{N}} - N$$					
	0	1	2	$\ge 3$		
**PELT**	**100**	0	0	0	39 $\times 10^{-5}$	0.004
NP.PELT	8	1	23	68	999 $\times 10^{-5}$	1.077
**S3IB**	**100**	0	0	0	39 $\times 10^{-5}$	0.715
**CumSeg**	**100**	0	0	0	39 $\times 10^{-5}$	0.115
CPM.*l*.500	0	0	0	100	2957 $\times 10^{-5}$	0.011
CPM.*l*.3000	28	6	39	27	628 $\times 10^{-5}$	0.031
WBSC1	15	18	20	47	653 $\times 10^{-5}$	0.149
**WBSIC**	**99**	1	0	0	44 $\times 10^{-5}$	0.149
WBS2	89	5	4	2	82 $\times 10^{-5}$	0.958
**NOT**	**99**	1	0	0	44 $\times 10^{-5}$	0.089
**FDR**	**96**	4	0	0	47 $\times 10^{-5}$	–
**TGUH**	**100**	0	0	0	39 $\times 10^{-5}$	0.217
**ID**	**100**	0	0	0	39 $\times 10^{-5}$	0.172
**ID.SDLL**	**90**	4	0	6	182 $\times 10^{-5}$	0.069
$\mathbf{ID }_{\sqrt{3/2}}$	**99**	0	1	0	41 $\times 10^{-5}$	0.259

Also the average MSE and computational times for each method are given

**Table 6:** Distribution of ${\hat{N}} - N$ over 100 simulated data sequences from the continuous piecewise-linear signals (W1), (W3), and (W4)

Method	Model								MSE	$d_H$	Time (s)
		$${\hat{N}} - N$$									
		$\le -3$	$-2$	$-1$	0	1	2	$\ge 3$			
**NOT**		0	0	0	**99**	1	0	0	0.016	0.063	0.343
TF		0	0	0	0	0	0	100	0.029	0.451	1.125
**CPOP**		0	0	0	**99**	1	0	0	0.013	0.055	23.190
MARS	(W1)	0	0	2	9	42	39	8	0.034	0.200	0.011
FKS		0	0	0	72	22	6	0	0.015	0.109	270.385
**ID**		0	0	0	**91**	9	0	0	0.030	0.104	0.036
**ID.SDLL**		0	0	0	**98**	0	1	1	0.033	0.098	0.030
NOT		0	0	27	0	6	18	49	0.035	0.571	0.163
TF		0	0	0	0	0	0	100	606.523	0.432	0.117
**CPOP**		0	0	0	**90**	6	2	2	0.010	0.097	0.078
MARS	(W3)	91	0	7	2	0	0	0	3.991	2.258	0.008
**FKS**		0	0	0	**90**	9	1	0	0.010	0.097	67.582
**ID**		0	0	0	**99**	1	0	0	0.013	0.101	0.017
**ID.SDLL**		0	0	0	**93**	4	1	2	0.022	0.130	0.010
NOT		0	1	14	20	16	20	29	0.109	0.998	0.958
TF		0	0	0	0	0	0	100	660.399	0.465	1.349
**CPOP**	(W4)	0	0	0	**92**	8	0	0	0.015	0.084	1.627
MARS		100	0	0	0	0	0	0	22.058	1.609	0.019
**ID**		0	0	0	**92**	8	0	0	0.038	0.123	0.045
**ID.SDLL**		0	0	0	**92**	4	1	3	0.062	0.120	0.025

The average MSE, $d_H$ and computational time for each method are also given

**Table 7:** Distribution of ${\hat{N}} - N$ over 100 simulated data sequences of the continuous piecewise-linear signal (W2)

Method								MSE	$d_H$	Time (s)
	$${\hat{N}} - N$$									
	$\le -90$	$(-90,-1)$	$-1$	0	1	(1, 60]	$> 60$			
NOT	100	0	0	0	0	0	0	4.731	99	0.869
TF	0	0	0	0	0	0	100	212.547	0.387	0.863
**CPOP**	0	0	0	**97**	3	0	0	0.162	0.189	1.161
MARS	100	0	0	0	0	0	0	4.703	98.523	0.009
**ID**	0	0	0	**98**	2	0	0	0.201	0.242	0.589
**ID.SDLL**	0	0	0	**98**	2	0	0	0.256	0.287	0.097

The average MSE, $d_H$ and computational time for each method are also given

**Table 8:** ID results for the distribution of ${\hat{N}} - N$ for the models (M2)–(M4) and (W1), over 100 simulations where the distribution of the noise is Student-$t_d$, for $d=3,5$

*d*	Model								MSE	$d_H$	Time (ms)
		$${\hat{N}} - N$$									
		$\le -3$	$-2$	$-1$	0	1	2	$\ge 3$			
5	(M2)	6	2	2	74	9	5	2	$60\times 10^{-3}$	0.86	9.7
	(M3)	0	0	0	75	16	5	4	$21 \times 10^{-3}$	0.16	9.2
	(W1)	0	0	0	86	12	2	0	$31 \times 10^{-3}$	0.23	32.8
3	(M2)	7	1	2	52	21	8	9	$71\times 10^{-3}$	1.18	8.7
	(M3)	0	1	0	59	20	13	7	$26 \times 10^{-3}$	0.22	9.8
	(W1)	0	0	0	62	28	4	6	$32 \times 10^{-3}$	0.25	22.6

The average MSE, $d_H$ and computational time are also given

With regards to piecewise-constancy, ID is always in the top $10\%$ of the best methods when considering accuracy in any aspect (estimation of *N*, MSE, $d_H$); in most cases it is the best method overall. ID.SDLL is also, in most cases, in the top 10% of the best performing methods; this provides evidence that the Isolate-Detect algorithm can be combined with various model selection criteria (thresholding, SIC, SDLL) and maintain a good practical behaviour. When the threshold constant, *C*, is equal to $\sqrt{3/2}$, the behaviour of ID remains good for signals that have a moderate number of change-points that are not near each other. As we can see from Table 4, $\hbox {ID}_{ \sqrt{3/2}}$ seems to struggle in scenarios with a large number of frequently occurring change-points. In continuous piecewise-linear signals, CPOP, ID, and ID.SDLL are in all cases in the top $10\%$ of the best methods in terms of the accurate estimation of *N*. In terms of the MSE and $d_H$, CPOP is by a narrow margin the overall best method, with ID and ID.SDLL coming second and third, respectively. We can deduce that our method exhibits uniformity in detecting with high accuracy the change-points for various different signal structures, a characteristic which is at least partly absent from the majority of its competitors. Furthermore, ID’s behaviour is particularly impressive in extremely long signals with a large number of frequently occurring change-points; see Tables 4 and 7. Compared to other well-behaved methods, such as NOT for piecewise-constancy and CPOP for continuous piecewise-linear signals, our methodology has by far the lowest computational cost. To conclude, the simulation study provides evidence that Isolate-Detect is an accurate, reliable, and quick method for generalized change-point detection.

The results of Table 8 are very good for $d=5$ and not too different from those under Gaussian noise. For $d=3$, there is a slight overestimation of the number of change-points. When the tails of the distribution of the noise are significantly heavier than those of the normal distribution, one can obtain better results by increasing the threshold constant. For example, the results in Table 8 for $d=3$ were improved when the threshold constant was slightly increased. We highlight that more thorough simulations can be done using our R packages **IDetect** and **breakfast** and code available from https://github.com/Anastasiou-Andreas/IDetect/blob/master/R/Simulations_used.R.

## Real data examples

### UK House Price Index

**Fig. 6:**
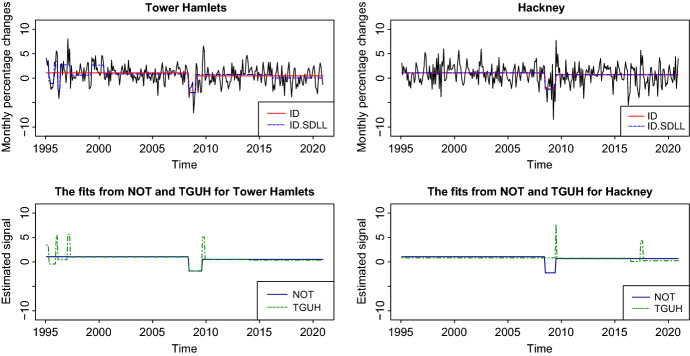
Top row: The time series and the fitted piecewise-constant mean signals obtained by ID and ID.SDLL for both Tower Hamlets and Hackney. Bottom row: NOT (solid) and TGUH (dashed) estimates for Tower Hamlets and Hackney

We investigate the performance of ID on monthly percentage changes in the UK House price index from January 1995 to December 2020 in two London Boroughs: Tower Hamlets and Hackney. The data are available from http://landregistry.data.gov.uk/app/ukhpi and they were accessed in March 2021. Figure 6 shows the fits of ID, ID.SDLL, NOT, and TGUH. In both data sets, ID behaves similarly to NOT whereas ID.SDLL’s performance is closer to that of TGUH where we detect more change-points. This difference between the examined methods is, in our opinion, due to the fact that ID in this example and NOT detect change-points based on the Schwarz Information Criterion, so fewer estimated change-points can be expected. The detection of two change-points near March 2008 and September 2009 for both boroughs may be related to the financial crisis during that time, which led to a decrease in house prices. As explained in Sect. 3.3, our methodology returns the solution path defined in (10), which can be used to obtain different fits; see Section 7 in the supplement for more details and for a real-data example where this is useful.

Residual diagnostics have indicated that the behaviour of the raw residuals, $X_t - {\hat{f}}_t$, in relation to normality and independence is good for all methods.

### The COVID-19 outbreak in the UK

**Fig. 7:**
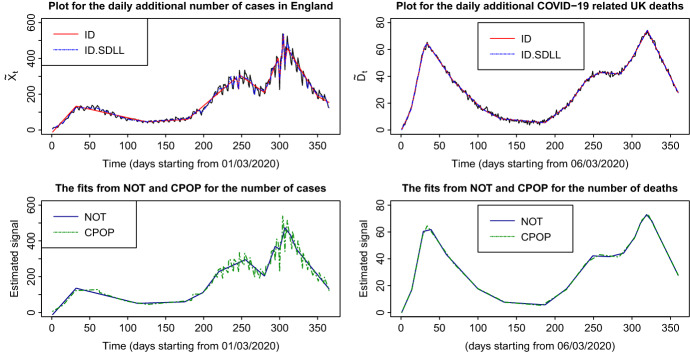
Top row: The transformed data sequence and the fitted continuous and piecewise-linear mean signals obtained by ID and ID.SDLL for both the daily number of cases and the daily number of deaths. Bottom row: NOT (solid) and CPOP (dashed) estimates for the daily number of cases and the daily number of deaths

The performance of ID is investigated on data from the recent COVID-19 pandemic; we employ a continuous piecewise-linear model on the daily number of lab-confirmed cases in England, as well as on the daily additional COVID-19 associated UK deaths. The data concern the period from the beginning of March 2020 until the end of February 2021 and they are available from https://coronavirus.data.gov.uk. The data were accessed on the 8th of March 2021. Before applying the various methods to the data, we bring the distribution closer to Gaussian with constant variance. To achieve this we perform the Anscombe transform, $a:{\mathbb {N}} \rightarrow {\mathbb {R}}$, with $a(x) = 2\sqrt{x+3/8}$ as described in Anscombe (1948). We denote the transformed number of COVID-19 cases by ${\tilde{X}}_t$ and the transformed number of COVID-19 associated deaths by ${\tilde{D}}_t$. Figure 7 presents the results of ID, ID.SDLL, CPOP, and NOT for the transformed data. We observe that ID, ID.SDLL, and NOT have a similar behaviour, while CPOP gives a higher estimated number of change-points. In an attempt to date the detected change-points by ID, we provide a possible explanation of their location with respect to the outbreak of the pandemic in the UK; this discussion is given in Section 4 of the supplementary material.

For another example related to the continuous, piecewise-linear case, see Section 7 of the supplement where we explore the behaviour of Isolate-Detect and two competitors, CPOP and NOT, on the daily closing stock prices of Samsung Electronics Co. from July 2012 until June 2020.

## Concluding reflections on ID

In this paper, we have proposed Isolate-Detect which is a new, generic technique for multiple generalized change-point detection in noisy data sequences. The method is based on a change-point isolation approach which seems to provide an advantage in detection power, especially in complex structures where most state-of-the-art competitors seem to suffer (see the simulations in Sect. 5) such as limited spacings between change-points. In addition, the aforementioned isolation aspect allows the extension of our method to the detection of knots in higher-order polynomial signals. As already mentioned in Sect. 1, NOT, WBS, and WBS2 also work on sub-intervals of the data, but the way the isolation is carried out in ID, where one of the end-points of the subintervals is kept fixed, provides predictable execution times for the analysis of a given data sequence, which are faster than the aforementioned competitors; see Sects. 4.1 and 5. Another advantage of our method over NOT, WBS and WBS2 is that, due to its pseudo-sequential interval expansion character, it can easily be applied for online change-point detection.

In Sect. 4.4, a variant of ID was introduced that combines the threshold- and SIC-based versions of our proposed method with the aim to enhance its accuracy (both in terms of the estimated number and the estimated change-point locations) for signals of different structures with respect to the true number of change-points and the distance between them. In addition, due to the way that the relevant hybrid approach has been developed in Sect. 4.4, we manage to offer, for ease of execution, minimal parameter choice. Apart from thresholding and SIC, we have also combined ID with the SDLL model selection criterion.

In the practical applications of Sects. 5 and 6, compared to the state-of-the-art competitors, ID lies in the top 10% (in terms of the accurate estimation of the number and the location of the change-points) of the best methods. Furthermore, it exhibits a notable advantage over other techniques in long signals with many change-points that occur frequently. In addition, ID’s pseudo-sequential character assists in attaining a low computational time; our method can accurately analyse signals of tens of thousands with thousands of change-points in less than a second; see for example Table 4. In cases where the normality assumption for the error terms is violated, Sect. 4.5 provides a practical solution where pre-processing allows us to use ID without altering the proposed parameter values. The results of simulations from a Student-*t* distribution with two options for the degrees of freedom are in Table 8.

Since no method has a uniformly best behaviour, it is natural to also highlight the weaknesses of our method in terms of its practical behaviour. To start with, ID can be slow in long and constant signals in which change-points do not occur. This is because of the expanding intervals attribute, which in the case of no change-points will push the method to keep testing for change-points in growing, overlapping intervals. This is inevitably going to lead to high computational costs. We tried to eliminate this weakness by introducing a window-based variant, as explained in Section 3. Another drawback of the method is that, due to its left- and right-expanding feature, the change-points need to be detectable based on relatively unbalanced intervals. This could lead to accuracy issues in signals where the change-points are in the middle of the data sequence and offset each other. In practice, we have not encountered this type of behaviour in ID; in particular it accurately detects the change-points for the model (M4) in Table 3, which is an example of the aforementioned structure with two nearby change-points in the middle of the data sequence.

## Supplementary Information

Below is the link to the electronic supplementary material.

Supplementary material 1 (pdf 569 KB)

## References

[CR1] Anscombe FJ (1948). The transformation of Poisson, binomial and negative-binomial data. Biometrika.

[CR2] Auger IE, Lawrence CE (1989). Algorithms for the optimal identification of segment neighborhoods. Bull Math Biol.

[CR3] Bai J, Perron P (1998). Estimating and testing linear models with multiple structural changes. Econometrica.

[CR4] Baranowski R, Chen Y, Fryzlewicz P (2019). Narrowest-over-threshold detection of multiple change points and change-point-like features. J R Stat Soc B.

[CR5] Chan HP, Walther G (2013). Detection with the scan and the average likelihood ratio. Stat Sin.

[CR6] Cho H, Kirch C (2020) Data segmentation algorithms: univariate mean change and beyond. arXiv:2012.12814

[CR7] Dette H, Eckle T, Vetter M (2020). Multiscale change point detection for dependent data. Scand J Stat.

[CR8] Eichinger B, Kirch C (2018). A MOSUM procedure for the estimation of multiple random change points. Bernoulli.

[CR9] Fang X, Siegmund D (2020) Detection and Estimation of Local Signals. arXiv:2004.08159

[CR10] Fang X, Li J, Siegmund D (2020). Segmentation and estimation of change-point models: false positive control and confidence regions. Ann Stat.

[CR11] Fearnhead P, Rigaill G (2020). Relating and comparing methods for detecting changes in mean. Stat.

[CR12] Fearnhead P, Maidstone R, Letchford A (2019). Detecting changes in slope with an $${L}_0$$ penalty. J Comput Graph Stat.

[CR13] Frick K, Munk A, Sieling H (2014). Multiscale change point inference. J R Stat Soc B.

[CR14] Friedman JH (1991). Multivariate adaptive regression splines. Ann Stat.

[CR15] Fryzlewicz P (2014). Wild binary segmentation for multiple change-point detection. Ann Stat.

[CR16] Fryzlewicz P (2018). Tail-greedy bottom-up data decompositions and fast multiple change-point detection. Ann Stat.

[CR17] Fryzlewicz P (2020). Detecting possibly frequent change-points: wild binary segmentation 2 and steepest-drop model selection. J Korean Stat Soc.

[CR18] Hampel FR (1974). The influence curve and its role in robust estimation. J Am Stat Assoc.

[CR19] Haynes K, Fearnhead P, Eckley IA (2017). A computationally efficient nonparametric approach for changepoint detection. Stat Comput.

[CR20] Jackson B, Sargle JD, Barnes D, Arabhi S, Alt A, Gioumousis P, Gwin E, Sangtrakulcharoen P, Tan L, Tsai TT (2005). An algorithm for optimal partitioning of data on an interval. IEEE Signal Process Lett.

[CR21] Killick R, Fearnhead P, Eckley IA (2012). Optimal detection of changepoints with a linear computational cost. J Am Stat Assoc.

[CR22] Kim S-J, Koh K, Boyd S, Gorinevsky D (2009). $$\ell _1$$ trend filtering. SIAM Rev.

[CR23] Kovács S, Li H, Bühlmann P, Munk A (2020) Seeded binary segmentation: a general methodology for fast and optimal change point detection. arXiv:2002.06633

[CR24] Li H, Munk A, Sieling H (2016). FDR-control in multiscale change-point segmentation. Electron J Stat.

[CR25] Liu J, Wu S, Zidek JV (1997). On segmented multivariate regression. Stat Sin.

[CR26] Maidstone R, Hocking T, Rigaill G, Fearnhead P (2017). On optimal multiple changepoint algorithms for large data. Stat Comput.

[CR27] Muggeo VMR, Adelfio G (2011). Efficient change point detection for genomic sequences of continuous measurements. Bioinformatics.

[CR28] Olshen AB, Venkatraman ES, Lucito R, Wigler M (2004). Circular binary segmentation for the analysis of array-based DNA copy number data. Biostatistics.

[CR29] Raimondo M (1998). Minimax estimation of sharp change points. Ann Stat.

[CR30] Rigaill G (2015). A pruned dynamic programming algorithm to recover the best segmentations with 1 to $${K}_{max}$$ change-points. Journal de la Société Française de Statistique.

[CR31] Ross GJ (2015). Parametric and nonparametric sequential change detection in R: the cpm package. J Stat Softw.

[CR32] Rousseeuw PJ, Croux C (1993). Alternatives to the median absolute deviation. J Am Stat Assoc.

[CR33] Spiriti S, Eubank R, Smith PW, Young D (2013). Knot selection for least-squares and penalized splines. J Stat Comput Simul.

[CR34] Tibshirani RJ (2014). Adaptive piecewise polynomial estimation via trend filtering. Ann Stat.

[CR35] Truong C, Oudre L, Vayatis N (2020). Selective review of offline change point detection methods. Signal Process.

[CR36] Venkatraman ES (1992) Consistency results in multiple change-point problems. Ph.D. thesis, Stanford University

[CR37] Vostrikova L (1981). Detecting “disorder” in multidimensional random processes. Sov Math Dokl.

[CR38] Yao Y-C (1988). Estimating the number of change-points via Schwarz’ criterion. Stat Probab Lett.

[CR39] Yu Y (2020) A review on minimax rates in change point detection and localisation. arXiv:2011.01857

