# Supplementary material for: Detecting multiple generalized change-points by isolating single ones
Source: Metrika. 2021 May 24;85(2):141–74. doi: 10.1007/s00184-021-00821-6 (PMC8142888; doi:10.1007/s00184-021-00821-6)
Supplement: Supplementary file 1 — Supplementary material 1 (pdf 569 KB) [file 184_2021_821_MOESM1_ESM.pdf]

# Supplement to "Detecting multiple generalized change-points by isolating single ones"

By A. ANASTASIOU[1,†] and P. FRYZLEWICZ[2,‡]

[1]*Department of Mathematics and Statistics, University of Cyprus, P.O. Box: 20537, 1678, Nicosia, Cyprus*
[2]*Department of Statistics, The London School of Economics and Political Science, Columbia House, Houghton Street, London, WC2A 2AE*
*E-mail:* [†]anastasiou.andreas@ucy.ac.cy; [‡]p.fryzlewicz@lse.ac.uk

## 1 The different parts in the removal process of the solution path algorithm

The four different parts of the removal process applied in order to create the solution path explained in Section 3.3 of the paper are very similar and are based on the idea of removing change-points according to their contrast function values as well as their distance to neighbouring estimates. We mention that if the algorithm proceeds from Part 1 to Part 2 as below, then it is guaranteed that it will also proceed up to Part 4. All events below occur with probability tending to one with $T$.

**Part 1:** With $C^*$ being a positive constant, the aim is to prune the estimates in $\tilde{S}$, such that, for each true change-point, there are at most four and at least one estimated change-point within a distance of $C^*(\log T)^\alpha$. To achieve this, $\forall j \in \{1, 2, \ldots, J\}$ and with $\tilde{r}_0 = 1$, $\tilde{r}_{J+1} = T$, we collect triplets $(\tilde{r}_{j-1}, \tilde{r}_j, \tilde{r}_{j+1})$ and we calculate $CS(\tilde{r}_j) := C^{\tilde{r}_j}_{\tilde{r}_{j-1},\tilde{r}_{j+1}}(\boldsymbol{X})$, with $C^b_{s,e}(\boldsymbol{X})$ being the relevant contrast function. For $m = \mathrm{argmin}_j \{CS(\tilde{r}_j)\}$, firstly we check whether $CS(\tilde{r}_m) \leq \tilde{\tilde{C}}\sqrt{\log T}$, for $\tilde{\tilde{C}} > 0$; in the proofs of Theorems 3 and 4, $\tilde{\tilde{C}} = 2\sqrt{2}$, but smaller values could be sufficient. If $CS(\tilde{r}_m) \leq \tilde{\tilde{C}}\sqrt{\log T}$ and also $\tilde{r}_{j+1} - \tilde{r}_{j-1} \leq 2C^*(\log T)^\alpha$, we remove $\tilde{r}_m$ from $\tilde{S}$, reduce $J$ by 1, relabel the remaining estimates (in increasing order) in $\tilde{S}$, and repeat this estimate removal process. We proceed to Part 2 when $CS(\tilde{r}_m) > \tilde{\tilde{C}}\sqrt{\log T}$. If this is not satisfied at any point of this part, then we conclude that there are no change-points in the data sequence and we stop.

**Part 2:** The aim is to continue the pruning process of Part 1, in a way that at the end of Part 2 there is at least one estimate within a distance of $C^*(\log T)^\alpha$ from each true change-point, but also there are at most two estimates between any pair of consecutive true change-points. For the relabelled estimates in $\tilde{S}$ after the completion of Part 1, if $\tilde{r}_j - \tilde{r}_{j-1} \leq C^*(\log T)^\alpha$, then we remove $\tilde{r}_j$, relabel the remaining estimates, and keep removing the estimates until there is no pair $(\tilde{r}_{j-1}, \tilde{r}_j)$, such that $\tilde{r}_j - \tilde{r}_{j-1} \leq C^*(\log T)^\alpha$. We then calculate $CS(\tilde{r}_j)$ as in Part 1 and for $m = \mathrm{argmin}_j \{CS(\tilde{r}_j)\}$, if $CS(\tilde{r}_m) \leq \tilde{\tilde{C}}\sqrt{\log T}$, then we remove $\tilde{r}_m$ and relabel the remaining elements of $\tilde{S}$. This removal process is repeated and we proceed to Part 3 only when $CS(\tilde{r}_m) > \tilde{\tilde{C}}\sqrt{\log T}$.

**Part 3:** We need to ensure that once $\tilde{S}$ contains $N$ estimates, then for $j = 1, 2, \ldots, N$, each $\tilde{r}_j$ is within a distance of $C^* (\log T)^{\alpha}$ from $r_j$. To achieve this, for the remaining estimated change-points after Part 2, we use triplets $(\tilde{s}_j, \tilde{r}_j, \tilde{e}_j)$, with $\tilde{s}_j = \lfloor (\tilde{r}_{j-1} + \tilde{r}_j)/2 \rfloor + 1$ and $\tilde{e}_j = \lceil (\tilde{r}_j + \tilde{r}_{j+1})/2 \rceil$. For $m = \text{argmin}_j C^{\tilde{r}_j}_{\tilde{s}_j, \tilde{e}_j}(\boldsymbol{X})$, if $C^{\tilde{r}_m}_{\tilde{s}_m, \tilde{e}_m}(\boldsymbol{X}) \leq \tilde{\tilde{C}} \sqrt{\log T}$, then we remove $\tilde{r}_m$ and relabel the remaining estimates in $\tilde{S}$ in increasing order. We repeat this removal procedure until $C^{\tilde{r}_m}_{\tilde{s}_m, \tilde{e}_m}(\boldsymbol{X}) > \tilde{\tilde{C}} \sqrt{\log T}$, which is when we proceed to Part 4.

**Part 4:** For the estimated change-points that are in $\tilde{S}$ after Part 3 is completed, we use again the triplets $(\tilde{r}_{j-1}, \tilde{r}_j, \tilde{r}_{j+1})$ in order to find $m = \text{argmin}_j \{CS(\tilde{r}_j)\}$ and then remove $\tilde{r}_m$ from $\tilde{S}$. This estimates removal approach is repeated until $\tilde{S} = \emptyset$.

## 2 Models used in the simulation study of the paper

The characteristics of the test signals $f_t$ as well as the standard deviations $\sigma$ of the noise $\epsilon_t$, which were used in the simulation study are given in the list below.

(M1) *blocks*: length 2048 with change-points at 205, 267, 308, 472, 512, 820, 902, 1332, 1557, 1598, 1659 with values between change-points 0, 14.64, −3.66, 7.32, −7.32, 10.98, −4.39, 3.29, 19.03, 7.68, 15.37, 0. The standard deviation is $\sigma = 10$.

(M2) *teeth*: length 140 with change-points at 11, 21, 31, 41, 51, 61, 71, 81, 91, 101, 111, 121, 131 with values between change-points 0, 1, 0, 1, 0, 1, 0, 1, 0, 1, 0, 1, 0, 1. The standard deviation of the noise is $\sigma = 0.4$.

(M3) *stairs*: length 150 with change-points at 11, 21, 31, 41, 51, 61, 71, 81, 91, 101, 111, 121, 131, 141 with values between change-points 1, 2, 3, 4, 5, 6, 7, 8, 9, 10, 11, 12, 13, 14, 15. The standard deviation of the noise is $\sigma = 0.3$.

(M4) *middle-points*: length 2000 with change-points at 1000 and 1020 with values between change-points 0, 1.5, 0. The standard deviation of the noise is $\sigma = 1$.

(M5) *long teeth*: length 20000 with 1999 change-points at $10, 20, \ldots, 19990$ with values between change-points $0, 3, 0, 3, \ldots, 0, 3$. The standard deviation is $\sigma = 0.8$.

(NC) *constant signal*: length 3000 with no change-points. The standard deviation is $\sigma = 1$.

(W1) *wave 1*: piecewise-linear signal without jumps in the intercept, $T = 1408$, with 7 change-points at $256, 512, 768, 1024, 1152, 1280, 1344$ with the corresponding changes in slopes $-1/64, 2/64, -3/64, 4/64, -5/64, 6/64, -7/64$, starting intercept $f_1 = 1$ and slope $f_2 - f_1 = 1/256$. The standard deviation of the noise is $\sigma = 1$.

(W2) *wave 2*: piecewise-linear signal without jumps in the intercept, $T = 1500$, with 99 change-points at $15, 30, \ldots, 1485$. The corresponding changes in the slope are $-1, 1, -1, \ldots, -1$, while the starting intercept is $f_1 = -1/2$ and the starting slope is $f_2 - f_1 = 1/40$. The standard deviation is $\sigma = 1$.

(W3) *smoother signal 1*: piecewise-linear signal without jumps in the intercept, $T = 200$, with 9 change-points at $20, 40, \ldots, 180$ with the corresponding changes in

slopes $1/6, 1/2, -3/4, -1/3, -2/3, 1, 1/4, 3/4, -5/4$. The starting intercept is $f_1 = 1$ and slope $f_2 - f_1 = 1/32$. The standard deviation of the noise is $\sigma = 0.3$.

(W4) *smoother signal 2*: piecewise-linear signal without jumps in the intercept, $T = 1000$, with 19 change-points at $50, 100, \ldots, 950$ with the corresponding changes in slopes $-1/16, -5/16, -5/8, 1, 5/16, 15/32, -5/8, -7/32, -3/4, 13/16, 5/16, 19/32, -1, -5/8, 23/32, 1/2, 15/16, -25/16, -5/4$, starting intercept $f_1 = 1$ and slope $1/32$. The standard deviation of the noise is $\sigma = 0.6$.

## 3 Improvement of ID in the case of big data

In this section, we show through simulations that applying ID on a fixed window grid improves its speed in large data sets, without affecting its accuracy. We compare the classic ID method as explained in Section 3.1 of the main paper with the new window-grid-based version (WID) of Section 4.3 in the case of three data sequences of length $10^5$, each with standard Gaussian noise. We work under the scenario of piecewise-constant mean. The three signals are

(D1) No change-points;

(D2) three change-points at 25000, 55000, 85000 and the values between change-points are 0,3,-3,2;

(D3) seven change-points at 16000, 22000, 28000, 46000, 62000, 74000, 86000 and the values between change-points are 0,4,-4,4,-4,4,-4,4.

We took the expansion parameter $\lambda_T$ to be equal to 10 and the results are shown in Table 1. As a measure of the accuracy of the detected locations, we provide Monte-Carlo estimates of the mean squared error, $\mathrm{MSE} = T^{-1}\sum_{t=1}^{T}\mathbb{E}\left(\hat{f}_t - f_t\right)^2$. The scaled Hausdorff distance,

$$d_H = n_s^{-1}\max\left\{\max_j \min_k |r_j - \hat{r}_k|, \max_k \min_j |r_j - \hat{r}_k|\right\},$$

where $n_s$ is the length of the largest segment, is also given for (D2) and (D3); for (D1), $d_H$ is not informative. In terms of accuracy, both methods exhibit excellent behaviour. However, in terms of speed, the advantage of the windows-based approach is obvious. Note the decrease in the computational time of ID when the number of change-points gets larger. This is expected because the worst case in terms of computational complexity is when there are no change-points because ID will then be forced to calculate the contrast function on quite large intervals, even on $[1, T]$, which is computationally more expensive.

**Table 1** A comparison on the performance of WID and ID over 10 simulated time series of three different models of length $10^5$ each. The distribution of $\hat{N} - N$, as well as the average MSE, Hausdorff distance and computational time for each method are provided

| Method | Model | $\hat{N} - N = 0$ | MSE | $d_H$ | Time (s) |
|---|---|---|---|---|---|
| WID | | 10 | $1.65 \times 10^{-5}$ | - | 2.39 |
| ID | (D1) | 10 | $1.65 \times 10^{-5}$ | - | 79.40 |
| WID | | 10 | $8.8 \times 10^{-5}$ | $3.8 \times 10^{-5}$ | 2.28 |
| ID | (D2) | 10 | $11 \times 10^{-5}$ | $1.6 \times 10^{-5}$ | 13.06 |
| WID | | 10 | $8.9 \times 10^{-5}$ | 0 | 2.17 |
| ID | (D3) | 10 | $8.9 \times 10^{-5}$ | 0 | 6.34 |

## 4 A justification of the estimations in the example of Section 6.2

Here, we provide a possible explanation of the three most important (based on the solution path) detected change-points by ID in the real data related to the COVID-19 outbreak in the UK. We focus on the signal obtained with respect to the daily number of deaths (ID and ID.SDLL give exactly the same outcome), but similar conclusions can be extracted for the daily number of cases. The three most important changes in the behaviour of the data sequence have been detected on the $8^{th}$ of April, the $12^{th}$ of July and the $19^{th}$ of January. Some justification of these results is as follows:

- The change-point on the $8^{th}$ of April, shows that the upward trend vanishes and a negative slope takes its place leading to a vast decrease in the number of reported deaths. There is an obvious connection of this change-point with the British Governments important decision in late March to impose a general lockdown. On 9 April, the Secretary of State for Foreign Affairs stated that the UK was *starting to see the impact of the restrictions*; our second detection is in full agreement with the aforementioned date and statement.
- With respect to the change-point on the $12^{th}$ of July, it indicates a stabilisation, at a low level, for the number of deaths. This is, of course, expected because the downward trend could not continue indefinitely.
- The last change-point on the $19^{th}$ of January indicates that an upward trend on the daily number of deaths, which was apparent for a period of almost 3.5 months, vanishes and its place takes a significantly negative slope. There is an obvious connection of this change-point with the vaccination programme in the country. More specifically, on 8 January 2021, MRNA-1273 (commonly known as the Moderna vaccine) was the third COVID-19 vaccine approved for use in the UK.

## 5 Additional simulation results

Here we present the results of simulations for various signals other than those presented in Section 5 of the main article. Tables 3-7 summarize the results for the following models.

(LS) *long stairs*: length 10000 with 499 change-points at $20, 40, \ldots, 9980$ with values between change-points $0, 2, 4, 6, \ldots, 996, 998$. The standard deviation is $\sigma = 1$.

(LT2) *long teeth 2*: length 10000 with 249 change-points at $40, 80, \ldots, 9960$ with values between change-points $0, 1.5, 0, 1.5, \ldots, 0, 1.5$. The standard deviation is $\sigma = 1$.

(ELT) *extremely long teeth*: length 100000 with 19999 change-points at $5, 10, \ldots, 99995$ with values between change-points $0, 2, 0, 2, \ldots, 0, 2$. The standard deviation is $\sigma = 0.3$.

(NC2) *constant signal 2*: length 300 with no change-points. The standard deviation is $\sigma = 1$.

(SW1) *wave 5*: piecewise-linear signal without jumps in the intercept, $T = 2400$, with 119 change-points at $20, 40, \ldots, 2380$ with the corresponding changes in slopes $2.5, -2.5, 2.5, \ldots, 2.5$, starting intercept $f_1 = 1$, slope $f_2 - f_1 = 1.25$ and $\sigma = 3$.

(SW2) *wave 6*: piecewise-linear signal without jumps in the intercept, $T = 1500$, with 29 change-points at $50, 100, \ldots, 1450$ with the corresponding changes in slopes $-1/7, 1/7, -1/7, \ldots, -1/7$, starting intercept $f_1 = -1/2$, slope $f_2 - f_1 = 1/24$ and $\sigma = 1$.

(SW3) *wave 7*: piecewise-linear signal without jumps in the intercept, $T = 840$, with 119 change-points at $7, 14, \ldots, 833$ with the corresponding changes in slopes $-1, 1, -1, \ldots, -1$, starting intercept $f_1 = -1/2$ and slope $f_2 - f_1 = 1/32$. The standard deviation is $\sigma = 0.3$.

The signals (LS), (LT2), (ELT), and (NC2) are treated under piecewise-constancy, while (SW1), (SW2) and (SW3) under the continuous and piecewise-linear case. FDR, WBSIC and S3IB are excluded from the comparative study for the extremely long signal (ELT). For FDR, we had to interrupt the execution after 10 hours, while for WBSIC and S3IB, in order to have a fair comparison of the methods with the rest, we had to increase the default value of the maximum number of change-points allowed to be detected to be greater than 20000. For a single iteration we had to stop the execution for WBSIC and S3IB after 30 minutes.

**Table 2** Distribution of $\hat{N} - N$ over 100 simulated data sequences from the piecewise-constant signal (LS). The average MSE, $d_H$ and computational time are also given

| | $\hat{N} - N$ | | | | | | | |
|---|---|---|---|---|---|---|---|---|
| Method | $\leq -300$ | $(-300, -100]$ | $(-100, -10)$ | $[-10, 10]$ | $> 10$ | MSE | $d_H$ | Time (s) |
| PELT | 0 | 100 | 0 | 0 | 0 | 0.67 | 1.02 | 0.024 |
| NP.PELT | 100 | 0 | 0 | 0 | 0 | 9655.28 | 113.76 | 11.302 |
| S3IB | 0 | 13 | 87 | 0 | 0 | 0.44 | 1.01 | 111.143 |
| CumSeg | 100 | 0 | 0 | 0 | 0 | 87.11 | 15.08 | 0.323 |
| **CPM.$l$.500** | 0 | 0 | 0 | **100** | 0 | 0.19 | 0.75 | 0.002 |
| CPM.$l$.10000 | 0 | 0 | 74 | 25 | 0 | 0.22 | 1 | 0.002 |
| WBSC1 | 0 | 0 | 80 | 20 | 0 | 0.24 | 1 | 1.293 |
| WBSIC | 0 | 0 | 22 | 78 | 0 | 0.22 | 0.99 | 1.293 |
| WBS2 | 0 | 0 | 29 | 58 | 13 | 0.22 | 0.99 | 1.293 |
| NOT | 100 | 0 | 0 | 0 | 0 | 9.31 | 5.27 | 4.330 |
| **FDR** | 0 | 0 | 2 | **98** | 0 | 0.19 | 0.83 | - |
| TGUH | 0 | 0 | 100 | 0 | 0 | 0.29 | 1 | 0.484 |
| **ID** | 0 | 0 | 14 | **86** | 0 | 0.21 | 1.00 | 0.460 |

**Table 3** Distribution of $\hat{N} - N$ over 100 simulated data sequences from the piecewise-constant signal (LT2). The average MSE, $d_H$ and computational time are also given

| Method | $\hat{N} - N$ | | | | | MSE | $d_H$ | Time (s) |
|---|---|---|---|---|---|---|---|---|
| | $\leq -150$ | $(-150, -50]$ | $(-50, -10)$ | $[-10, 10]$ | $> 10$ | | | |
| PELT | 100 | 0 | 0 | 0 | 0 | 0.55 | 130.64 | 0.016 |
| NP.PELT | 0 | 12 | 88 | 0 | 0 | 0.21 | 4.95 | 0.496 |
| S3IB | 0 | 1 | 54 | 45 | 0 | 0.13 | 2.58 | 33.410 |
| CumSeg | 100 | 0 | 0 | 0 | 0 | 0.56 | 249 | 0.428 |
| CPM.$l$.500 | 0 | 0 | 0 | 9 | 91 | 0.12 | 0.59 | 0.008 |
| **CPM.$l$.10000** | 0 | 0 | 0 | **100** | 0 | 0.12 | 1.21 | 0.008 |
| WBSC1 | 0 | 84 | 16 | 0 | 0 | 0.27 | 4.22 | 0.631 |
| WBSIC | 7 | 0 | 0 | 88 | 5 | 0.16 | 15.92 | 1.051 |
| NOT | 100 | 0 | 0 | 0 | 0 | 0.56 | 240.08 | 0.610 |
| **FDR** | 0 | 0 | 0 | **99** | 1 | 0.11 | 0.72 | - |
| **TGUH** | 0 | 0 | 2 | **98** | 0 | 0.14 | 1.43 | 0.580 |
| **ID** | 0 | 0 | 0 | **100** | 0 | 0.11 | 0.76 | 0.139 |

**Table 4** Distribution of $\hat{N} - N$ over 100 simulated time series from the signal (ELT). Also the average MSE and computational times for each method are given

| Method | $\hat{N} - N$ | | | MSE | Time (s) |
|---|---|---|---|---|---|
| | $\leq -17000$ | $(-17000, -10)$ | $[-10, 10]$ | | |
| PELT | 100 | 0 | 0 | 0.94 | 0.086 |
| NP.PELT | 100 | 0 | 0 | 1 | 136.154 |
| CumSeg | 100 | 0 | 0 | 1 | 4.831 |
| CPM.$l$.500 | 100 | 0 | 0 | 1 | 59.936 |
| WBSC1 | 100 | 0 | 0 | 0.92 | 6.346 |
| NOT | 100 | 0 | 0 | 1 | 2.873 |
| **TGUH** | 0 | 0 | **100** | 0.02 | 4.751 |
| **ID** | 0 | 10 | **90** | 0.02 | 3.693 |

**Table 5** Distribution of $\hat{N} - N$ over 100 simulated time series from (NC2). Also the average MSE and computational times for each method are given

| Method | $\hat{N} - N$ | | | | MSE | Time (ms) |
|---|---|---|---|---|---|---|
| | 0 | 1 | 2 | $\geq 3$ | | |
| **PELT** | **100** | 0 | 0 | 0 | 28 $\times 10^{-4}$ | 12.3 |
| NP.PELT | 56 | 13 | 23 | 8 | 269 $\times 10^{-4}$ | 30.4 |
| **S3IB** | **95** | 3 | 2 | 0 | 57 $\times 10^{-4}$ | 35.7 |
| **CumSeg** | **100** | 0 | 0 | 0 | 28 $\times 10^{-4}$ | 19.3 |
| CPM.$l$.500 | 54 | 11 | 15 | 20 | 294 $\times 10^{-4}$ | 1.5 |
| WBSC1 | 17 | 16 | 19 | 48 | 578 $\times 10^{-4}$ | 62.7 |
| **WBSIC** | **95** | 3 | 1 | 1 | 59 $\times 10^{-4}$ | 61.3 |
| **NOT** | **99** | 0 | 0 | 1 | 39 $\times 10^{-4}$ | 37.6 |
| **FDR** | **90** | 7 | 2 | 1 | 66 $\times 10^{-4}$ | - |
| TGUH | 83 | 0 | 12 | 5 | 147 $\times 10^{-4}$ | 49.6 |
| **ID** | **95** | 4 | 1 | 0 | 60 $\times 10^{-4}$ | 1.1 |

**Table 6** Distribution of $\hat{N} - N$ over 100 simulated time series of the signal (SW1). Also, the average MSE, Hausdorff distance and computational time for each method are given

| | $\hat{N} - N$ | | | | | | | | |
|---|---|---|---|---|---|---|---|---|---|
| Method | $\leq -110$ | $(-110, -1]$ | 0 | 1 | $(1, 90)$ | $\geq 90$ | MSE | $d_H$ | Time (s) |
| NOT | 100 | 0 | 0 | 0 | 0 | 0 | 52.352 | 119 | 6.061 |
| TF | 0 | 0 | 0 | 0 | 0 | 100 | 107.463 | 0.381 | 2.309 |
| **CPOP** | 0 | 0 | **96** | 4 | 0 | 0 | 1.063 | 0.146 | 3.327 |
| **ID** | 0 | 0 | **90** | 10 | 0 | 0 | 1.781 | 0.254 | 0.041 |

**Table 7** Distribution of $\hat{N} - N$ over 100 simulated time series of the signal (SW2). Also, the average MSE, Hausdorff distance and computational time for each method are given

| | $\hat{N} - N$ | | | | | | | | | | |
|---|---|---|---|---|---|---|---|---|---|---|---|
| Method | $\leq -10$ | $(-10, -1)$ | $-1$ | 0 | 1 | 2 | $(2, 10)$ | $\geq 10$ | MSE | $d_H$ | Time (s) |
| NOT | 0 | 5 | 1 | 1 | 1 | 3 | 25 | 64 | 0.24 | 0.95 | 0.412 |
| TF | 0 | 0 | 0 | 0 | 0 | 0 | 0 | 100 | 0.76 | 0.33 | 1.417 |
| **CPOP** | 0 | 0 | 0 | **97** | 3 | 0 | 0 | 0 | 0.05 | 0.17 | 8.728 |
| **ID** | 0 | 0 | 0 | **97** | 2 | 1 | 0 | 0 | 0.07 | 0.27 | 0.049 |

**Table 8** Distribution of $\hat{N} - N$ over 100 simulated data sequences of the continuous piecewise-linear signal (SW3). The average MSE, $d_H$ and computational time for each method are also given

| | $\hat{N} - N$ | | | | | | | | | |
|---|---|---|---|---|---|---|---|---|---|---|
| Method | $\leq -100$ | $(-100, -1)$ | $-1$ | 0 | 1 | $(1, 10]$ | $> 10$ | MSE | $d_H$ | Time (s) |
| NOT | 100 | 0 | 0 | 0 | 0 | 0 | 0 | 1.063 | 119 | 0.485 |
| TF | 0 | 0 | 0 | 0 | 0 | 0 | 100 | 217868.2 | 0.324 | 0.632 |
| **CPOP** | 0 | 0 | 0 | **98** | 2 | 0 | 0 | 0.027 | 0.154 | 0.438 |
| **ID** | 0 | 0 | 0 | **100** | 0 | 0 | 0 | 0.039 | 0.210 | 0.055 |

In all examples, the ID methodology is within $10\%$ of the best method. Once again, it exhibits remarkable behaviour when it comes to very long signals with a large number of frequently appearing change-points; see Tables 3, 4 and 6.

## 6 Investigation of the impact of different values of $\lambda_T$ on accuracy

In this section, we provide a small-scale simulation study in order to examine the behaviour of ID on different values of the expansion parameter $\lambda_T$. The simulation set up is as in Section 5 of the main paper and the signals used are those explained in Section 2 of the supplement. For the expansion parameter, we take $\lambda_T \in \{5, 20, 80\}$. The results are in Tables 9 - 15 below.

**Table 9** Distribution of $\hat{N} - N$ over 100 simulated data sequences from (NC). The average MSE and computational times are also given

| Method | $\hat{N} - N$: 0 | 1 | 2 | $\geq 3$ | MSE | Time (s) |
|---|---|---|---|---|---|---|
| $\text{ID}_{\lambda_T=5}$ | 100 | 0 | 0 | 0 | $31 \times 10^{-5}$ | 0.116 |
| $\text{ID}_{\lambda_T=20}$ | 100 | 0 | 0 | 0 | $31 \times 10^{-5}$ | 0.033 |
| $\text{ID}_{\lambda_T=80}$ | 100 | 0 | 0 | 0 | $31 \times 10^{-5}$ | 0.012 |

**Table 10** Distribution of $\hat{N} - N$ over 100 simulated data sequences of the piecewise-constant signals (M1)-(M3). The average MSE, $d_H$ and computational time are also given

| Method | Model | $\hat{N} - N$: $\leq -3$ | $-2$ | $-1$ | 0 | 1 | 2 | $\geq 3$ | MSE | $d_H$ | Time (ms) |
|---|---|---|---|---|---|---|---|---|---|---|---|
| $\text{ID}_{\lambda_T=5}$ | (M1) | 0 | 3 | 40 | 56 | 1 | 0 | 0 | 2.49 | 0.06 | 26.8 |
| $\text{ID}_{\lambda_T=20}$ | (M1) | 0 | 3 | 40 | 57 | 0 | 0 | 0 | 2.44 | 0.06 | 17.4 |
| $\text{ID}_{\lambda_T=80}$ | (M1) | 2 | 8 | 47 | 43 | 0 | 0 | 0 | 2.92 | 0.08 | 11.6 |
| $\text{ID}_{\lambda_T=5}$ | (M2) | 7 | 8 | 3 | 72 | 9 | 1 | 0 | $67 \times 10^{-3}$ | 0.88 | 10.9 |
| $\text{ID}_{\lambda_T=20}$ | (M2) | 83 | 7 | 8 | 4 | 0 | 0 | 0 | $185 \times 10^{-3}$ | 6.80 | 8.1 |
| $\text{ID}_{\lambda_T=80}$ | (M2) | 91 | 2 | 0 | 7 | 0 | 0 | 0 | $212 \times 10^{-3}$ | 8.75 | 5.7 |
| $\text{ID}_{\lambda_T=5}$ | (M3) | 0 | 0 | 0 | 82 | 14 | 4 | 0 | $23 \times 10^{-3}$ | 0.16 | 9.9 |
| $\text{ID}_{\lambda_T=20}$ | (M3) | 20 | 38 | 30 | 10 | 2 | 0 | 0 | $86 \times 10^{-3}$ | 0.82 | 9.2 |
| $\text{ID}_{\lambda_T=80}$ | (M3) | 100 | 0 | 0 | 0 | 0 | 0 | 0 | $784 \times 10^{-3}$ | 2.73 | 5 |

**Table 11** Distribution of $\hat{N} - N$ over 100 simulated data sequences from the piecewise-constant signal (M4). The average MSE, $d_H$ and computational time are also given

| Method | $\hat{N} - N$: $-2$ | $-1$ | 0 | 1 | $\geq 2$ | MSE | $d_H$ | Time (ms) |
|---|---|---|---|---|---|---|---|---|
| $\text{ID}_{\lambda_T=5}$ | 12 | 0 | 87 | 1 | 0 | $7 \times 10^{-3}$ | 0.13 | 31.5 |
| $\text{ID}_{\lambda_T=20}$ | 11 | 0 | 89 | 0 | 0 | $6 \times 10^{-3}$ | 0.11 | 11.7 |
| $\text{ID}_{\lambda_T=80}$ | 28 | 0 | 72 | 0 | 0 | $11 \times 10^{-3}$ | 0.29 | 5.8 |

**Table 12** Distribution of $\hat{N} - N$ over 100 simulated data sequences from the piecewise-constant signal (M5). The average MSE, $d_H$ and computational time are also given

| Method | $\hat{N} - N$: $\leq -500$ | $(-500, -50]$ | $(-50, -10)$ | $[-10, 10]$ | $> 10$ | MSE | $d_H$ | Time (s) |
|---|---|---|---|---|---|---|---|---|
| $\text{ID}_{\lambda_T=5}$ | 0 | 0 | 0 | 100 | 0 | 0.14 | 0.99 | 0.772 |
| $\text{ID}_{\lambda_T=20}$ | 100 | 0 | 0 | 0 | 0 | 1.28 | 3.45 | 0.395 |
| $\text{ID}_{\lambda_T=80}$ | 100 | 0 | 0 | 0 | 0 | 1.53 | 10.34 | 0.308 |

**Table 13** Distribution of $\hat{N} - N$ over 100 simulated data sequences from the continuous piecewise-linear signal (W1). The average MSE, $d_H$ and computational time for each method are also given

| Method | $\hat{N} - N$ | | | | | | | MSE | $d_H$ | Time (s) |
|---|---|---|---|---|---|---|---|---|---|---|
| | $\leq -3$ | $-2$ | $-1$ | 0 | 1 | 2 | $\geq 3$ | | | |
| $\text{ID}_{\lambda_T=5}$ | 0 | 0 | 0 | 91 | 9 | 0 | 0 | 0.031 | 0.104 | 0.036 |
| $\text{ID}_{\lambda_T=20}$ | 0 | 0 | 0 | 92 | 8 | 0 | 0 | 0.027 | 0.099 | 0.020 |
| $\text{ID}_{\lambda_T=80}$ | 0 | 0 | 0 | 99 | 1 | 0 | 0 | 0.020 | 0.067 | 0.017 |

**Table 14** Distribution of $\hat{N} - N$ over 100 simulated data sequences of the continuous piecewise-linear signal (W2). The average MSE, $d_H$ and computational time for each method are also given

| Method | $\hat{N} - N$ | | | | | | | MSE | $d_H$ | Time (s) |
|---|---|---|---|---|---|---|---|---|---|---|
| | $\leq -90$ | $(-90, -1)$ | $-1$ | 0 | 1 | $(1, 60]$ | $> 60$ | | | |
| $\text{ID}_{\lambda_T=5}$ | 0 | 0 | 0 | 97 | 3 | 0 | 0 | 0.227 | 0.272 | 0.690 |
| $\text{ID}_{\lambda_T=20}$ | 0 | 0 | 0 | 100 | 0 | 0 | 0 | 0.364 | 0.328 | 0.722 |
| $\text{ID}_{\lambda_T=80}$ | 100 | 0 | 0 | 0 | 0 | 0 | 0 | 4.730 | 98.955 | 0.102 |

**Table 15** Distribution of $\hat{N} - N$ over 100 simulated time series of the continuous piecewise-linear signals (W3) and (W4). The average MSE, $d_H$ and computational time are also given

| Method | Model | $\hat{N} - N$ | | | | | | | MSE | $d_H$ | Time (s) |
|---|---|---|---|---|---|---|---|---|---|---|---|
| | | $\leq -3$ | $-2$ | $-1$ | 0 | 1 | 2 | $\geq 3$ | | | |
| $\text{ID}_{\lambda_T=5}$ | | 0 | 0 | 0 | 94 | 6 | 0 | 0 | 0.020 | 0.122 | 0.014 |
| $\text{ID}_{\lambda_T=20}$ | (W3) | 0 | 0 | 0 | 99 | 1 | 0 | 0 | 0.009 | 0.067 | 0.012 |
| $\text{ID}_{\lambda_T=80}$ | | 100 | 0 | 0 | 0 | 0 | 0 | 0 | 3.917 | 1.875 | 0.009 |
| $\text{ID}_{\lambda_T=5}$ | | 0 | 0 | 0 | 77 | 22 | 1 | 0 | 0.055 | 0.134 | 0.040 |
| $\text{ID}_{\lambda_T=20}$ | (W4) | 0 | 0 | 0 | 98 | 2 | 0 | 0 | 0.029 | 0.106 | 0.032 |
| $\text{ID}_{\lambda_T=80}$ | | 0 | 36 | 50 | 14 | 0 | 0 | 0 | 1.603 | 0.950 | 0.030 |

In terms of accuracy, we notice that in most cases, the best method is $\text{ID}_{\lambda_T=5}$. In addition, as long as the different values of $\lambda_T$ are all less than the minimum distance, $\delta_T$, between two successive change-points, then the results are extremely similar (if not identical); see for example Tables 9 and 13. On the other hand, when $\lambda_T > \delta_T$ the accuracy is getting worse; compare for example the results for the three different values of $\lambda_T$ in Models (M2), (M3), (M5), (W2), and (W3). In terms of speed, as expected, the larger the value of $\lambda_T$, the quicker the method in general.

## 7 Additional real-data example

In this section we explore the behaviour of our method and two competitors, CPOP and NOT, to the daily closing stock prices of Samsung Electronics Co. from July

2012 until June 2020. The data are available from `https://finance.yahoo.com/quote/005930.KS/history?p=005930.KS` and they were accessed in July 2020. We look for changes in a continuous piecewise-linear mean signal. Figure 8 shows the results for the ID, ID.SDLL, NOT and CPOP methods, which detect $165, 134, 22, 239$ change-points, respectively. From both the fit and the residuals given in Figure 8, it is not easy to say which of the three methods gives the "best" number of change-points.

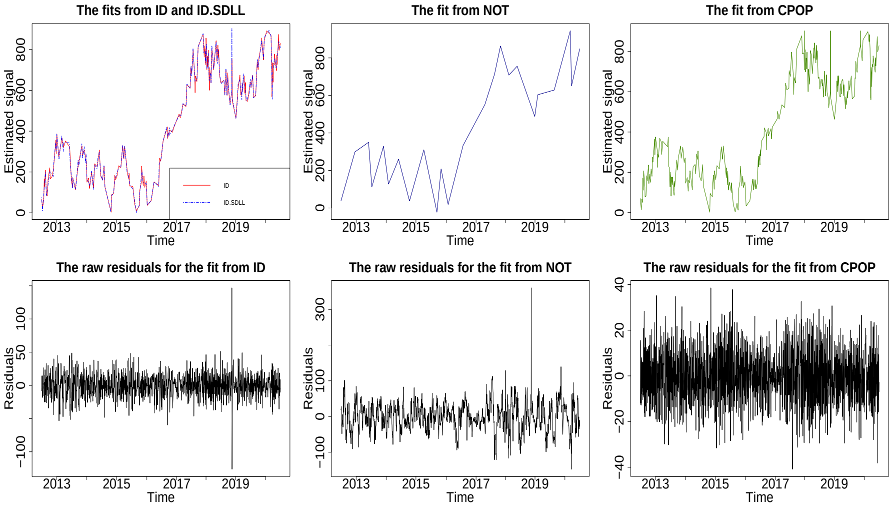


**Fig. 8** **Top row:** From left to right, the fits for ID and ID.SDLL (on the same plot), NOT and CPOP, respectively. **Bottom row:** The raw residuals $\epsilon_t = Y_t - \hat{f}_t$ for each method.

ID can return a range of different fits providing users with the flexibility to choose according to their preference. In Figure 9, we use the solution path and we obtain the estimated signal and the raw residuals of ID for $\hat{N} = 22$ and $\hat{N} = 239$, which are the estimated change-point numbers through NOT and CPOP, respectively. The fit is similar to those obtained by the aforementioned methods as presented in Figure 8. However, we note that both those competitors are significantly slower than ID; see Tables 6 and 7 in the main paper and Tables 6 - 8 in the supplement for a comparison. To conclude, apart from returning the estimated fit, the ID methodology can directly, and without any extra effort, produce a series of estimated signals based on the solution path defined in (10).

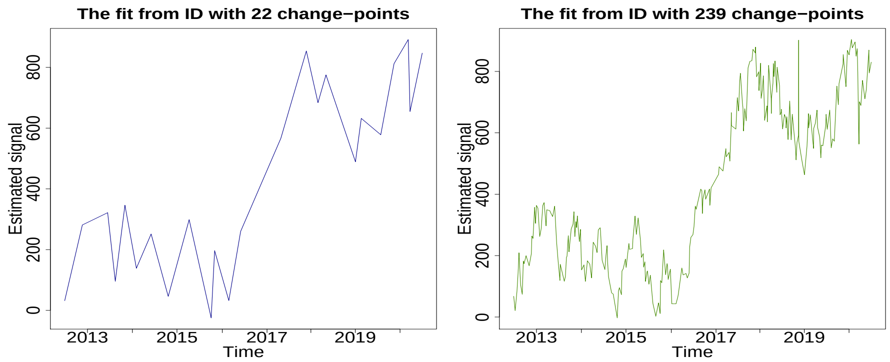


**Fig. 9** The estimated signals obtained by ID with 22 and 239 change-points. The solution path was employed in order to obtain these fits.

## 8 Proof of the theorems in the paper and of Corollary 1

From now on, the contrast vector $\boldsymbol{\psi_{s,e}^{b}} = (\psi_{s,e}^{b}(1), \psi_{s,e}^{b}(2), \ldots, \psi_{s,e}^{b}(T))$ is defined through the contrast function

$$\psi_{s,e}^{b}(t) = \begin{cases} \sqrt{\frac{e-b}{n(b-s+1)}}, & t = s, s+1, \ldots, b, \\ -\sqrt{\frac{b-s+1}{n(e-b)}}, & t = b+1, b+2, \ldots, e, \\ 0, & \text{otherwise.} \end{cases},$$

where $s \leq b < e$. Notice that for any vector $\boldsymbol{v} = (v_1, v_2, \ldots, v_T)$, we have that $\langle \boldsymbol{v}, \boldsymbol{\psi_{s,e}^{b}} \rangle = \tilde{v}_{s,e}^{b}$. We first present Lemma 1, which is partly used for the proof of Theorem 1,

**Lemma 1** *Suppose* $\boldsymbol{f} = (f_1, f_2, \ldots, f_T)^{\intercal}$ *is a piecewise-constant vector. Pick any interval* $[s, e] \subset [1, T]$ *such that* $[s, e-1]$ *contains exactly one change-point* $r_j$*. Let* $\rho = |r_j - b|$, $\Delta_j^f = \left|f_{r_j+1} - f_{r_j}\right|$, $\eta_L = r_j - s + 1$ *and* $\eta_R = e - r_j$*. Then,*

$$\|\psi_{s,e}^{b}\langle \boldsymbol{f}, \boldsymbol{\psi_{s,e}^{b}}\rangle - \boldsymbol{\psi_{s,e}^{r_j}}\langle \boldsymbol{f}, \boldsymbol{\psi_{s,e}^{r_j}}\rangle\|_2^2 = \left(\tilde{f}_{s,e}^{r_j}\right)^2 - \left(\tilde{f}_{s,e}^{b}\right)^2.$$

*In addition,*

1. *for any* $r_j \leq b < e$, $\left(\tilde{f}_{s,e}^{r_j}\right)^2 - \left(\tilde{f}_{s,e}^{b}\right)^2 = (\rho\eta_L/(\rho+\eta_L))\left(\Delta_j^f\right)^2$;
2. *for any* $s \leq b < r_j$, $\left(\tilde{f}_{s,e}^{r_j}\right)^2 - \left(\tilde{f}_{s,e}^{b}\right)^2 = (\rho\eta_R/(\rho+\eta_R))\left(\Delta_j^f\right)^2$;

*Proof* See Lemma 4 from Baranowski et al. (2019).

**Brief discussion of the steps of the proof of Theorem 1**

Before proceeding with the thorough mathematical proof, we give an informal explanation of the main steps. In the main part of the proof, we derive results for the signal $f_t$. However, the consistency is concerned with the estimated number and locations

of the change-points in the observed process $\{X_t\}_{t=1,2,\ldots,T}$. Therefore, in order to be able to deduce consistency related to $X_t$ from our $f_t$-reliant proof, we need first to show that for all $1 \leq s \leq b < e \leq T$, the observed quantity $\left|\tilde{X}_{s,e}^b\right|$ is uniformly close to the unobserved $\left|\tilde{f}_{s,e}^b\right|$; this is achieved in Step 1. In Step 2, for $b_1, b_2 \in [s,e)$, we control the distance between the noised $\left|\tilde{X}_{s,e}^{b_1}\right| - \left|\tilde{X}_{s,e}^{b_2}\right|$ and its noiseless equivalent $\left|\tilde{f}_{s,e}^{b_1}\right| - \left|\tilde{f}_{s,e}^{b_2}\right|$ for all possible combinations of $s, e, b_1, b_2$. This allows us to transfer the decision on whether $b_1$ or $b_2$ is more suitable as a change-point, from $\left|\tilde{f}_{s,e}^{b_1}\right| - \left|\tilde{f}_{s,e}^{b_2}\right|$ to the calculable $\left|\tilde{X}_{s,e}^{b_1}\right| - \left|\tilde{X}_{s,e}^{b_2}\right|$. Step 3 is the main part of our proof, where we first show that as the ID algorithm proceeds, each change-point will get isolated in an interval where its detection will occur with high probability. Therefore, it suffices to restrict our proof to a single change-point detection framework, and the convergence rate is proved to hold for each estimated location. Because upon detection ID proceeds from the end-point (or start-point) of the interval where the detection occurred, we also show that with probability one there is no change-point in those bypassed points (between the detection and the new start- or end-point). Furthermore, in Step 3 it is shown that, the new start- and end-points are at places that allow the detection of the next change-point. In Step 4, we conclude the proof by showing that after detecting all change-points, then ID, with high probability, will terminate after scanning all the remaining data. We mention that for our proof, we employ Lemma 1 given in the online supplement.

**Proof of Theorem 1**. We will prove the more specific result

$$\mathbb{P}\left(\hat{N} = N, \max_{j=1,2,\ldots,N}\left(|\hat{r}_j - r_j|\left(\Delta_j^f\right)^2\right) \leq C_3 \log T\right) \geq 1 - \frac{1}{6\sqrt{\pi}T}, \qquad (17)$$

which implies the result in (5).

**Step 1**: Allow us to denote by

$$A_T = \left\{\max_{s,b,e:1\leq s\leq b<e\leq T}\left|\tilde{X}_{s,e}^b - \tilde{f}_{s,e}^b\right| \leq \sqrt{8\log T}\right\}. \qquad (18)$$

We will show that $\mathbb{P}\,(A_T) \geq 1 - 1/(12\sqrt{\pi}T)$. From (3) and (4), simple steps yield $\tilde{X}_{s,e}^b - \tilde{f}_{s,e}^b = \tilde{\epsilon}_{s,e}^b$, where $\tilde{\epsilon}_{s,e}^b \sim \mathcal{N}(0,1)$. Thus, for $Z \sim \mathcal{N}(0,1)$, using the Bonferroni inequality we get that

$$\begin{aligned}
&\mathbb{P}\left((A_T)^c\right) = \mathbb{P}\left(\max_{s,b,e:1\leq s\leq b<e\leq T}\left|\tilde{X}_{s,e}^b - \tilde{f}_{s,e}^b\right| > \sqrt{8\log T}\right)\\
&\leq \sum_{1\leq s\leq b<e\leq T}\mathbb{P}\left(\left|\tilde{\epsilon}_{s,e}^b\right| > \sqrt{8\log T}\right) \leq \frac{T^3}{6}\mathbb{P}\left(|Z| > \sqrt{8\log T}\right)\\
&= \frac{T^3}{3}\mathbb{P}\left(Z > \sqrt{8\log T}\right) \leq \frac{T^3}{3}\frac{\phi(\sqrt{8\log T})}{\sqrt{8\log T}} \leq \frac{1}{12\sqrt{\pi}T},
\end{aligned}$$

where $\phi(\cdot)$ is the probability density function of the standard normal distribution.

**Step 2:** For intervals $[s, e)$ that contain only one true change-point $r_j$, we denote by

$$B_T = \left\{ \max_{j=1,2,\dots,N} \max_{\substack{r_{j-1}<s\leq r_j \\ r_j<e\leq r_{j+1} \\ s\leq b<e}} \frac{\left|\left\langle \boldsymbol{\psi}^{\boldsymbol{b}}_{\boldsymbol{s},\boldsymbol{e}}\langle \boldsymbol{f}, \boldsymbol{\psi}^{\boldsymbol{b}}_{\boldsymbol{s},\boldsymbol{e}}\rangle - \boldsymbol{\psi}^{\boldsymbol{r_j}}_{\boldsymbol{s},\boldsymbol{e}}\langle \boldsymbol{f}, \boldsymbol{\psi}^{\boldsymbol{r_j}}_{\boldsymbol{s},\boldsymbol{e}}\rangle, \boldsymbol{\epsilon}\right\rangle\right|}{\|\boldsymbol{\psi}^{\boldsymbol{b}}_{\boldsymbol{s},\boldsymbol{e}}\langle \boldsymbol{f}, \boldsymbol{\psi}^{\boldsymbol{b}}_{\boldsymbol{s},\boldsymbol{e}}\rangle - \boldsymbol{\psi}^{\boldsymbol{r_j}}_{\boldsymbol{s},\boldsymbol{e}}\langle \boldsymbol{f}, \boldsymbol{\psi}^{\boldsymbol{r_j}}_{\boldsymbol{s},\boldsymbol{e}}\rangle\|_2} \leq \sqrt{8\log T} \right\}. \tag{19}$$

Because $\left|\left\langle \boldsymbol{\psi}^{\boldsymbol{b}}_{\boldsymbol{s},\boldsymbol{e}}\langle \boldsymbol{f}, \boldsymbol{\psi}^{\boldsymbol{b}}_{\boldsymbol{s},\boldsymbol{e}}\rangle - \boldsymbol{\psi}^{\boldsymbol{r_j}}_{\boldsymbol{s},\boldsymbol{e}}\langle \boldsymbol{f}, \boldsymbol{\psi}^{\boldsymbol{r_j}}_{\boldsymbol{s},\boldsymbol{e}}\rangle\right\rangle\right| / \left(\|\boldsymbol{\psi}^{\boldsymbol{b}}_{\boldsymbol{s},\boldsymbol{e}}\langle \boldsymbol{f}, \boldsymbol{\psi}^{\boldsymbol{b}}_{\boldsymbol{s},\boldsymbol{e}}\rangle - \boldsymbol{\psi}^{\boldsymbol{r_j}}_{\boldsymbol{s},\boldsymbol{e}}\langle \boldsymbol{f}, \boldsymbol{\psi}^{\boldsymbol{r_j}}_{\boldsymbol{s},\boldsymbol{e}}\rangle\|_2\right)$ follows the standard normal distribution, then we use a similar approach as in Step 1, to show that $\mathbb{P}\left((B_T)^c\right) \leq \frac{1}{12\sqrt{\pi}T}$. Therefore, Steps 1 and 2 lead to

$$\mathbb{P}\left(A_T \cap B_T\right) \geq 1 - \frac{1}{6\sqrt{\pi}T}.$$

**Step 3:** This is the main part of our proof, where we explain in detail how to get the result in (17). For ease of understanding, we split this step into two smaller parts. From now on, we assume that $A_T$ and $B_T$ both hold. The constants we use are

$$C_1 = \sqrt{C_3} + \sqrt{8}, C_2 = \frac{1}{\sqrt{6}} - \frac{2\sqrt{2}}{\underline{C}}, C_3 = 2(2\sqrt{2}+4)^2, \tag{20}$$

where $\underline{C}$ is as in condition (A1).

**Step 3.1:** For ease of presentation, we take $\lambda_T \leq \delta_T/3$; see Remark 1 for comments in regards to the general case of $\lambda_T \leq \delta_T/m$, for an $m > 1$. Allow us now $\forall j \in \{1, 2, \dots, N\}$, to define the intervals

$$I_j^R = \left[r_j + \frac{\delta_T}{3}, r_j + 2\frac{\delta_T}{3}\right), \qquad I_j^L = \left(r_j - 2\frac{\delta_T}{3}, r_j - \frac{\delta_T}{3}\right]. \tag{21}$$

In order for $I_j^R$ and $I_j^L$ to have at least one point, we actually implicitly require that $\delta_T > 3$, which is the case for sufficiently large $T$; see assumption (A1). Since the length of the intervals in (21) is equal to $\delta_T/3$ and $\lambda_T \leq \delta_T/3$, then ID ensures that for $K = \lceil T/\lambda_T \rceil$ and $k, m \in \{1, 2, \dots, K\}$, there exists at least one $c_k^r = k\lambda_T$ and at least one $c_m^l = T - m\lambda_T + 1$ that are in $I_j^R$ and $I_j^L$, $\forall j = 1, 2, \dots, N$. At the beginning of our algorithm, $s = 1$, $e = T$ and depending on whether $r_1 \leq T - r_N$ then $r_1$ or $r_N$ will get isolated in a right- or left-expanding interval, respectively. W.l.o.g., assume that $r_1 \leq T - r_N$. As already mentioned, ID naturally ensures that $\exists k \in \{1, 2, \dots, K\}$ such that $c_k^r \in I_1^R$. There is no other change-point in $[1, c_k^r]$ apart from $r_1$. We will show that for $\tilde{b} = \text{argmax}_{1\leq t<c_k^r}\left|\tilde{X}^t_{1,c_k^r}\right|$, then $\left|\tilde{X}^{\tilde{b}}_{1,c_k^r}\right| > \zeta_T$. Using (18), we have that

$$\left|\tilde{X}^{\tilde{b}}_{1,c_k^r}\right| \geq \left|\tilde{X}^{r_1}_{1,c_k^r}\right| \geq \left|\tilde{f}^{r_1}_{1,c_k^r}\right| - \sqrt{8\log T}. \tag{22}$$

But,

$$\left|\tilde{f}^{r_1}_{1,c^r_{\tilde{k}}}\right| = \left|\sqrt{\frac{c^r_{\tilde{k}} - r_1}{r_1 c^r_{\tilde{k}}}} r_1 f_{r_1} - \sqrt{\frac{r_1}{c^r_{\tilde{k}}(c^r_{\tilde{k}} - r_1)}}(c^r_{\tilde{k}} - r_1) f_{r_1+1}\right| = \sqrt{\frac{(c^r_{\tilde{k}} - r_1)r_1}{c^r_{\tilde{k}}}} \Delta^f_1$$
$$= \sqrt{\frac{(c^r_{\tilde{k}} - r_1)r_1}{(c^r_{\tilde{k}} - r_1) + r_1}} \Delta^f_1 \geq \sqrt{\frac{(c^r_{\tilde{k}} - r_1)r_1}{2\max\{c^r_{\tilde{k}} - r_1, r_1\}}} \Delta^f_1 = \sqrt{\frac{\min\{c^r_{\tilde{k}} - r_1, r_1\}}{2}} \Delta^f_1. \tag{23}$$

By the definition of $\delta_T$ and from our notation of $r_0 = 0$, we know that $r_1 \geq \delta_T$. In addition, since $c^r_{\tilde{k}} \in I^R_1$, then $\delta_T/3 \leq c^r_{\tilde{k}} - r_1 < 2\delta_T/3$, meaning that

$$\min\{c^r_{\tilde{k}} - r_1, r_1\} \geq \frac{\delta_T}{3}. \tag{24}$$

The result in (22), the assumption (A1) and the application of (24) in (23) yield

$$\left|\tilde{X}^{\tilde{b}}_{1,c^r_{\tilde{k}}}\right| \geq \sqrt{\frac{\delta_T}{6}} \Delta^f_1 - \sqrt{8\log T} \geq \sqrt{\frac{\delta_T}{6}} \underline{f}_T - \sqrt{8\log T}$$
$$= \left(\frac{1}{\sqrt{6}} - \frac{2\sqrt{2\log T}}{\sqrt{\delta_T}\underline{f}_T}\right) \sqrt{\delta_T}\underline{f}_T \geq \left(\frac{1}{\sqrt{6}} - \frac{2\sqrt{2}}{\underline{C}}\right) \sqrt{\delta_T}\underline{f}_T$$
$$= C_2\sqrt{\delta_T}\underline{f}_T > \zeta_T.$$

Therefore, there will be an interval of the form $[1, c^r_{\tilde{k}}]$, with $c^r_{\tilde{k}} > r_1$, such that $[1, c^r_{\tilde{k}}]$ contains only $r_1$ and $\max_{1\leq b<c^r_{\tilde{k}}} \left|\tilde{X}^b_{1,c^r_{\tilde{k}}}\right| > \zeta_T$. Let us, for $k^* \in \{1, 2, \ldots, K\}$, to denote by $c^r_{k^*} \leq c^r_{\tilde{k}}$ the first right-expanding point where this happens and let $b_1 = \mathrm{argmax}_{1\leq t<c^r_{k^*}} \left|\tilde{X}^t_{1,c^r_{k^*}}\right|$ with $\left|\tilde{X}^{b_1}_{1,c^r_{k^*}}\right| > \zeta_T$. Our aim now is to find $\gamma_T > 0$ such that for any $b^* \in \{1, 2, \ldots, c^r_{k^*} - 1\}$ with $|b^* - r_1| \left(\Delta^f_1\right)^2 > \gamma_T$, we have that

$$\left(\tilde{X}^{r_1}_{1,c^r_{k^*}}\right)^2 > \left(\tilde{X}^{b^*}_{1,c^r_{k^*}}\right)^2. \tag{25}$$

Proving (25) and using the definition of $b_1$ we can conclude that $|b_1 - r_1| \left(\Delta^f_1\right)^2 \leq \gamma_T$. Now, since $X_t = f_t + \epsilon_t$, then (25) can be expressed as

$$\left(\tilde{f}^{r_1}_{1,c^r_{k^*}}\right)^2 - \left(\tilde{f}^{b^*}_{1,c^r_{k^*}}\right)^2 > \left(\tilde{\epsilon}^{b^*}_{1,c^r_{k^*}}\right)^2 - \left(\tilde{\epsilon}^{r_1}_{1,c^r_{k^*}}\right)^2$$
$$+ 2\left\langle \boldsymbol{\psi}^{\boldsymbol{b^*}}_{\boldsymbol{1,c^r_{k^*}}} \langle \boldsymbol{f}, \boldsymbol{\psi}^{\boldsymbol{b^*}}_{\boldsymbol{1,c^r_{k^*}}} \rangle - \boldsymbol{\psi}^{\boldsymbol{r_1}}_{\boldsymbol{1,c^r_{k^*}}} \langle \boldsymbol{f}, \boldsymbol{\psi}^{\boldsymbol{r_1}}_{\boldsymbol{1,c^r_{k^*}}} \rangle, \boldsymbol{\epsilon} \right\rangle. \tag{26}$$

W.l.o.g. assume that $b^* \geq r_1$ and a similar approach as below holds when $b^* < r_1$. Lemma 1, gives for the left-hand side of the inequality in (26) that

$$\left(\tilde{f}^{r_1}_{1,c^r_{k^*}}\right)^2 - \left(\tilde{f}^{b^*}_{1,c^r_{k^*}}\right)^2 = \frac{|b^* - r_1|\, r_1}{|b^* - r_1| + r_1} \left(\Delta^f_1\right)^2 := \Lambda. \tag{27}$$

For the terms on the right-hand side of (26), using (18) we obtain that

$$\left(\tilde{\epsilon}^{b^*}_{1,c^r_{k^*}}\right)^2 - \left(\tilde{\epsilon}^{r_1}_{1,c^r_{k^*}}\right)^2 \leq \max_{s,e,b:s\leq b<e}\left(\tilde{\epsilon}^{b}_{s,e}\right)^2 - \left(\tilde{\epsilon}^{r_1}_{1,c^r_{k^*}}\right)^2 \leq \max_{s,e,b:s\leq b<e}\left(\tilde{\epsilon}^{b}_{s,e}\right)^2 \leq 8\log T,$$

while from (19) and Lemma 1,

$$\begin{aligned}&2\left\langle \boldsymbol{\psi}^{\boldsymbol{b^*}}_{\boldsymbol{1,c^r_{k^*}}}\langle \boldsymbol{f}, \boldsymbol{\psi}^{\boldsymbol{b^*}}_{\boldsymbol{1,c^r_{k^*}}}\rangle - \boldsymbol{\psi}^{\boldsymbol{r_1}}_{\boldsymbol{1,c^r_{k^*}}}\langle \boldsymbol{f}, \boldsymbol{\psi}^{\boldsymbol{r_1}}_{\boldsymbol{1,c^r_{k^*}}}\rangle, \boldsymbol{\epsilon}\right\rangle \\ &\leq 2\|\boldsymbol{\psi}^{\boldsymbol{b^*}}_{\boldsymbol{1,c^r_{k^*}}} < \boldsymbol{f}, \boldsymbol{\psi}^{\boldsymbol{b^*}}_{\boldsymbol{1,c^r_{k^*}}} > -\boldsymbol{\psi}^{\boldsymbol{r_1}}_{\boldsymbol{1,c^r_{k^*}}} < \boldsymbol{f}, \boldsymbol{\psi}^{\boldsymbol{r_1}}_{\boldsymbol{1,c^r_{k^*}}} > \|_2\sqrt{8\log T} = 2\sqrt{\Lambda}\sqrt{8\log T}.\end{aligned}$$

Therefore (26) is satisfied if the stronger inequality $\Lambda > 8\log T + 2\sqrt{\Lambda}\sqrt{8\log T}$ is satisfied, which has solution

$$\Lambda > (2\sqrt{2}+4)^2\log T.$$

From (27) and since $(|b^* - r_1|r_1)/(|b^* - r_1| + r_1) \geq \min\{|b^* - r_1|, r_1\}/2$, we deduce that (25) is implied by

$$\min\{|b^* - r_1|, r_1\} > \frac{2(2\sqrt{2}+4)^2\log T}{\left(\Delta^f_1\right)^2} = \frac{C_3\log T}{\left(\Delta^f_1\right)^2}. \tag{28}$$

However,

$$\min\{r_1, c^r_{k^*} - r_1\} > C_3\frac{\log T}{\left(\Delta^f_1\right)^2} \tag{29}$$

and this is because if we assume that $\min\{r_1, c^r_{k^*} - r_1\} \leq C_3\log T/\left(\Delta^f_1\right)^2$, then

$$\begin{aligned}\left|\tilde{X}^{b_1}_{1,c^r_{k^*}}\right| &\leq \left|\tilde{f}^{r_1}_{1,c^r_{k^*}}\right| + \sqrt{8\log T} = \sqrt{\frac{(c^r_{k^*} - r_1)r_1}{c^r_{k^*}}}\Delta^f_1 + \sqrt{8\log T} \\ &\leq \sqrt{\min\{c^r_{k^*} - r_1, r_1\}}\Delta^f_1 + \sqrt{8\log T} \leq \left(\sqrt{C_3} + \sqrt{8}\right)\sqrt{\log T} \\ &= C_1\sqrt{\log T} \leq \zeta_T.\end{aligned}$$

This comes to a contradiction to $\left|\tilde{X}^{b_1}_{1,c^r_{k^*}}\right| > \zeta_T$. Therefore, (29) holds and (28) is restricted to $|b^* - r_1|\left(\Delta^f_1\right)^2 > C_3\log T$, which implies (25). Thus, we conclude that necessarily,

$$|b_1 - r_1|\left(\Delta^f_1\right)^2 \leq C_3\log T. \tag{30}$$

So far, for $\lambda_T \leq \delta_T/3$ we have proven that working under the assumption that $A_T$ and $B_T$ hold, there will be an interval $[1, c^r_{k^*}]$, with $\left|\tilde{X}^{b_1}_{1,c^r_{k^*}}\right| > \zeta_T$, where $b_1 = \underset{1\leq t<c^r_{k^*}}{\operatorname{argmax}}\left|\tilde{X}^{t}_{1,c^r_{k^*}}\right|$ is an estimation of $r_1$ that satisfies (30).

**Step 3.2:** After detecting the first change-point, ID follows the same process as in Step 3.1 but in the set $[c_{k^*}^r, T]$, which contains $r_2, r_3, \ldots, r_N$. This means that we bypass, without checking for possible change-points, the interval $[b_1+1, c_{k^*}^r)$ and we need to prove that:

(S.1) There is no change-point in $[b_1+1, c_{k^*}^r)$, apart from maybe the already detected $r_1$;
(S.2) $c_{k^*}^r$ is at a location which allows for detection of $r_2$.

**For (S.1):** We will split the explanation into two cases with respect to the location of $b_1$.
**Case 1:** $b_1 < r_1 < c_{k^*}^r$. Using (30) and imposing the condition

$$\delta_T > 3C_3 \frac{\log T}{\left(\Delta_1^f\right)^2}, \tag{31}$$

then since $c_{\tilde{k}}^r \in I_1^R$, we have that

$$c_{k^*}^r - b_1 \leq c_{\tilde{k}}^r - b_1 = c_{\tilde{k}}^r - r_1 + r_1 - b_1 < 2\frac{\delta_T}{3} + r_1 - b_1 \leq 2\frac{\delta_T}{3} + \frac{C_3 \log T}{\left(\Delta_1^f\right)^2} < \delta_T.$$

Since $r_2 - r_1 \geq \delta_T$ and $r_1$ is already in $[b_1+1, c_{k^*}^r)$, then there is no other change-point in $[b_1+1, c_{k^*}^r)$ apart from $r_1$. Actually, the result in (31) is not an extra assumption and we will briefly explain the reason at the end of our proof.
**Case 2:** $r_1 \leq b_1 < c_{k^*}^r$. Since $c_{\tilde{k}}^r \in I_1^R$, then $c_{k^*}^r - r_1 \leq c_{\tilde{k}}^r - r_1 < 2\delta_T/3$, which means that apart from $r_1$ there is no other change-point in $[r_1, c_{k^*}^r)$. With $r_1 \leq b_1$, then $[b_1+1, c_{k^*}^r)$ does not have any change-point.

Cases 1 and 2 above show that no matter the location of $b_1$, there is no change-point in $[b_1+1, c_{k^*}^r)$ other than possibly the previously detected $r_1$. Similarly to the approach in Step 3.1, our method applied now in $[c_{k^*}^r, T]$, will first isolate $r_2$ or $r_N$ depending on whether $r_2 - c_{k^*}^r$ is smaller or larger than $T - r_N$. If $T - r_N < r_2 - c_{k^*}^r$ then $r_N$ will get isolated first in a left-expanding interval and the procedure to show its detection is exactly the same as for the detection of $r_1$ in Step 3.1. Therefore, for the sake of showing (S.2) let us assume that $r_2 - c_{k^*}^r \leq T - r_N$.

**For (S.2):** With $\mathrm{R}_{s,e}$ as in (2), there exists $c_{k_2}^r \in \mathrm{R}_{c_{k^*}^r, T}$ such that $c_{k_2}^r \in I_2^R$, with $I_j^R$ defined in (21). We will show that $r_2$ gets detected in $[c_{k^*}^r, c_{k_2^*}^r]$, for $k_2^* \leq k_2$ and its detection is $b_2 = \mathrm{argmax}_{c_{k^*}^r \leq t < c_{k_2^*}^r} \left|\tilde{X}_{c_{k^*}^r, c_{k_2^*}^r}^t\right|$, which satisfies $|b_2 - r_2|\left(\Delta_2^f\right)^2 \leq C_3 \log T$. Following similar steps as in (23), we have that for $\tilde{b}_2 = \underset{c_{k^*}^r \leq t < c_{k_2}^r}{\mathrm{argmax}} \left|\tilde{X}_{c_{k^*}^r, c_{k_2}^r}^t\right|$,

$$\left|\tilde{X}_{c_{k^*}^r, c_{k_2}^r}^{\tilde{b}_2}\right| \geq \left|\tilde{f}_{c_{k^*}^r, c_{k_2}^r}^{r_2}\right| - \sqrt{8\log T} \geq \sqrt{\frac{\min\left\{c_{k_2}^r - r_2, r_2 - c_{k^*}^r + 1\right\}}{2}}\Delta_2^f - \sqrt{8 \log T}. \tag{32}$$

By construction, $c^r_{\tilde{k}_2} - r_2 \geq \delta_T/3$ and

$$r_2 - c^r_{k^*} + 1 \geq r_2 - c^r_{\tilde{k}} + 1 = r_2 - r_1 - (c^r_{\tilde{k}} - r_1) + 1 \geq \delta_T - (c^r_{\tilde{k}} - r_1) + 1$$
$$> \delta_T - 2\frac{\delta_T}{3} + 1 > \frac{\delta_T}{3},$$

which means that $\min\left\{c^r_{\tilde{k}_2} - r_2, r_2 - c^r_{k^*} + 1\right\} \geq (\delta_T/3)$ and therefore continuing from (32),

$$\left|\tilde{X}^{\tilde{b}_2}_{c^r_{k^*}, c^r_{\tilde{k}_2}}\right| \geq \sqrt{\frac{\delta_T}{6}}\Delta^f_2 - \sqrt{8\log T} \geq \left(\frac{1}{\sqrt{6}} - \frac{2\sqrt{2}\sqrt{\log T}}{\sqrt{\delta_T}\underline{f}_T}\right)\sqrt{\delta_T}\underline{f}_T$$
$$\geq \left(\frac{1}{\sqrt{6}} - \frac{2\sqrt{2}}{\underline{C}}\right)\sqrt{\delta_T}\underline{f}_T = C_2\sqrt{\delta_T}\underline{f}_T > \zeta_T.$$

Therefore, for a $c^r_{\tilde{k}_2} \in \mathrm{R}_{c^r_{k^*}, T}$ we have shown that there exists an interval of the form $[c^r_{k^*}, c^r_{\tilde{k}_2}]$, with $\max_{c^r_{k^*} \leq b < c^r_{\tilde{k}_2}} \left|\tilde{X}^b_{c^r_{k^*}, c^r_{\tilde{k}_2}}\right| > \zeta_T$. Let us denote by $c^r_{k^*_2} \in \mathrm{R}_{c^r_{k^*}, T}$ the first right-expanding point where this occurs and let $b_2 = \mathrm{argmax}_{c^r_{k^*} \leq t < c^r_{k^*_2}} \left|\tilde{X}^t_{c^r_{k^*}, c^r_{k^*_2}}\right|$ with $\left|\tilde{X}^{b_2}_{c^r_{k^*}, c^r_{k^*_2}}\right| > \zeta_T$.

We will now show that $|b_2 - r_2|\left(\Delta^f_2\right)^2 \leq C_3 \log T$. Following exactly the same process as in Step 3.1 and assuming now w.l.o.g. that $b_2 < r_2$, we have that for $b^* \in \left\{c^r_{k^*}, \ldots, c^r_{k^*_2} - 1\right\}$,

$$\left(\tilde{X}^{r_2}_{c^r_{k^*}, c^r_{k^*_2}}\right)^2 > \left(\tilde{X}^{b^*}_{c^r_{k^*}, c^r_{k^*_2}}\right)^2 \tag{33}$$

is implied by $\min\left\{|b^* - r_2|, c^r_{k^*_2} - r_2\right\} > C_3 \log T / \left(\Delta^f_2\right)^2$. In the same way as in Step 3.1 and by contradiction we can show that $\min\left\{c^r_{k^*_2} - r_2, r_2 - c^r_{k^*} + 1\right\} > C_3 \log T / \left(\Delta^f_2\right)^2$ and (33) is implied by $|b^* - r_2|\left(\Delta^f_2\right)^2 > C_3 \log T$. Therefore $|b_2 - r_2|\left(\Delta^f_2\right)^2 > C_3 \log T$ would mean that $\left|\tilde{X}^{r_2}_{c^r_{k^*}, c^r_{k^*_2}}\right| > \left|\tilde{X}^{b_2}_{c^r_{k^*}, c^r_{k^*_2}}\right|$, which is not true by the definition of $b_2$. Having said this, we conclude that $|b_2 - r_2|\left(\Delta^f_2\right)^2 \leq C_3 \log T$. Having detected $r_2$, then our algorithm will proceed in the interval $[s, e] = [c^r_{k^*_2}, T]$ and all the change-points will get detected one by one since Step 3.2 will be applicable as long as there are undetected change-points in $[s, e]$.

Denoting by $\hat{r}_j$ the estimation of $r_j$ as we did in the statement of the theorem, then we conclude that all change-points will get detected one by one and $|\hat{r}_j - r_j|\left(\Delta^f_j\right)^2 \leq C_3 \log T, \quad \forall j \in \{1, 2, \ldots, N\}$. In addition, as one can see from (31), our process

imposes that $\delta_T > 3C_3 \log T / \left(\Delta_j^f\right)^2, \forall j \in \{1, 2, \ldots, N\}$, which, by the definition of $\underline{f}_T$, is implied by

$$\delta_T > 3\frac{C_3 \log T}{\underline{f}_T^2}. \tag{34}$$

We will now explain why (34) is not actually an extra assumption but it is implied by our assumption (A1), which requires $\delta_T \geq \underline{C}^2 \log T/\underline{f}_T^2$. Proving that $\underline{C} > \sqrt{3C_3}$ would mean that indeed (A1) implies (34). Due to $C_1\sqrt{\log T} \leq \zeta_T < C_2\sqrt{\delta_T}\underline{f}_T$, we require $\underline{C}$ to be such that $\underline{C}C_2 > C_1$. Simple steps yield

$$\underline{C}C_2 > C_1 \Leftrightarrow \underline{C}\left(\frac{1}{\sqrt{6}} - \frac{2\sqrt{2}}{\underline{C}}\right) > \sqrt{C_3}+\sqrt{8} \Leftrightarrow \underline{C} > \sqrt{6}\left(\sqrt{C_3} + 4\sqrt{2}\right). \tag{35}$$

We conclude that $\underline{C} > \sqrt{3C_3}$, meaning that (34) is something already satisfied due to (A1).

**Step 4:** The arguments given in Steps 1-3 hold in $A_T \cap B_T$. At the beginning of the algorithm, $s = 1, e = T$ and for $N \geq 1$, there exist $k_1 \in \{1, 2, \ldots, K\}$ such that $s_{k_1} = s, e_{k_1} \in I_1^R$ and $k_2 \in \{1, 2, \ldots, K\}$ such that $s_{k_2} \in I_N^L, e_{k_2} = e$. As in our previous steps, w.l.o.g. assume that $r_1 \leq T - r_N$ and $r_1$ gets isolated and detected first in an interval $[s, c_{k^*}^r]$, where $c_{k^*}^r \in \mathrm{R}_{1,T}$ and it is less than or equal to $e_{k_1}$. Then, $\hat{r}_1 = \mathrm{argmax}_{s \leq t < c_{k^*}^r} |\tilde{X}_{s,c_{k^*}^r}^t|$ is the estimated location for $r_1$ and $|r_1 - \hat{r}_1| \left(\Delta_1^f\right)^2 \leq C_3 \log T$. After this, the method continues in $[c_{k^*}^r, T]$ and keeps detecting all the change-points as explained in Step 3. There will not be any double detection issues because naturally, at each step of the algorithm, the new interval $[s, e]$ does not include any previously detected change-points. Once all the change-points have been detected one by one, then $[s, e]$ will contain no other change-points. ID will keep checking for possible change-points in intervals of the form $\left[s, c_{\tilde{k}_1}^r\right]$ and $\left[c_{\tilde{k}_2}^l, e\right]$ for $c_{\tilde{k}_1}^r \in \mathrm{R}_{s,e}$ and $c_{\tilde{k}_2}^l \in \mathrm{L}_{s,e}$. We denote by $[s^*, e^*]$ any of these intervals. ID will not detect anything in $[s^*, e^*]$ since $\forall b \in [s^*, e^*)$,

$$\left|\tilde{X}_{s^*,e^*}^b\right| \leq \left|\tilde{f}_{s^*,e^*}^b\right| + \sqrt{8\log T} = \sqrt{8\log T} < C_1\sqrt{\log T} \leq \zeta_T.$$

After not detecting anything in all intervals of the above form, then the algorithm concludes that there are not any change-points in $[s, e]$ and stops. ■

*Remark 1* It is interesting to explore what happens when instead of $\lambda_T \leq \delta_T/3$, we use the more general case of $\lambda_T \leq \delta_T/m$, for $m > 1$. The adjustments need to be made are

(Adj.1) Instead of the definition in (21), we now have

$$I_j^R = \left[r_j + \frac{(m-1)\delta_T}{2m}, r_j + \frac{(m+1)\delta_T}{2m}\right)$$
$$I_j^L = \left(r_j - \frac{(m+1)\delta_T}{2m}, r_j - \frac{(m-1)\delta_T}{2m}\right].$$

Note that the length of the above intervals is $\delta_T/m$, meaning that with probability one there will be at least one left and one right expanding point in each of them because the distance between two consecutive right (left) expanding points is $\lambda_T \leq \delta_T/m$.

(Adj.2) Instead of $C_2$ as in (20), we should now use that $C_2 = \sqrt{(m-1)/4m} - 2\sqrt{2}/\underline{C}$. This is easy to prove and it will not be shown here.

(Adj.3) In (35), we give a lower bound for $\underline{C}$. Following similar steps, this now becomes

$$\underline{C} > \sqrt{\frac{4m}{m-1}}\left(\sqrt{C_3} + 4\sqrt{2}\right).$$

We see from (Adj.3) that the higher the value of $m$, the smaller the lower bound will be, meaning that the assumption on $\underline{C}$ gets possibly relaxed for larger values of $m$. On the other hand, the results above hold for an expanding level of $\lambda_T \leq \delta_T/m$ and thus, we notice that the smaller the value of $m$, the larger the upper bound for the acceptable $\lambda_T$-values. Our choice of $m = 3$ gives a more symmetric aspect to our approach as the length of the intervals $I_j^R$ and $I_j^L$ is the same as the minimum distance of their start- and end-points from possible change-points, which is $\delta_T/3$.

We now proceed to prove the result in Theorem 2. For the continuous piecewise-linear case, the contrast function values at $b$ for the observed data, the signal, and the noise are denoted by $C_{s,e}^b(\boldsymbol{X})$, $C_{s,e}^b(\boldsymbol{f})$ and $C_{s,e}^b(\boldsymbol{\epsilon})$, respectively. We have $\Delta_j^f = \left|2f_{r_j} - f_{r_j-1} - f_{r_j+1}\right|$ and as in the case of piecewise-constancy, $\underline{f}_T = \min_{j=1,2,\dots,N} \Delta_j^f$. The contrast vector $\boldsymbol{\phi_{s,e}^b} = (\phi_{s,e}^b(1), \phi_{s,e}^b(2), \dots, \phi_{s,e}^b(T))$ is defined through the contrast function

$$\phi_{s,e}^b(t) = \begin{cases} \alpha_{s,e}^b\beta_{s,e}^b\left[(e+2b-3s+2)t - (be+bs-2s^2+2s)\right], & t = s,\dots,b, \\ -\frac{\alpha_{s,e}^b}{\beta_{s,e}^b}\left[(3e-2b-s+2)t - (2e^2+2e-be-bs)\right], & t = b+1,\dots,e, \\ 0, & \text{otherwise}, \end{cases}$$

where $\alpha_{s,e}^b = (6/[n(n^2-1)(1+(e-b+1)(b-s+1)+(e-b)(b-s))])^{1/2}$ and $\beta_{s,e}^b = ([(e-b+1)(e-b)]/[(b-s+1)(b-s)])^{1/2}$, with $n = e-s+1$. For any vector $\boldsymbol{v} = (v_1, v_2, \dots, v_T)$, we have that

$$\left|\langle \boldsymbol{v}, \boldsymbol{\phi_{s,e}^b}\rangle\right| = C_{s,e}^b(\boldsymbol{v}).$$

Towards the proof of Theorem 2, we use Lemmas 2 and 3 given below.

**Lemma 2** *Suppose $\boldsymbol{f} = (f_1, f_2, \dots, f_T)^\intercal$ is piecewise-linear vector and $r_1, \dots, r_N$ are the locations of the change-points. Suppose $1 \leq s < e \leq T$, such that $r_{j-1} \leq s < r_j < e \leq r_{j+1}$, for some $j = 1, 2, \dots, N$. Let $\eta = \min\{r_j - s, e - r_j\}$. Then,*

$$C_{s,e}^{r_j}(\boldsymbol{f}) = \max_{s<b<e} C_{s,e}^b(\boldsymbol{f}) \begin{cases} \geq \frac{1}{\sqrt{24}}\eta^{\frac{3}{2}}\Delta_j^f, \\ \leq \frac{1}{\sqrt{3}}(\eta+1)^{\frac{3}{2}}\Delta_j^f, \end{cases}.$$

*Proof* See Lemma 5 from Baranowski et al. (2019).

**Lemma 3** *Suppose* $\boldsymbol{f} = (f_1, f_2, \ldots, f_T)^\intercal$ *is piecewise-linear vector. With* $r_1, r_2, \ldots, r_N$ *the locations of the change-points, suppose that* $1 \leq s < e \leq T$*, such that* $r_{j-1} \leq s < r_j < e \leq r_{j+1}$ *for some* $j = 1, 2, \ldots, N$*. Let* $\rho = |r_j - b|$*,* $\Delta_j^f = \left|2f_{r_j} - f_{r_j-1} - f_{r_j+1}\right|$*,* $\eta_L = r_j - s$ *and* $\eta_R = e - r_j$*. Then,*

$$\|\phi_{s,e}^b\langle \boldsymbol{f}, \boldsymbol{\phi_{s,e}^b}\rangle - \boldsymbol{\phi_{s,e}^{r_j}}\langle \boldsymbol{f}, \boldsymbol{\phi_{s,e}^{r_j}}\rangle\|_2^2 = \left(C_{s,e}^{r_j}(\boldsymbol{f})\right)^2 - \left(C_{s,e}^b(\boldsymbol{f})\right)^2.$$

*Furthermore,*

1. *for any* $r_j \leq b < e$*,* $\left(C_{s,e}^{r_j}(\boldsymbol{f})\right)^2 - \left(C_{s,e}^b(\boldsymbol{f})\right)^2 \geq (1/63)\min(\rho, \eta_L)^3\left(\Delta_j^f\right)^2$*;*
2. *for any* $s < b \leq r_j$*,* $\left(C_{s,e}^{r_j}(\boldsymbol{f})\right)^2 - \left(C_{s,e}^b(\boldsymbol{f})\right)^2 \geq (1/63)\min(\rho, \eta_R)^3\left(\Delta_j^f\right)^2$*.*

*Proof* See Lemma 7 from Baranowski et al. (2019), where the approach is similar as to the one for Lemma 1.

The steps we follow for the proof of Theorem 2 are the same as those explained for the proof of Theorem 1.

**Proof of Theorem 2**. We will prove the more specific result

$$\mathbb{P}\left(\hat{N} = N, \max_{j=1,2,\ldots,N}\left(|\hat{r}_j - r_j|\left(\Delta_j^f\right)^{\frac{2}{3}}\right) \leq C_3(\log T)^{\frac{1}{3}}\right) \leq 1 - \frac{1}{6\sqrt{\pi}T}, \quad (36)$$

which implies the result in (8).
**Steps 1 and 2**: As in Theorem 1, let

$$A_T^* = \left\{\max_{s,b,e:1\leq s\leq b<e\leq T}\left|C_{s,e}^b(\boldsymbol{X}) - C_{s,e}^b(\boldsymbol{f})\right| \leq \sqrt{8\log T}\right\}.$$

$$B_T^* = \left\{\max_{j=1,2,\ldots,N}\max_{\substack{r_{j-1}<s\leq r_j\\ r_j<e\leq r_{j+1}\\ s\leq b<e}}\frac{\left|\langle\boldsymbol{\phi_{s,e}^b}\langle\boldsymbol{f}, \boldsymbol{\phi_{s,e}^b}\rangle - \boldsymbol{\phi_{s,e}^{r_j}}\langle\boldsymbol{f}, \boldsymbol{\phi_{s,e}^{r_j}}\rangle, \boldsymbol{\epsilon}\rangle\right|}{\|\boldsymbol{\phi_{s,e}^b}\langle\boldsymbol{f}, \boldsymbol{\phi_{s,e}^b}\rangle - \boldsymbol{\phi_{s,e}^{r_j}}\langle\boldsymbol{f}, \boldsymbol{\phi_{s,e}^{r_j}}\rangle\|_2} \leq \sqrt{8\log T}\right\}.$$

The same reasoning as in the proof of Theorem 1 leads to $\mathbb{P}\left(A_T^*\right) \geq 1 - 1/(12\sqrt{\pi}T)$ and $\mathbb{P}\left(B_T^*\right) \geq 1 - 1/(12\sqrt{\pi}T)$. Therefore, Steps 1 and 2 lead to

$$\mathbb{P}\left(A_T^* \cap B_T^*\right) \geq 1 - \frac{1}{6\sqrt{\pi}T}.$$

**Step 3:** This is the main part of our proof, where we explain in detail how to get the result in (36). From now on, we assume that $A_T^*$ and $B_T^*$ both hold. The constants we use are

$$C_1 = \sqrt{\frac{2}{3}C_3^{\frac{3}{2}}} + \sqrt{8}, \quad C_2 = \frac{1}{3\sqrt{72}} - \frac{2\sqrt{2}}{C^*}, \quad C_3 = 63^{\frac{1}{3}}(2\sqrt{2}+4)^{\frac{2}{3}},$$

where $C^*$ is as in assumption (A2).

**Step 3.1:** First, $\forall j \in \{1, 2, \ldots, N\}$, we define $I_j^R$ and $I_j^L$ as in (21). At the beginning of our algorithm, $s = 1$, $e = T$ and depending on whether $r_1 \leq T - r_N$ then

$r_1$ or $r_N$ will get isolated first, respectively. W.l.o.g., assume that $r_1 \leq T - r_N$. Our aim is to first show that there will be at least an interval of the form $[1, c_{\tilde{k}}^r]$, for $\tilde{k} \in \{1, 2, \ldots, K\}$, which contains only $r_1$ and no other change-point, such that $\max_{1 \leq b < c_{\tilde{k}}^r} C_{1,c_{\tilde{k}}^r}^b > \zeta_T$. Due to ID's nature, for $K = \lceil T/\lambda_T \rceil$, then $\exists \tilde{k} \in \{1, 2, \ldots, K\}$ such that $c_{\tilde{k}}^r = \tilde{k}\lambda_T \in I_1^R$ and there is no other change-point in $[1, c_{\tilde{k}}^r]$ apart from $r_1$. We will now show that for $\tilde{b}_1 = \mathrm{argmax}_{1<t<c_{\tilde{k}}^r} C_{1,c_{\tilde{k}}^r}^t(\boldsymbol{X})$, then $C_{1,c_{\tilde{k}}^r}^{\tilde{b}_1}(\boldsymbol{X}) > \zeta_T$. Firstly, we have that

$$C_{1,c_{\tilde{k}}^r}^{\tilde{b}_1}(\boldsymbol{X}) \geq C_{1,c_{\tilde{k}}^r}^{r_1}(\boldsymbol{X}) \geq C_{1,c_{\tilde{k}}^r}^{r_1}(\boldsymbol{f}) - \sqrt{8 \log T}. \tag{37}$$

From Lemma 2, we know that $C_{1,c_{\tilde{k}}^r}^{r_1}(\boldsymbol{f}) \geq 1/(\sqrt{24}) \left(\min\left\{r_1 - 1, c_{\tilde{k}}^r - r_1\right\}\right)^{3/2} \Delta_1^f$. Now, $r_1 - 1 = r_1 - r_0 - 1 \geq \delta_T - 1 > \delta_T/3$, because for continuous piecewise-linear signals we have that $\delta_T \geq 2$ for identifiability purposes. In addition, since $c_{\tilde{k}}^r \in I_1^R$, then $c_{\tilde{k}}^r - r_1 \geq \delta_T/3$, meaning that

$$\min\left\{c_{\tilde{k}}^r - r_1, r_1 - 1\right\} \geq \frac{\delta_T}{3}. \tag{38}$$

The result in (37), the assumption (A2) and (38) yield

$$\begin{aligned}
C_{1,c_{\tilde{k}}^r}^{\tilde{b}_1}(\boldsymbol{X}) &\geq \frac{1}{\sqrt{24}}\left(\frac{\delta_T}{3}\right)^{3/2} \Delta_1^f - \sqrt{8\log T} \geq \frac{1}{\sqrt{24}}\left(\frac{\delta_T}{3}\right)^{3/2} \underline{f}_T - \sqrt{8\log T} \\
&= \delta_T^{3/2}\underline{f}_T\left(\frac{1}{3\sqrt{72}} - \frac{2\sqrt{2\log T}}{\delta_T^{3/2}\underline{f}_T}\right) \geq \left(\frac{1}{3\sqrt{72}} - \frac{2\sqrt{2}}{C^*}\right)\delta_T^{3/2}\underline{f}_T \\
&= C_2\delta_T^{3/2}\underline{f}_T > \zeta_T.
\end{aligned} \tag{39}$$

Therefore, there will be an interval of the form $[1, c_{\tilde{k}}^r]$, with $c_{\tilde{k}}^r > r_1$, such that $[1, c_{\tilde{k}}^r]$ contains only $r_1$ and $\max_{1 \leq b < c_{\tilde{k}}^r} C_{1,c_{\tilde{k}}^r}^b > \zeta_T$. Let us, for $k^* \in \{1, 2, \ldots, K\}$, denote by $c_{k^*}^r \leq c_{\tilde{k}}^r$ the first right-expanding point where this happens and let $b_1 = \mathrm{argmax}_{1 \leq t < c_{k^*}^r} C_{1,c_{k^*}^r}^t$ with $C_{1,c_{k^*}^r}^{b_1} > \zeta_T$. Note that $b_1$ can not be an estimation of any other change-point as $[1, c_{k^*}^r]$ includes only $r_1$.

Our aim now is to find $\tilde{\gamma}_T > 0$ such that for any $b^* \in \{1, 2, \ldots, c_{k^*}^r - 1\}$ with $|b^* - r_1| \left(\Delta_1^f\right)^{2/3} > \tilde{\gamma}_T$, we have

$$\left(C_{1,c_{k^*}^r}^{r_1}(\boldsymbol{X})\right)^2 > \left(C_{1,c_{k^*}^r}^{b^*}(\boldsymbol{X})\right)^2. \tag{40}$$

Proving (40) and using the definition of $b_1$ we can conclude that $|b_1 - r_1| \left(\Delta_1^f\right)^{2/3} \leq \tilde{\gamma}_T$. Since $X_t = f_t + \epsilon_t$, then (40) can be expressed as

$$\begin{aligned}
\left(C_{1,c_{k^*}^r}^{r_1}(\boldsymbol{f})\right)^2 - \left(C_{1,c_{k^*}^r}^{b^*}(\boldsymbol{f})\right)^2 &> \left(C_{1,c_{k^*}^r}^{b^*}(\boldsymbol{\epsilon})\right)^2 - \left(C_{1,c_{k^*}^r}^{r_1}(\boldsymbol{\epsilon})\right)^2 \\
&\quad + 2\left\langle \boldsymbol{\phi}_{1,c_{k^*}^r}^{b^*}\langle \boldsymbol{f}, \boldsymbol{\phi}_{1,c_{k^*}^r}^{b^*}\rangle - \boldsymbol{\phi}_{1,c_{k^*}^r}^{r_1}\langle \boldsymbol{f}, \boldsymbol{\phi}_{1,c_{k^*}^r}^{r_1}\rangle, \boldsymbol{\epsilon}\right\rangle.
\end{aligned} \tag{41}$$

W.l.o.g. assume that $b^* \geq r_1$ and a similar approach as below holds when $b^* < r_1$. We denote by

$$\Lambda := \left(C^{r_1}_{1,c^r_{k^*}}(\boldsymbol{f})\right)^2 - \left(C^{b^*}_{1,c^r_{k^*}}(\boldsymbol{f})\right)^2$$

and for the terms in the right-hand side of (41), we get that

$$\left(C^{b^*}_{1,c^r_{k^*}}(\boldsymbol{\epsilon})\right)^2 - \left(C^{r_1}_{1,c^r_{k^*}}(\boldsymbol{\epsilon})\right)^2 \leq \max_{s,e,b: s\leq b<e} \left(C^{b}_{s,e}(\boldsymbol{\epsilon})\right)^2 - \left(C^{r_1}_{1,c^r_{k}}(\boldsymbol{\epsilon})\right)^2 \leq 8\log T,$$

while from Lemma 3,

$$\begin{aligned} &2\left\langle \boldsymbol{\phi}^{\boldsymbol{b^*}}_{\boldsymbol{1},\boldsymbol{c^r_{k^*}}}\langle \boldsymbol{f}, \boldsymbol{\phi}^{\boldsymbol{b^*}}_{\boldsymbol{1},\boldsymbol{c^r_{k^*}}}\rangle - \boldsymbol{\phi}^{\boldsymbol{r_1}}_{\boldsymbol{1},\boldsymbol{c^r_{k^*}}}\langle \boldsymbol{f}, \boldsymbol{\phi}^{\boldsymbol{r_1}}_{\boldsymbol{1},\boldsymbol{c^r_{k^*}}}\rangle, \boldsymbol{\epsilon}\right\rangle \\ &\leq 2\|\boldsymbol{\phi}^{\boldsymbol{b^*}}_{\boldsymbol{1},\boldsymbol{c^r_{k^*}}} < \boldsymbol{f}, \boldsymbol{\phi}^{\boldsymbol{b^*}}_{\boldsymbol{1},\boldsymbol{c^r_{k^*}}} > - \boldsymbol{\phi}^{\boldsymbol{r_1}}_{\boldsymbol{1},\boldsymbol{c^r_{k^*}}} < \boldsymbol{f}, \boldsymbol{\phi}^{\boldsymbol{r_1}}_{\boldsymbol{1},\boldsymbol{c^r_{k^*}}} > \|_2\sqrt{8\log T} \\ &= 2\sqrt{\Lambda}\sqrt{8\log T}. \end{aligned}$$

Therefore (41) is satisfied if the stronger inequality

$$\Lambda > 8\log T + 2\sqrt{\Lambda}\sqrt{8\log T}$$

is satisfied, which has solution

$$\Lambda > (2\sqrt{2}+4)^2\log T. \tag{42}$$

Using Lemma 3, we have that (42) is implied by

$$\begin{aligned} &\frac{1}{63}\left(\min\{|r_1-b^*|, r_1-1\}\right)^3\left(\Delta^f_1\right)^2 > \left(2\sqrt{2}+4\right)^2\log T \\ &\Leftrightarrow \min\{|r_1-b^*|, r_1-1\} > \frac{(63\log T)^{1/3}(2\sqrt{2}+4)^{2/3}}{\left(\Delta^f_1\right)^{2/3}} = \frac{C_3(\log T)^{1/3}}{\left(\Delta^f_1\right)^{2/3}}. \end{aligned} \tag{43}$$

However,

$$\min\{r_1-1, c^r_{k^*}-r_1\} > 2^{1/3}C_3\frac{(\log T)^{1/3}}{\left(\Delta^f_1\right)^{2/3}} - 1 \tag{44}$$

and this is because if we assume that

$$\min\{r_1-1, c^r_{k^*}-r_1\} \leq 2^{1/3}C_3(\log T)^{1/3}/\left(\Delta^f_1\right)^{2/3} - 1$$

yields

$$\begin{aligned} C^{b_1}_{1,c^r_{k^*}}(\boldsymbol{X}) &\leq C^{r_1}_{1,c^r_{k^*}}(\boldsymbol{f}) + \sqrt{8\log T} \leq \frac{1}{\sqrt{3}}\left(\min\{r_1-1, c^r_{k^*}-r_1\}+1\right)^{3/2}\Delta^f_1 + \sqrt{8\log T} \\ &\leq \frac{1}{\sqrt{3}}\left(2^{1/3}C_3\frac{(\log T)^{1/3}}{\left(\Delta^f_1\right)^{2/3}}\right)^{3/2}\Delta^f_1 + \sqrt{8\log T} = \sqrt{\frac{2}{3}}C_3^{3/2}\sqrt{\log T} + \sqrt{8\log T} \\ &= \left(\sqrt{\frac{2}{3}}C_3^{3/2} + \sqrt{8}\right)\sqrt{\log T} = C_1\sqrt{\log T} \leq \zeta_T. \end{aligned}$$

This comes to a contradiction to $C_{1,c^r_{k^*}}^{b_1}(\boldsymbol{X}) > \zeta_T$. Therefore, (44) holds and for sufficiently large $T$,

$$\min\{r_1 - 1, c^r_{k^*} - r_1\} > 2^{1/3} C_3 \frac{(\log T)^{1/3}}{\left(\Delta_1^f\right)^{2/3}} - 1 > C_3 \frac{(\log T)^{1/3}}{\left(\Delta_1^f\right)^{2/3}}. \qquad (45)$$

From (45) we deduce that (43) is restricted to

$$|r_1 - b^*| > C_3 \frac{(\log T)^{1/3}}{\left(\Delta_1^f\right)^{2/3}},$$

which implies (40). Therefore, necessarily,

$$|b_1 - r_1| \left(\Delta_1^f\right)^{2/3} \leq C_3 (\log T)^{1/3}. \qquad (46)$$

So far, for $\lambda_T \leq \delta_T/3$ we have proven that working under the sets $A_T^*$ and $B_T^*$, there will be an interval of the form $[1, c^r_{k^*}]$, with $C_{1,c^r_{k^*}}^{b_1} > \zeta_T$, where $b_1 = \operatorname*{argmax}_{1 \leq t < c^r_{k^*}} C_{1,c^r_{k^*}}^t$ is an estimation of $r_1$ that satisfies (46).

**Step 3.2:** After detecting the first change-point, ID follows the same process as in Step 3.1 in the set $[c^r_{k^*}, T]$, which contains $r_2, r_3, \ldots, r_N$. This means that we do not check for possible change-points in the interval $[b_1 + 1, c^r_{k^*})$. Therefore, we need to prove that:

(S.1) There is no other change-point in $[b_1 + 1, c^r_{k^*})$, apart from possibly the already detected $r_1$;
(S.2) $c^r_{k^*}$ is at a location which allows for detection of $r_2$.

**For (S.1):** The approach is the same as the one in Step 3.2 in the proof of Theorem 1 and will not be repeated here.

Similarly to the approach in Step 3.1, our method applied now to $[c^r_{k^*}, T]$, will first detect $r_2$ or $r_N$ depending on whether $r_2 - c^r_{k^*}$ is smaller or larger than $T - r_N$. If $T - r_N < r_2 - c^r_{k^*}$ then $r_N$ will get isolated first and the procedure to show its detection is exactly the same as in Step 3.1 where we explained the detection of $r_1$. Therefore, w.l.o.g. and also for the sake of showing (S.2) let us assume that $r_2 - c^r_{k^*} \leq T - r_N$.

**For (S.2):** With $\mathrm{R}_{s,e}$ as in (2), there exists $c^r_{k_2} \in \mathrm{R}_{c^r_{k^*},T}$ such that $c^r_{k_2} \in I_2^R$. We will show that $r_2$ gets detected in $[c^r_{k^*}, c^r_{k_2^*}]$, for $k_2^* \leq k_2$ and its detection is $b_2 = \operatorname{argmax}_{c^r_{k^*} \leq t < c^r_{k_2^*}} C^t_{c^r_{k^*}, c^r_{k_2^*}}(\boldsymbol{X})$, which satisfies $|b_2 - r_2| \left(\Delta_2^f\right)^{2/3} \leq C_3 (\log T)^{1/3}$. Using again Lemma 3 and for $\tilde{b}_2 = \operatorname{argmax}_{c^r_{k^*} \leq t < c^r_{k_2}} C^t_{c^r_{k^*}, c^r_{k_2}}$, we have that

$$\begin{aligned} C^{\tilde{b}_2}_{c^r_{k^*}, c^r_{k_2}}(\boldsymbol{X}) &\geq C^{r_2}_{c^r_{k^*}, c^r_{k_2}}(\boldsymbol{X}) \geq C^{r_2}_{c^r_{k^*}, c^r_{k_2}}(\boldsymbol{f}) - \sqrt{8 \log T} \\ &\geq \frac{1}{\sqrt{24}} \left(\min\left\{r_2 - c^r_{k^*}, c^r_{k_2} - r_2\right\}\right)^{3/2} \Delta_2^f - \sqrt{8 \log T}. \qquad (47) \end{aligned}$$

By construction,

$$c^r_{k_2} - r_2 \geq \frac{\delta_T}{3}$$
$$r_2 - c^r_{k^*} \geq r_2 - c^r_k = r_2 - r_1 - (c^r_k - r_1) \geq \delta_T - (c^r_k - r_1) > \delta_T - 2\frac{\delta_T}{3} = \frac{\delta_T}{3},$$

which means that $\min\left\{c^r_{k_2} - r_2, r_2 - c^r_{k^*}\right\} \geq \delta_T/3$. Therefore, continuing from (47) and using the exact same calculations as in (39), we have that

$$C^{\tilde{b}_2}_{c^r_{k^*}, c^r_{k_2}}(\boldsymbol{X}) \geq C_2 \delta_T^{3/2} \underline{f}_T > \zeta_T.$$

Therefore, for a $c^r_{k_2} \in \mathrm{R}_{c^r_{k^*}, T}$ we have shown that there exists an interval of the form $[c^r_{k^*}, c^r_{\tilde{k}_2}]$, with $\max_{c^r_{k^*} \leq b < c^r_{\tilde{k}_2}} C^b_{c^r_{k^*}, c^r_{\tilde{k}_2}} > \zeta_T$. Let us denote by $c^r_{k_2^*} \in \mathrm{R}_{c^r_{k^*}, T}$ the first right-expanding point where this occurs and let $b_2 = \mathrm{argmax}_{c^r_{k^*} \leq t < c^r_{k_2^*}} C^t_{c^r_{k^*}, c^r_{k_2^*}}$ with $C^{b_2}_{c^r_{k^*}, c^r_{k_2^*}} > \zeta_T$. We will now show that $|b_2 - r_2| \left(\Delta^f_2\right)^{2/3} \leq C_3 (\log T)^{1/3}$. Following the same process as in Step 3.1 and assuming now that $b_2 < r_2$, we have that for $b^* \in \left\{c^r_{k^*}, c^r_{k^*} + 1, \ldots, c^r_{k_2^*} - 1\right\}$,

$$\left(C^{r_2}_{c^r_{k^*}, c^r_{k_2^*}}(\boldsymbol{X})\right)^2 > \left(C^{b^*}_{c^r_{k^*}, c^r_{k_2^*}}(\boldsymbol{X})\right)^2 \tag{48}$$

is implied by $\min\left\{|b^* - r_2|, c^r_{k_2} - r_2\right\} > C_3 (\log T)^{1/3} / \left(\Delta^f_2\right)^{2/3}$. However, following the same procedure as in Step 3.1 we can show that for sufficiently large $T$,

$$\min\left\{c^r_{k_2} - r_2, r_2 - c^r_k\right\} > C_3 \frac{(\log T)^{1/3}}{\left(\Delta^f_2\right)^{2/3}}.$$

Thus, (48) is implied by $|b^* - r_2| \left(\Delta^f_2\right)^{2/3} > C_3 (\log T)^{1/3}$. Therefore, $|b_2 - r_2| \left(\Delta^f_2\right)^{2/3} > C_3 (\log T)^{1/3}$ would necessarily mean that $C^{r_2}_{c^r_{k^*}, c^r_{k_2^*}}(\boldsymbol{X}) > C^{b_2}_{c^r_{k^*}, c^r_{k_2^*}}(\boldsymbol{X})$, which is not true by the definition of $b_2$. Having said this, we conclude that $|b_2 - r_2| \left(\Delta^f_2\right)^{2/3} \leq C_3 (\log T)^{1/3}$.

Having detected $r_2$, then our algorithm will proceed in the interval $[s, e] = [c^r_{k_2^*}, T]$ and all the change-points will get detected one by one since Step 3.2 will be applicable as long as there are previously undetected change-points in $[s, e]$. Denoting by $\hat{r}_j$ the estimation of $r_j$ as we did in the statement of the theorem, then we conclude that all change-points will first get isolated and then detected one by one and

$$|\hat{r}_j - r_j| \left(\Delta^f_j\right)^{2/3} \leq C_3 (\log T)^{1/3}, \quad \forall j \in \{1, 2, \ldots, N\}.$$

**Step 4:** The arguments given in Steps 1-3 hold in $A_T^* \cap B_T^*$. At the beginning of the algorithm, $s = 1, e = T$ and for $N \geq 1$, there exist $k_1 \in \{1, 2, \ldots, K\}$ such that $s_{k_1} = s, e_{k_1} \in I_1^R$ and $k_2 \in \{1, 2, \ldots, K\}$ such that $s_{k_2} \in I_N^L, e_{k_2} = e$. As in our previous steps, w.l.o.g. assume that $r_1 \leq T - r_N + 1$, meaning that $r_1$ gets isolated and detected first in an interval $[s, c_{k^*}^r]$, where $c_{k^*}^r \in \mathrm{R}_{1,T}$ and it is less than or equal to $e_{k_1}$. Then, $\hat{r}_1 = \mathrm{argmax}_{s \leq t < c_{k^*}^r} C_{s,c_{k^*}^r}^t(\boldsymbol{X})$ is the estimated location for $r_1$ and $|r_1 - \hat{r}_1| \left(\Delta_1^f\right)^{2/3} \leq C_3 (\log T)^{1/3}$. After this, the algorithm continues in $[c_{k^*}^r, T]$ and keeps detecting all the change-points as explained in Step 3. It is important to note that there will not be any double detection issues because naturally, at each step of the algorithm, the new interval $[s, e]$ does not include any previously detected change-points.

Once all the change-points have been detected one by one, then $[s, e]$ will have no other change-points in it. Our method will keep interchangeably checking for possible change-points in intervals of the form $\left[s, c_{\tilde{k}_1}^r\right]$ and $\left[c_{\tilde{k}_2}^l, e\right]$ for $c_{\tilde{k}_1}^r \in \mathrm{R}_{s,e}$ and $c_{\tilde{k}_2}^l \in \mathrm{L}_{s,e}$. Allow us to denote by $[s^*, e^*]$ any of these intervals. Our algorithm will not detect anything in $[s^*, e^*]$ since $\forall b \in [s^*, e^*)$,

$$C_{s^*,e^*}^b(\boldsymbol{X}) \leq C_{s^*,e^*}^b(\boldsymbol{f}) + \sqrt{8 \log T} = \sqrt{8 \log T} < C_1 \sqrt{\log T} \leq \zeta_T.$$

After not detecting anything in all intervals of the above form, then the algorithm concludes that there are not any change-points in $[s, e]$ and stops. ■

**Brief discussion of the steps of the proof of Theorem 3**

Before the thorough mathematical proof of Theorem 3, we provide an informal explanation of the three main steps in our proof. The notation is as in the main paper with $\tilde{S}$ denoting the ordered set with the remaining and relabelled estimated change-points, $\tilde{r}_k$, after each estimation is removed. At the beginning of the change-point removal approach, $\tilde{S} = [\tilde{r}_1, \tilde{r}_2, \ldots, \tilde{r}_J]$. In Step 1 of the proof, we show that for each true change-point $r_j, j \in \{1, 2, \ldots, N\}$, there is at least one and at most four estimated change-points, $\tilde{r}_k, k \in \{1, 2, \ldots, J\}$ within a distance equal to $\tilde{C}(\log T)^{\alpha} / \left(\Delta_j^f\right)^2$, where $\tilde{C} > 0$. In Step 2, we show that there are at most two estimated change-points between two consecutive true change-points. In Step 3, we prove that as the algorithm proceeds, then $\forall j \in \{1, 2, \ldots, N\}$, the only remaining change-point for $r_j$ is within a distance of $C_1(\log T)^{\alpha} / \left(\Delta_j^f\right)^2$ from $r_j$ and it cannot be removed whilst there are still more than $N$ estimated change-points in $\tilde{S}$. Step 4 shows that the sSIC penalty as defined in (11), proposes a solution with $\hat{N} = N$ estimated change-points.

**Proof of Theorem 3**

Allow us first to denote by

$$D_T = \left\{ \max_{s,b,e: 1 \leq b \leq e \leq T} \left| \frac{1}{\sqrt{e - b + 1}} \sum_{t=b}^{e} \epsilon_t \right| \leq \sqrt{6 \log T} \right\}. \tag{49}$$

We will show that $\mathbb{P}\,(D_T) \geq 1-\sqrt{2}/(\sqrt{\pi}T)$. For $Z \sim \mathcal{N}(0,1)$, using the Bonferroni inequality we get that

$$\begin{aligned}\mathbb{P}\,((D_T)^c) &= \mathbb{P}\left(\max_{s,b,e:1\leq b\leq e\leq T}\left|\frac{1}{\sqrt{e-b+1}}\sum_{t=b}^{e}\epsilon_t\right| > \sqrt{6\log T}\right)\\ &\leq \sum_{1\leq b\leq e\leq T}\mathbb{P}\left(|Z| > \sqrt{6\log T}\right) \leq T^2\mathbb{P}\left(|Z| > \sqrt{6\log T}\right)\\ &= 2T^2\mathbb{P}\left(Z > \sqrt{6\log T}\right) \leq 2T^2\frac{\phi(\sqrt{6\log T})}{\sqrt{6\log T}} \leq \frac{\sqrt{2}}{\sqrt{\pi}T},\end{aligned}$$

where $\phi(\cdot)$ is the probability density function of the standard normal distribution. Therefore, $\mathbb{P}\,(D_T) \geq 1-\sqrt{2}/(\sqrt{\pi}T)$. With $A_T$ as in (18), the work that follows is valid on the set $A_T \cap D_T$ with $\mathbb{P}\,(A_T \cap D_T) \geq 1 - 1/(12\sqrt{\pi}T) - \sqrt{2}/(\sqrt{\pi}T)$. We take $\tilde{\tilde{C}}$ from the main paper to be equal to $2\sqrt{2}$.

**Step 1**: When the algorithm moves from Part 1 to Part 2, as described in Subsection 3.3, then we are under a structure described by the following three characteristics:

**(P1)** For $\tilde{C} > 0$, there is at least one estimation within a distance of $\tilde{C}(\log T)^\alpha/\left(\Delta_j^f\right)^2$ from $r_j$, $\forall j \in \{1,2,\ldots,N\}$. We know that this is true at the beginning of Part 1 due to calling the ID algorithm with threshold $\zeta_T$. This continues to be the case when the algorithm proceeds to Part 2, because if $\tilde{r}_k$ is the last estimation within $\tilde{C}(\log T)^\alpha/\left(\Delta_j^f\right)^2$ from $r_j$, then $\tilde{r}_{k+1}-\tilde{r}_{k-1} > 2\tilde{C}(\log T)^\alpha/\left(\Delta_j^f\right)^2 = 2C^*(\log T)^\alpha$ and $\tilde{r}_k$ cannot be removed in Part 1.

**(P2)** For each $j = 1,2,\ldots,N$, there are at most four estimated change-points within a distance of $\tilde{C}(\log T)^\alpha/\left(\Delta_j^f\right)^2$ from $r_j$. We can not have more than four estimations as if this was the case then at least three of them, let's denote them by $p_1, p_2, p_3$, would be either on the right or the left of $r_j$, which would then mean that both $CS(p_2) \leq 2\sqrt{2\log T}$ and $p_3 - p_1 \leq \tilde{C}(\log T)^\alpha/\left(\Delta_j^f\right)^2 = C^*(\log T)^\alpha$ are satisfied and therefore $p_2$ would have been removed in Part 1 of the algorithm as explained in Subsection 3.3 of the paper.

**(P3)** There is an unknown, possibly large, number of estimated change-points (which tends to infinity as $T$ goes to infinity) between any two true change-points, namely $r_j$ and $r_{j+1}$. This issue is solved in Part 2 of the algorithm.

**Step 2:** We are now in Part 2 of the algorithm as explained in Subsection 3.3, which guarantees that the minimum distance between two estimated change-points is $C^*(\log T)^\alpha$, and also that there exists $C \geq \tilde{C}$, such that there is at least one estimation within a distance of $C(\log T)^\alpha/\left(\Delta_j^f\right)^2$ from $r_j$. After that, in Part 2 we collect the triplets $(\tilde{r}_{j-1}, \tilde{r}_j, \tilde{r}_{j+1})$ and we calculate $\mathrm{CS}(\tilde{r}_j)$ and for $m = \mathrm{argmin}_j\,\{\mathrm{CS}(\tilde{r}_j)\}$, if $CS(\tilde{r}_m) \leq 2\sqrt{2\log T}$, then $\tilde{r}_m$ is removed and the process is repeated for the remaining estimated change-points. By doing this it is easy to see that, first of all, between $r_j$ and $r_{j+1}$, $j = 0,1,\ldots,N$ there will be at most two estimated change-points, since if there were more, then we would have triplets $(\tilde{r}_{j-1}, \tilde{r}_j, \tilde{r}_{j+1})$ with

$\mathrm{CS}(\tilde{r}_j) \leq 2\sqrt{2\log T}$ and $\tilde{r}_j$ would have been removed. Secondly, for each $j = 1, 2, \ldots, N$ there is still at least an estimation within a distance of $C(\log T)^\alpha / \left(\Delta_j^f\right)^2$ from $r_j$. If there is exactly one estimated change-point in the area $r_j \pm C(\log T)^\alpha / \left(\Delta_j^f\right)^2$, namely $\tilde{r}_k$, then this cannot be removed in Part 2 of the algorithm because $\min\{\tilde{r}_k - \tilde{r}_{k-1}, \tilde{r}_{k+1} - \tilde{r}_k\} > C^*(\log T)^\alpha$ and therefore for $C_4 > 0$,

$$\begin{aligned} \left|\tilde{X}^{\tilde{r}_k}_{\tilde{r}_{k-1},\tilde{r}_{k+1}}\right| &\geq \left|\tilde{f}^{\tilde{r}_k}_{\tilde{r}_{k-1},\tilde{r}_{k+1}}\right| - 2\sqrt{2\log T} \\ &\geq C_4(\log T)^{\alpha/2} - 2\sqrt{2\log T}, \end{aligned}$$

which for sufficiently large $T$ is greater than $2\sqrt{2\log T}$. Therefore, $\tilde{r}_k$ will not be removed and there is at least one estimation within a distance of $C(\log T)^\alpha / \left(\Delta_j^f\right)^2$ from $r_j$, $\forall j = 1, 2, \ldots, N$. This estimation will be in the set $\tilde{S}$ of estimated change-points that continue in Part 3 of the algorithm.

**Step 3:** We are in Part 3 of the algorithm as explained in Subsection 3.3. In this step, we will show that with $m = \mathrm{argmin}_{\tilde{r}_k \in \tilde{S}}\ CS(\tilde{r}_k)$, then once $CS(\tilde{r}_m) > 2\sqrt{2\log T}$ we will be at the stage where $\tilde{S}$ contains $N$ estimated change-points; one estimated change-point within a distance of $C_1(\log T)^\alpha / \left(\Delta_j^f\right)^2$ from each $r_j$, $\forall j \in \{1, 2, \ldots, N\}$, where $C_1 > 0$. W.l.o.g. let $\tilde{r}_m$ be between $r_j$ and $r_{j+1}$. From Step 2 we know that there is a finite number (no more than four) of estimated change-points in $[r_{j-1}, r_{j+1}]$. It is straightforward that at the beginning of Part 3 we have at most $2N$ estimations and either of the following two cases is possible:

**Case 1:** $\tilde{r}_m$ is not the closest change-point to either the true change-point on its left ($r_j$ for a $j \in \{1, 2, \ldots, N\}$) or the true change-point on its right ($r_{j+1}$). Since, $\tilde{s}_m = \lfloor(\tilde{r}_{m-1} + \tilde{r}_m)/2\rfloor + 1$ and $\tilde{e}_m = \lceil(\tilde{r}_m + \tilde{r}_{m+1})/2\rceil$, it is straightforward to see that necessarily $\tilde{s}_m$ is on the right of $r_j$ and $\tilde{e}_m$ is on the left of $r_{j+1}$. This means that there are not any true change-points in $[\tilde{s}_m, \tilde{e}_m]$ and because we are working in the set $A_T$, we have that

$$CS(\tilde{r}_m) = \left|X^{\tilde{r}_m}_{\tilde{s}_m,\tilde{e}_m}\right| = \left|X^{\tilde{r}_m}_{\tilde{s}_m,\tilde{e}_m} - f^{\tilde{r}_m}_{\tilde{s}_m,\tilde{e}_m}\right| \leq 2\sqrt{2\log T}.$$

Therefore, $\tilde{r}_m$ will be removed from the set $\tilde{S}$.

**Case 2:** $\tilde{r}_m$ is the closest change-point to a true change-point, namely $r_j$ for a $j \in \{1, 2, \ldots, N\}$, and from what has been discussed in Steps 1 and 2, $\tilde{r}_m$ is within a distance of $C(\log T)^\alpha / \left(\Delta_j^f\right)^2$. If $CS(\tilde{r}_m) \leq 2\sqrt{2\log T}$, then there is at least another estimated change-point within a distance of $C_m(\log T)^\alpha / \left(\Delta_j^f\right)^2$ from $\tilde{r}_m$ (and therefore from $r_j$ too), where $C_m$ is a constant that does not depend on $T$. If this was not the case, then since we are working under $A_T$,

$$\begin{aligned} \left|\tilde{X}^{\tilde{r}_m}_{\tilde{s}_m,\tilde{e}_m}\right| &\geq \left|\tilde{f}^{\tilde{r}_m}_{\tilde{s}_m,\tilde{e}_m}\right| - 2\sqrt{2\log T} \\ &\geq C_m^*(\log T)^\alpha - 2\sqrt{2\log T} > 2\sqrt{2\log T} \end{aligned}$$

for a constant $C_m^*$ and for sufficiently large $T$. Therefore, $\tilde{r}_m$ would not get removed. Since there are at most $2N$ estimations, then the constants $C_m$ are upper bounded by a general constant $C_1$ and therefore, when Part 3 terminates, each true change-point will have only one estimated change-point within the distance of $C_1(\log T)^\alpha / \left(\Delta_j^f\right)^2$. This is exactly the stage in our algorithm, where $J = N$, with $J$ denoting the number of estimated change-points in the set $\tilde{S}$. There are not any change-point identifiability issues, because $(\log T)^\alpha = o\left(\delta_T \underline{f}_T^2\right)$ and for $T$ large enough

$$2C_1(\log T)^\alpha < \delta_T \underline{f}_T^2.$$

The algorithm will then proceed to Part 4 explained in Subsection 3.3 and each of the remaining estimated change-points will be removed one by one until $\tilde{S}$ is the empty set.

**Step 4:** In this last step we will prove that the sSIC penalty indicates the solution obtained when Part 3 terminates, where the number of estimated change-points is equal to $N$. We have already explained in Section 3.3 of the paper that in the scenario of piecewise-constant mean signals,

$$\text{sSIC}(j) = \frac{T}{2}\log\hat{\sigma}_j^2 + (j+1)(\log T)^\alpha, \tag{50}$$

where for any candidate model $\mathcal{M}_j, j = 0,1,\ldots,J$, we have $\hat{f}_t^j = (\hat{r}_{j+1} - \hat{r}_j)^{-1}\sum_{k=r_j+1}^{\hat{r}_{j+1}} X_j$, for $\hat{r}_j + 1 \leq t \leq \hat{r}_{j+1}$, and $\hat{\sigma}_j^2 = T^{-1}\sum_{t=1}^T (X_t - \hat{f}_t^j)^2$ is the maximum likelihood estimator of the residual variance associated with model $\mathcal{M}_j$. It has also been proven in Fryzlewicz (2014) that in an interval $[s,e]$,

$$\hat{\sigma}_{j-1}^2 - \hat{\sigma}_j^2 = \frac{\left(\tilde{X}_{s,e}^d\right)^2}{T},$$

where $d \in \{s, s+1, \ldots, e-1\}$. With $j = 0,1,\ldots,J$ the number of estimated change-points related to $\mathcal{M}_j$, if $j > N$, it means that all the change-points have been detected (see explanation in Step 2) and therefore $\left|\tilde{X}_{s,e}^d\right| \leq 2\sqrt{2\log T}$ since we are working under $A_T$. Therefore, $\hat{\sigma}_{j-1}^2 - \hat{\sigma}_j^2 \leq 8\log T/T$. In addition, since we are working in the set $D_T$, we have that $\left|\hat{\sigma}_N^2 - \sigma^2\right| \leq C^* \log T/T$, for a positive constant $C^*$. Using these results, the definition of sSIC$(j)$ in (50), and a first order Taylor expansion, we conclude that

$$\begin{aligned}
\text{sSIC}(j) - \text{sSIC}(N) &= \frac{T}{2}\log\frac{\hat{\sigma}_j^2}{\hat{\sigma}_N^2} + (j-N)(\log T)^\alpha \\
&= \frac{T}{2}\log\left(1 - \frac{\hat{\sigma}_N^2 - \hat{\sigma}_j^2}{\hat{\sigma}_N^2}\right) + (j-N)(\log T)^\alpha \\
&\geq -\frac{T}{2}(1+w)\frac{\hat{\sigma}_N^2 - \hat{\sigma}_j^2}{\hat{\sigma}_N^2} + (j-N)(\log T)^\alpha \\
&\geq -K_1\log T + (j-N)(\log T)^\alpha,
\end{aligned} \tag{51}$$

where $K_1$ and $w$ are positive constants. The lower bound in (51) is positive for $T$ large enough. Now, if $j < N$, then from the proof of Theorem 1, we know that in the interval $[s, e]$, we have that $\left|\tilde{X}^d_{s,e}\right| \geq \tilde{C}_2\sqrt{\delta_T}\underline{f}_T$, leading to $\hat{\sigma}^2_{j-1} - \hat{\sigma}^2_j \geq \tilde{C}_2^2\delta_t\underline{f}_T^2/T$. Therefore,

$$\begin{aligned}
\text{sSIC}(j) - \text{sSIC}(N) &= \frac{T}{2}\log\frac{\hat{\sigma}^2_j}{\hat{\sigma}^2_N} + (j-N)(\log T)^\alpha \\
&= \frac{T}{2}\log\left(1 + \frac{\hat{\sigma}^2_j - \hat{\sigma}^2_N}{\hat{\sigma}^2_N}\right) + (j-N)(\log T)^\alpha \\
&\geq -\frac{T}{2}(1-w_2)\frac{\hat{\sigma}^2_j - \hat{\sigma}^2_N}{\hat{\sigma}^2_N} + (j-N)(\log T)^\alpha \\
&\geq K_2\delta_T\underline{f}_T^2 + (j-N)(\log T)^\alpha, \qquad (52)
\end{aligned}$$

where $K_2$ and $w_2$ are positive constants. The lower bound in (52) is positive for $T$ large enough because $(\log T)^\alpha = o\left(\delta_T\underline{f}_T^2\right)$. The results in (51) and (52) show that for $T$ large enough and on the set $A_T \cap D_T$, we have that $\text{sSIC}(j) > \text{sSIC}(N)$, for $j \neq N$. Therefore, $\text{sSIC}(j)$ is minimized for $j = N$, showing that $\hat{N} = N$. ■

**Proof of Corollary 1.** From now on, we denote by

$$\tilde{\epsilon}^b_{s,e} = \sqrt{\frac{e-b}{n(b-s+1)}}\sum_{t=s}^{b}\epsilon_t - \sqrt{\frac{b-s+1}{n(e-b)}}\sum_{t=b+1}^{e}\epsilon_t,$$

where $1 \leq s \leq b < e \leq T$ and $n = e - s + 1$. Using also the notation in (14), we have

$$\begin{aligned}
&\exists_{s,b,e} \left\{ \left(\tilde{\epsilon}^b_{s,e}\right)^2 > 3\gamma \right\} \\
&\iff \exists_{s,b,e} \left\{ \frac{e-b}{n} \left(\tilde{\tilde{\epsilon}}_{s,b}\right)^2 + \frac{b-s+1}{n} \left(\tilde{\tilde{\epsilon}}_{b+1,e}\right)^2 \right. \\
&\qquad\qquad \left. -2\frac{\{(e-b)(b-s+1)\}^{1/2}}{n} \tilde{\tilde{\epsilon}}_{s,b}\tilde{\tilde{\epsilon}}_{b+1,e} > 3\gamma \right\} \\
&\implies \exists_{s,b,e} \left\{ \frac{e-b}{n} \left(\tilde{\tilde{\epsilon}}_{s,b}\right)^2 + \frac{b-s+1}{n} \left(\tilde{\tilde{\epsilon}}_{b+1,e}\right)^2 \right. \\
&\qquad\qquad \left. +2\frac{\{(e-b)(b-s+1)\}^{1/2}}{n} |\tilde{\tilde{\epsilon}}_{s,b}||\tilde{\tilde{\epsilon}}_{b+1,e}| > 3\gamma \right\} \\
&\implies \exists_{s,b,e} \left\{ \frac{e-b}{n} \left(\tilde{\tilde{\epsilon}}_{s,b}\right)^2 > \frac{e-b}{n} 2\gamma \right\} \\
&\quad \vee\ \exists_{s,b,e} \left\{ \frac{b-s+1}{n} \left(\tilde{\tilde{\epsilon}}_{b+1,e}\right)^2 > \frac{b-s+1}{n} 2\gamma \right\} \\
&\quad \vee\ \exists_{s,b,e} \left\{ 2\frac{\{(e-b)(b-s+1)\}^{1/2}}{n} |\tilde{\tilde{\epsilon}}_{s,b}||\tilde{\tilde{\epsilon}}_{b+1,e}| > \gamma \right\} \\
&\implies \exists_{s,b} \left\{ \left(\tilde{\tilde{\epsilon}}_{s,b}\right)^2 > 2\gamma \right\} \vee \exists_{b,e} \left\{ \left(\tilde{\tilde{\epsilon}}_{b+1,e}\right)^2 > 2\gamma \right\} \\
&\quad \vee\ \exists_{s,b,e} \left\{ 2\frac{\{(e-b)(b-s+1)\}^{1/2}}{n} \tilde{\tilde{\epsilon}}_{s,b}\tilde{\tilde{\epsilon}}_{b+1,e} > \gamma \right\} \\
&\quad \vee\ \exists_{s,b,e} \left\{ -2\frac{\{(e-b)(b-s+1)\}^{1/2}}{n} \tilde{\tilde{\epsilon}}_{s,b}\tilde{\tilde{\epsilon}}_{b+1,e} > \gamma \right\}. \qquad (53)
\end{aligned}$$

Define

$$\gamma' = \frac{\gamma n}{2\{(e-b)(b-s+1)\}^{1/2}},$$

and consider any straight line in the $(\tilde{\tilde{\epsilon}}_{s,b}, \tilde{\tilde{\epsilon}}_{b+1,e})$-plane, defined by the equation $\alpha\tilde{\tilde{\epsilon}}_{s,b} + \beta\tilde{\tilde{\epsilon}}_{b+1,e} = \lambda$, that is tangent to the curve $\tilde{\tilde{\epsilon}}_{s,b}\tilde{\tilde{\epsilon}}_{b+1,e} = \gamma'$ in the quadrant $(\tilde{\tilde{\epsilon}}_{s,b}, \tilde{\tilde{\epsilon}}_{b+1,e}) > (0,0)$. By elementary calculus, which we do not repeat here, the tangency is attained when $\lambda = 2\{\gamma'\alpha\beta\}^{1/2}$. By elementary geometry,

$$\tilde{\tilde{\epsilon}}_{s,b}\tilde{\tilde{\epsilon}}_{b+1,e} > \gamma' \implies |\alpha\tilde{\tilde{\epsilon}}_{s,b} + \beta\tilde{\tilde{\epsilon}}_{b+1,e}| > 2\{\gamma'\alpha\beta\}^{1/2}. \qquad (54)$$

Consider the particular values of $(\alpha, \beta)$ given by

$$\alpha = \left\{\frac{b-s+1}{n}\right\}^{1/2} \qquad \beta = \left\{\frac{e-b}{n}\right\}^{1/2},$$

and note that by the definition of $\tilde{\tilde{\epsilon}}_{s,e}$ we have

$$\alpha\tilde{\tilde{\epsilon}}_{s,b} + \beta\tilde{\tilde{\epsilon}}_{b+1,e} = \left\{\frac{b-s+1}{n}\right\}^{1/2} \tilde{\tilde{\epsilon}}_{s,b} + \left\{\frac{e-b}{n}\right\}^{1/2} \tilde{\tilde{\epsilon}}_{b+1,e} = \tilde{\tilde{\epsilon}}_{s,e}. \qquad (55)$$

Therefore, from the definitions of $\gamma'$, $\alpha$ and $\beta$, the implication in (54) and the identity in (55), we have

$$2\frac{\{(e-b)(b-s+1)\}^{1/2}}{n}\tilde{\tilde{\epsilon}}_{s,b}\tilde{\tilde{\epsilon}}_{b+1,e} > \gamma \iff \tilde{\tilde{\epsilon}}_{s,b}\tilde{\tilde{\epsilon}}_{b+1,e} > \gamma'$$
$$\implies |\tilde{\tilde{\epsilon}}_{s,e}| > 2\{\gamma'\alpha\beta\}^{1/2} \iff |\tilde{\tilde{\epsilon}}_{s,e}| > \{2\gamma\}^{1/2} \iff \left(\tilde{\tilde{\epsilon}}_{s,e}\right)^2 > 2\gamma. \quad (56)$$

Considering again (53), this implies

$$\exists_{s,b,e}\ \left\{\left(\tilde{\epsilon}^b_{s,e}\right)^2 > 3\gamma\right\}$$
$$\implies \exists_{s,b}\left\{\left(\tilde{\tilde{\epsilon}}_{s,b}\right)^2 > 2\gamma\right\} \vee \exists_{s,b,e}\left\{-2\frac{\{(e-b)(b-s+1)\}^{1/2}}{n}\tilde{\tilde{\epsilon}}_{s,b}\tilde{\tilde{\epsilon}}_{b+1,e} > \gamma\right\}. \quad (57)$$

By (15), we have

$$P\left(\exists_{s,b,e}\left\{-2\frac{\{(e-b)(b-s+1)\}^{1/2}}{n}\tilde{\tilde{\epsilon}}_{s,b}\tilde{\tilde{\epsilon}}_{b+1,e} > \gamma\right\}\right)$$
$$\le P\left(\exists_{s,b,e}\left\{2\frac{\{(e-b)(b-s+1)\}^{1/2}}{n}\tilde{\tilde{\epsilon}}_{s,b}\tilde{\tilde{\epsilon}}_{b+1,e} > \gamma\right\}\right). \quad (58)$$

From (56), we have

$$P\left(\exists_{s,b,e}\left\{2\frac{\{(e-b)(b-s+1)\}^{1/2}}{n}\tilde{\tilde{\epsilon}}_{s,b}\tilde{\tilde{\epsilon}}_{b+1,e} > \gamma\right\}\right) \le P\left(\exists_{s,e}\left(\tilde{\tilde{\epsilon}}_{s,e}\right)^2 > 2\gamma\right) \quad (59)$$

Therefore, from (57), and using (58) and (59) in turn, we have

$$P(\exists_{s,b,e}\ \left(\tilde{\epsilon}^b_{s,e}\right)^2 > 3\gamma) \le P(\exists_{s,e}\left(\tilde{\tilde{\epsilon}}_{s,e}\right)^2 > 2\gamma)$$
$$+ P\left(\exists_{s,b,e}\left\{2\frac{\{(e-b)(b-s+1)\}^{1/2}}{n}\tilde{\tilde{\epsilon}}_{s,b}\tilde{\tilde{\epsilon}}_{b+1,e} > \gamma\right\}\right)$$
$$\le 2P(\exists_{s,e}\left(\tilde{\tilde{\epsilon}}_{s,e}\right)^2 > 2\gamma), \quad (60)$$

Now, for any $\delta > 0$, taking $\gamma = \sigma^2(1+\delta)\log T$, we have that

$$P\left(\exists_{s,b,e}\ \left(\tilde{\epsilon}^b_{s,e}\right)^2 > 3\sigma^2(1+\delta)\log T\right) \le 2P\left(\exists_{s,e}\ \left(\tilde{\tilde{\epsilon}}_{s,e}\right)^2 > 2\sigma^2(1+\delta)\log T\right),$$

and the statement is a consequence of Lemma 1 in Yao (1988). ■

## References


Baranowski, R., Chen, Y. and Fryzlewicz, P. (2019). Narrowest-over-threshold detection of multiple change points and change-point-like features. *Journal of the Royal Statistical Society Series B*, **81**, 649–672.

Fryzlewicz, P. (2014). Wild binary segmentation for multiple change-point detection. *Annals of Statistics* **42**, 2243–2281.

Yao, Y.-C. (1988). Estimating the number of change-points via Schwarz' criterion. *Statistics & Probability Letters*, **6**, 181–189.
